# A Revision of the Stylasteridae (Cnidaria, Hydrozoa, Filifera) from Alaska and Adjacent Waters

**DOI:** 10.3897/zookeys.158.1910

**Published:** 2011-12-22

**Authors:** Stephen D. Cairns, Alberto Lindner

**Affiliations:** 1 Department of Invertebrate Zoology, National Museum of Natural History, Smithsonian Institution, Washington DC 20560, USA; 2 Departmento de Ecologia e Zoologia, Universidade Federal de Santa Catarina, Florianópolis, SC 88040-970, Brazil

**Keywords:** Stylasteridae, Hydrozoa, Cnidaria, Aleutian Islands, Alaska, British Columbia, *Stylaster*, *Errinopora*

## Abstract

The stylasterid fauna of Alaska is revised, consisting of the description or redescription and illustration of 21 species, one additional subspecies, and a geographically adjacent species: *Stylaster venustus*. Six new species and one new subspecies are described: *Errinopora fisheri*, *Errinopora undulata*, *Errinopora disticha*, *Errinopora dichotoma*, *Stylaster crassiseptum*, *Stylaster repandus*, and *Stylaster parageus columbiensis*. Four subspecies are raised to species rank: *Stylaster leptostylus*, *Stylaster trachystomus*, *Stylaster parageus*, and *Distichopora japonica*, and five species and one subspecies were synonymized. A dichotomous key to the *Errinopora* species and tabular keys to the *Errinopora* and Alaskan *Stylaster* species are provided. The focus of the study was on the stylasterids from Alaska, primarily those from the diverse Aleutian Islands, but also including records from British Columbia. This is the first revisionary work on this fauna since the seminal report by Fisher in 1938.

## Introduction

The history of the published information about Alaskan stylasterids is not long. The first stylasterids to be reported from off Alaska were beach-worn specimens or coralla obtained from very shallow water from the Aleutian Islands and the Alaskan Peninsula (Dall 1884): *Stylaster verrillii* (Dall, 1884) and *Stylantheca papillosa* (Dall, 1884). Dall’s report also included a description of *Stylaster moseleyi* (Dall, 1884), which is now considered to be a junior synonym of *Stylaster verrillii*. Broch (1936) later reported *Stylaster boreopacificus typica* from Yukatat Bay, which is herein reidentified as *Stylaster parageus parageus*.

The major contributor to this fauna, however, was W. K. Fisher (1938), who, based on specimens from 19 stations from the Pacific Expeditions of the US Fish Commission Steamer *Albatross*, described 12 sp. n. and four new subspecies from off Alaska, mainly the Aleutian Islands. Herein, five of those species and 1 subspecies are synonymized, and three of his subspecies are raised to species status. Fisher was a careful observer, with a good eye for variation, but he did not have access to scanning electron microscopy and a much larger collection than his is now available, and thus we have made some changes to his taxonomy. Most of these specimens are deposited at the NMNH, and a representative set exists at the CAS.

Although no specimens were reported from the Aleutians, Naumov (1960) reported many of Fisher’s species from the Kurile Islands and the Sea of Okhotsk; these have not been verified. Eguchi (1968) described *Allopora abei* Eguchi, 1968, from an unknown location in the Aleutian Islands, this species presumed to be a junior synonym of *Stylaster brochi* (Fisher, 1938).

Twenty years ago, Cairns (1991) described the deep-water, lamellate sp. n. and genus *Cyclohelia lamellata* from off Pribilof Islands. Cairns and Macintrye (1992) used seven Alaskan species for calcium carbonate polymorph determinations, some of which have been re-identified herein. Heifetz (2002) and Lindner et al. (2008) used molecular sequences of three genes (nuclear rDNA 18S, Calmodulin, and mitochondrial rDNA 16S) of 11 Alaskan stylasterid species in a phylogenetic study of the family.

## Material and methods

**Abbreviations:**

AB Auke Bay Marine Lab (NOAA), Juneau, Alaska


AL Alberto Lindner


Alb USFWS Albatross


CAS California Academy of Sciences, San Francisco


coll. private collector


FOMC Fiber Optic Monitoring Cruise of 2008


GM Geologisk Museum ved København Universitet, Copenhagen, Denmark


H:D Height to Diameter ratio of a gastrostyle


indet. gender indeterminate


MCZ Museum of Comparative Zoology at Harvard, Cambridge


MF Miller Freeman


NMNH National Museum of Natural History, Smithsonian Institution, Washington, DC


NOAA United States National Oceanic and Atmospheric Administration


RACE Resource Assessment and Conservation Engineering


USNM United States National Museum (now the NMNH)


WoRMS World Register of Marine Species


Specimens were obtained from collections deposited at the following institutions: CAS; MCZ; GM; and the NMNH. Specimens were also collected by one of us (AL) during NOAA’s RACE bottom trawling survey off the Gulf of Alaska and the Aleutian Islands in 2001 and 2002. Additional specimens were provided by Mr. Arthur Schultz and by NOAA’s Alaska Fisheries Science Center. We examined type specimens of all species reported herein.

Specimens were studied using scanning electron microscopy using the methodology as described by Cairns (1983). Genomic DNA and 37 new mitochondrial rDNA 16S sequences for the six *Errinopora* species from Alaska (GenBank accession numbers: JN572403-JN572439) were obtained as described in Cairns (2011), and the term pseudocyclosystem is defined as a cyclosystem-like structure (composed of a gastropore surrounded by dactylopores) that may be found at basal branches of some stylasterid species, and in which the dactylopores are usually not fused with the central gastropore and may not have the slits (dactylotome) of the dactylopore spines directed towards the central gastropore. In contrast to cyclosystemate stylasterids (e.g., *Stylaster*, *Conopora*, *Crypthelia*), pseudocyclosystemate species (e.g., *Errinopora*) have this polyp arrangement at basal portions of branches and colonies, and a different polyp arrangement at branch tips.

The specimens on which this study was based resulted from collections from both the United States government and university vessels as well as from privately owned fishing vessels, the latter often having many with colourful names. The US research vessels owned by the US Government are the *Albatross* and the *Miller Freeman*, whereas fishing vessels temporarily chartered by the US Government (for NOAA’s RACE surveys) included: *Dominator*, *Sea Storm*, *Pacific Knight*, and the *Vesteraalen*. Additional private fishing vessels included: *Alaska Beauty*, *Alaska Sea*, *AlaskaTrojan*, *Alaskan Leader*, *Ballyhoo*, *Delta Alfa*, *North Pacific*, *Ocean Olympic*, *Patricia Lee*, and *Shishaldin*. Additionaly, the manned submersible *Delta* and the Remotely Operated Vehicles *Jason II *and *Ropos* were also used to collect specimens as part of NOAA expeditions. The types of all species known from have been examined with the only exception of *Allopora abei*. All of these types are deposited at the NMNH and a subset at the CAS. Unless otherwise stated, the condition of specimens examined is assumed to be in the dry state.

## Taxonomy

**Family Stylasteridae Gray, 1847**

### Genus Distichopora

Lamarck, 1816

http://species-id.net/wiki/Distichopora

#### Diagnosis.

Colonies usually flabellate, with blunt branch tips. Coenosteum reticulate-granular and of many colors. Gastropores aligned along branch edge or, more rarely, meandering in lines on colony faces, the gastropore rows flanked (usually on both sides) by rows of horseshoe-shaped dactylopore spines. Gastro- and dactylopore tubes axial, extending along branch axis. Dactylostyles absent. Gastrostyles elongate (needle-shaped), often stabilized by transverse tabulae; a diffuse ring palisade sometimes present. Ampullae usually superficial and clustered.

#### Discussion.

Equally diverse in tropical and temperate waters as well as in shallow and deep environments, *Distichopora* is known from 26 Recent species and two fossil species (see Appeltans et al. 2001: WoRMS data base: www.marinespecies.org). The genus was revised by Boschma (1959) and Cairns (1983b).

#### Type species.

*Millepora violacea* Pallas, 1766, by monotypy.

#### Distribution.

Eocene to Recent: cosmopolitan except for off Antarctica, 1-806 m.

### Distichopora borealis

Fisher, 1938

http://species-id.net/wiki/Distichopora_borealis

Figs 1A–C 2A–K

Distichopora borealis Fisher, 1938: 543-545, pl. 70, fig. 3, pl. 71-73.—Broch 1942: 20.—Boschma 1957: 41 (listed).—Naumov 1960: 562, text-fig. 407.—Wing and Barnard 2004: 10, 27, fig. 26.—Heifetz et al. 2005: 133, 137 (listed).—Jamieson et al. 2007: 224 (listed).—Brooke and Stone 2007: 529, fig. 2I.—Stone and Shotwell 2007: 107 (listed).—Lindner et al. 2008: 3, and supplemental Table S1: 3 (phylogeny and DNA sequences).
Distichopora sp. Heifetz, 2002: 22 (listed).—Heifetz et al. 2005: 133 (listed).

#### Type material.

**Figure 1.**
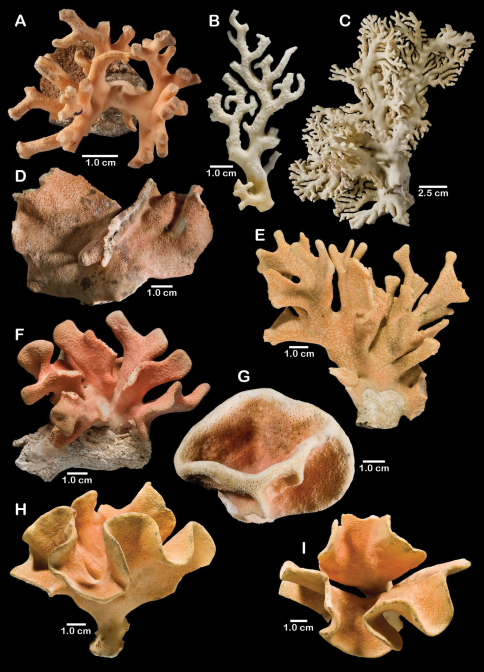
Color figures of skeleton of various Alaskan stylasterids: **A–C** *Distichopora borealis* **D–I** *Cyclohelia lamellata* **A** arborescent colony, USNM 1122878 **B** holotype, USNM 43274 **C** largest known colony, USNM 1122542 **D** holotype, USNM 85077 **E** digitate form, USNM 1122492 **F** intermediate form between lamellar and digitate, USNM 96236 **G** juvenile corallum **H–I** side and apical views of fully developed lamellar colony.

Holotype, *Alb*-3480, a female colony (dry) of 9 cm length (USNM 43274, Fig. 1B). Paratypes, *Alb*-3480, include 3 male, 4 female, 1 indeterminate dry colonies, and SEM stubs 1489–1494 (USNM 76810) and 2 dry female colonies from *Alb*-4781 (USNM 76375).

#### Type locality.

*Alb*-3480: 52°06'00"N, 171°45'00"W (Amukta Pass, Aleutian Islands), 518 m.

#### Material examined.

*Alaskan Leader* 40, 52°09'18"N, 175°40'42"W, 174 m, 8 Jun 2002, 1 female and 1 male, USNM 1122542; *Alaskan Leader* 54–14, 51°44.4'N, 178°16.7'W, 567–680 m, 11 Jun 2002, 6 indet., AB02–29; *Alaska Trojan*, 51°24'47"N, 178°50'02"W, 640 m, 2 Feb 2000, 1 female, USNM1122447; *Ballyhoo*, 54°49'52"N, 178°43'14"E, 236, 7 June 2000, 1 female, USNM 1122565; *Delta* 5600, 52°33'47"N, 179°26'45"W, 225 m, 15 Jul 2002, 1 male, USNM 1122543; *Dominator* 971–181, 51°27'43"N, 178°35'20"E, 384 m, 27 Jul 1997, 1 female and 1 male, USNM 1123357; *Dominator* 971–219, 53°00'03"N, 172°18.86'E, 223 m, 4 Aug 1997, 1 female, 2 male, USNM 1123358; *Jason2*–2103–7-4, 51°47.898'N, 179°57.169'E, 1267 m, 1 female, AB08–0036; *MF* 70, 52°03'24"N, 179°25'06"E, 174 m, 1 male, USNM 77047; *Ocean Olympic*, 52°04.78'N, 177°13.39'E, 256 m, 1 male, AB00–57; *Ocean Olympic*, 52°08.67'N, 176°35.46'E, 292 m, 1 female, AB00–21; *Ommaney* 8, 56°11'42"N, 135°06'31"W, 53 m, 17 Aug 2006, 1 female in alcohol, USNM 1086324; *Patricia Lee*, 51°53.44'N, 179°47.7'E, 298 m, 1 indet., AB00–41; *Sea Storm* 36, 53°05'45"N, 171°41'56"E, 458 m, 19 Jun 2002, 1 female and 1 male in alcohol, USNM 1076507 and 1122770; *Sea Strom* 92, 51°33'34"N, 177°36'59"W, 367 m, 4 Jul 2002, 7 males, USNM 1137601, 1123541, 1122874–76; *Sea Storm* 93, 51°50'59"N, 178°26'02"E, 390 m, 5 Jul 2002, 2 male, USNM 1122877 and 1123287; *Sea Storm* 105, 52°08'59"N, 176°02'17"E, 201 m, 8 Jul 2002, 2 female in alcohol, USNM 1122884; *Sea Storm* 106, 52°10'46"N, 175°10'34"E, 165 m, 8 Jul 2002, 1 female and 1 male in alcohol, USNM 1122885 and 1123288; *Sea Storm* 107, 52°10'28"N, 175°14'14"E, 214 m, 8 Jul 2002,4 female and 2 males in alcohol, USNM 1122866–70; *Sea Storm* 108, 52°11'32"N, 175°17'E, 208 m, 8 Jul 2002, 1 female and 3 males, USNM 1122862–65; *Sea Storm* 109, 52°17'16"N, 175°20'56"E, 238 m, 8 Jul 2002, 6 female, 8 male, 3 indet. in alcohol, USNM 1122886 and 1123289; *Sea Storm* 122, 52°02'49"N, 175°16'54"E, 143 m, 13 Jul 2002, 2 female and 7 males, USNM 1122858–61; *Sea Storm* 132, 52°12'02"N, 176°05'56"E, 150 m, 15 Jul 2002, 1 female in alcohol, USNM 1122854; *Sea Storm* 133, 52°13'40"N, 176°02'13"E, 148 m, 15 Jul 2002, 1 male, 2 females in alcohol, USNM 1122855–57; *Sea Storm* 149, 52°30'15"N, 173°33'03"W, 239 m, 21 Jul 2002, 1 male in alcohol, USNM 1122883; *Sea Storm* 151, 52°33'40"N, 173°33'03"W, 203 m, 21 Jul 2002, 1 female and 2 male, USNM 1122878–80; *Sea Storm* 155, 52°38'43'N, 173°34'31"W, 393–401 m, 22 Jul 2002, 1 male and 1 indet., USNM 1122881–82; *Shishaldin*, 53°56'24"N, 179°49'31"E, 318 m, 14 Mar 2000, 1 female and 1 male, USNM 1122549; *Shishaldin*, 54°07'N, 179°45'E, 366 m, 20 Feb 2000, 1 female and 1 male, USNM 1122544–455; *Shishaldin*, 54°23'29"N, 179°19'46"E, 413 m, 12 Feb 2000, 1 male, USNM 1122548; *Vesteraalen* 941–165, 51°34'N, 178°19'E, 470 m, 18 Jul 1994, 1 female and 1 male, USNM 96247; Slear, coll., 51°59'52"N, 176°47'05"E, 241 m, 13 Nov 2000, 1 male, USNM 1122458; University of Washington, 51°32'N, 179°15'W, 278–289 m, 1 Sep 1968, 1 female, USNM 76377; 54°20'15"N, 133°03'19"W, 457–466 m, 1 Sep 2002, 1 female, USNM 1123535; 51°13'43"N, 179°49'31"E, 465–529 m, 13 Jun 2000, 1 male, USNM 1137421.

#### Description.

**Figure 2.**
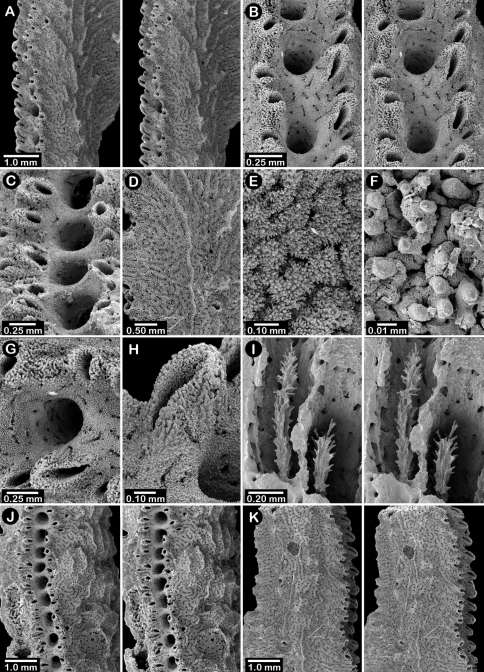
Paratypes of *Distichopora borealis*, USNM 76810: **A** stereo view of carinate branch face **B–C** pore row, B being a stereo view **D** branch face with longitudinal ridges **E–F** coenosteal texture **G** gastropore **H** dactylopore spine **I** stereo view of two gastrostyles **J** stereo view of pore row and female ampullae **K** stereo view of branch face and male ampullae.

Colonies usually uniplanar (Fig. 1B), but occasionally multiplanar or arborescent (Fig. 1A, C). Largest specimen examined (USNM 1122542, Fig. 1C) 11 cm tall and 20 cm broad, having a basal diameter of 3 cm, colonies broader than tall not uncommon. Base of colony broadly encrusting. Branching irregularly dichotomous, occasionally anastomotic. Large basal branches circular to elliptical in cross section, but terminal branches have flattened faces, thus rectangular in cross section. Branch tips blunt and rounded. Coenosteum white to light orange, the latter usually having branches with a white core. Coenosteum reticulate-granular in texture, the strips being 0.10-0.14 mm in width, each strip covered with tall (22 µm tall, 9 µm in diameter), slender granules arranged 8-10 across the width of a strip. Faces of distal branches often bear longitudinal ridges (Fig. 2A, D, K), some ridges up to 4 mm in length, sometimes ending distally in what appears to be a dactylopore opening (Fig. 2D).

Pore rows well defined (Fig. 2B), 1.0–1.3 mm in width, and restricted to lateral branch edges, although isolated rows and some irregularities may occur proximally on large colonies. Gastropores linearly arranged in a shallow sulcus, closely spaced (approximately 12–16 per cm), and circular in shape, measuring 0.33–0.41 mm in diameter. Gastropore tubes long and slightly curved, a diffuse ring palisade present near gastrostyle tip, the elements globular and about 30 µm in diameter; tabulae absent. Gastrostyles long, slender, and fragile, up to 1 mm in length and 0.09 mm in diameter, having high H:W ratios up to 8-10. Gastrostyles longitudinally ridged and bear numerous long slender spines up to 0.07 mm in length and 10–12 µm in diameter. U-shaped dactylopore spines arranged on both sides of gastropore row, but often more common on one side than the other, e.g., 18-20 dactylopore spines on one side opposite 28-40 spines on other side. Dactylopore spines up to 0.4 mm tall and 0.25-0.30 mm in width, with a dactylotome width of 0.07-0.08 mm, always directed toward or slightly obliquely to the adjacent gastropore. Dactylostyles absent.

Female ampullae (Fig. 2J) prominent, superficial, hemispherical mounds clustered on branch faces, 1.0–1.5 mm in diameter. Ampullae usually covered by low ridges, either radiating from the central apex or arranged in parallel, longitudinal rows aligned with branch axis. Lateral efferent pores 0.25–0.30 mm in diameter. Male ampullae (Fig. 2K) also superficial mounds, but mostly embedded in coenosteum (internal), also occurring in clusters on branch faces. Male ampullae irregular in shape, often with an apical tip, and smaller than female ampullae (0.35–0.45 mm in diameter).

#### Remarks.

Among the 26 Recent species of *Distichopora* (see Cairns et al. 1999; Cairns 2005; and Appeltans et al. 2011: WoRMS database – www.marinespecies.org), the most similar is *Distichopora borealis japonica* Broch, 1942, described from Sagami Bay, Japan (110 m). Although similar in gross morphology, subspecies *japonica* differs in coenosteal texture (porcellaneous), and in ampulla shape, each female ampulla having more than one efferent pore and the male ampullae clustered densely in a continuous mat. Also, the dactylotomes of the dactylopore spines are oriented upward to outward (away from the gastropores), not inward toward the gastropores. These differences would argue for an elevation to the species level of this putative subspecies. The observations are based on examination of a fragment of a syntype of *Distichopora borealis japonica* (USNM 76868) and additional Japanese material deposited at the NMNH (USNM 44198, 44199, 44202, and 44217). Of the 111 specimens examined, 45 are female, 53 male, and 13 indeterminate in gender.

#### Distribution.

Common in the Aleutian Islands from the Near Islands to Amukta Pass, including Bowers Bank; off Cape Ommaney, Alexander Archipelago, and Dixon Entrance, Queen Charlotte Islands; 53–1267 m, the shallowest record being from Cape Ommaney.

### Genus Cyclohelia

Cairns, 1991

http://species-id.net/wiki/Cyclohelia

#### Diagnosis.

Colonies lamellar, often attaining a large size. Coenosteum reticulate-granular, reddish-orange. Gastro- and dactylopores uniformly distributed on corallum faces, of the axial type. Dactylopore spines circular to elliptical, enclosed by a thin wall for entire perimeter. Dactylostyles absent. Gastrostyles elongate but tabulae absent; a diffuse ring palisade present. Ampullae internal.

#### Discussion.

The genus is monotypic. According to the molecular analysis of Cairns 2005), rendering the colonies of *Distichopora anceps* as a “transition” in morphology between the two genera.

#### Type Species.

*Cyclohelia lamellata* Cairns, 1991, by original designation.

#### Distribution.

Known only from the Aleutian Islands and Pribilof Islands, 27–567 m.

### Cyclohelia lamellata

Cairns, 1991

http://species-id.net/wiki/Cyclohelia_lamellata

Figs 1D–I 3A–C

Cyclohelia lamellata Cairns, 1991: 384-386, figs 1a-g, 2a-g.—Heifetz 2002: 22 (listed).—Wing and Barnard 2004: 10, 27, fig. 25, not Appendix fig. 1 (=*Errinopora undulata*).—Stone and Shotwell 2007: 107 (listed).—Brooke and Stone 2007: 529-530, figs 2K, 3E.—Jameison et al. 2007: 224 (listed).—Lindner et al. 2008: 3, and supplemental Table S1: 3 (phylogeny and DNA sequences).
Cyclohelia lamellate .—Heifetz et al. 2005: 133, 137 (listed).

#### Type material.

The holotype, a dry female colony (USNM 85077, Fig. 1D). **Type locality.** Off Pribilof Islands, 550 m.

#### Material examined.

*Alaskan Leader* 35, 53°01'48"N, 170°06'12"W, 172–178 m, 4 Jun 2002, 1 female, 1 male, USNM 1122491; *Alaskan Leader*, 54–14, 51°44.4'N, 178°16.7'W, 567–680 m, 11 Jun 2002, 1 female, AB02–0029; *Alaska Beauty*, 51°42.43'N, 176°56.05'E, 351 m, 3 Feb 2000, 1 female, AB00–15; *Delta* 5999–8E-5, 52°21.042'N, 179°30.483'W, 115 m, 4 Jul 2003, 3 fragments, AB10–0001; *Delta* 6000–10D-2, 51°50.8545'N, 179°49.6488'E, 150 m, 5 Jul 2003, 1 indet., AB; *Delta* 6001–10C-1, 51°52.396'N, 179°46.191'E, 230 m, 5 Jul 2003, 1 female, AB; *Delta* 6213–12B-1 , 2 and 4, 6 males, AB; *Delta*, 51°50'55"N, 179°48'35"W, 125 m, 17 Jul 2002, 1 female in alcohol (digitate form), USNM 1122494; *North Pacific*, 52°04.28'N, 176°05.35'E, 366 m, 1 indet., AB00–28; *Ocean Olympic*, 52°11.89'N, 176°17.3'E, 366 m, 20 Oct 2000, 1 female (digitate form), USNM 1122513; *Pacific Knight* 941–121, 51°38'00"N, 178°19'00"W, 0–373 m, 5 Jul 1994, 1 female and 1 male, USNM 96235 and 1122900; *Pacific Knight* 941–179, 52°00'N, 176°44'E, 0–90 m, 23 Jul 1994, 1 female (intermediate form), USNM 96236; *Patricia Lee*, 51°18'42"N, 179°30'04"E, 329 m, 20 Oct 2000, 1 male, USNM 1122488; *Patricia Lee* 51°53.44'N, 179°47.7'E, 298 m, 3 male, SEM stub 1488, AB00–41; *Sea Storm* 107, 52°10'28"N, 175°14'14"E, 214 m, 8 Jul 2002, 1 female in alcohol, USNM 1123297; *Sea Storm* 108, 52°11'32'N, 175°17'E, 208 m, 8 Jul 2002, 10 female, 6 male, 8 indet. (digitate form) in alcohol and dry, USNM 1122951, -54, -55, -57, -58, -59, 61, 1123290, 91; *Sea Storm* 111, 52°16'20"N, 175°59'13"E, 137 m, 9 Jul 2002, 1 male in alcohol, USNM 1122949; *Sea Storm* 112, 52°15'12"N, 176°10'42"E, 137 m, 9 Jul 2002, 1 indet., USNM 1122946; *Sea Storm* 116, 52°04'10"N, 177°14'25"E, 87–94 m, 11 Jul 2002, 1 male in alcohol (digitate form), USNM 1122942; *Sea Storm* 122, 52°02'49"N, 179°25'18"E, 143 m, 13 Jul 2002, 8 female, 4 male, 5 indet., USNM 1122899, 1122948, 1123294–96, -98; *Sea Storm* 123, 52°10'53"N, 179°37'02"E, 124 m, 13 Jul 2002, 2 male, USNM 1122952; *Sea Storm* 125, 52°12'53"N, 179°56'49"W, 89–96 m, 13 Jul 2002, 1 male, USNM 112305; *Vesteraalen* 3, 52°38'13"N, 169°47'14"W, 75–83 m, 22 May 2001, 1 male in alcohol, USNM 1076490; *Vesteraalen* 941–36, 52°56'N, 169°31'W, 0–227 m, 10 Jun 1994, 1 female, USNM 96237; *Vesteraalen* 941–46, 53°04'N, 170°09'W, 0–174 m, 12 Jun 1994, 1 female, USNM 96238; *Vesteraalen* 941–153, 52°10'N, 179°43'E, 0–94 m, 11 Jul 1994, 1 male, USNM 96239; “Vessel" 35, 51°45'48"N, 178°09'47"W, 200–400m, 4 Jun 2000, 2 female and 1 male, USNM 1122493; Renfro, coll., 51°56'35"N, 179°17'58"E, 236 m, 1 female, USNM 1122456; coll. Slear, 51°59'52"N, 176°47'05"E, 241 m, 13 Nov 2000, 1 male (digitate form), USNM 1122457; Petrel Bank, 1 male, 1 indet., USNM 1123540; 51°52.11'N, 179°49.51'W, 27 m, 17 Jul 2002, 1 female (digitate form), USNM 1122492.

#### Remarks.

This species is not redescribed herein as it was adequately described originally (Cairns 1991) and, being the only species in the genus, conforms to the genus diagnosis above. But, the species was previously known from only two specimens, the type and a specimen sequenced by Lindner et al. (2008), and thus more can be added to its description, including details of the male ampullae, based on the 81 additional specimens reported herein.

Colonies are usually firmly attached to a hard substrate by a robust cylindrical base and stem (Fig. 1H) up to 2.5 cm in diameter, which, at the height of about 2 cm, bifurcates into two lamellae or sheets, each sheet increasing its surface area by folding and undulating its surfaces into a complex three dimensional structure. Large colonies may attain a height of about 8 cm and a width of 10 cm, colonies wider than tall being the norm. However, a subset of colonies from seven stations (see Material Examined), termed the “digitate form”, differ from the typical colony morphology in having dissected plates, resulting in numerous flattened lobes and clavate branches (Fig. 1E). Furthermore, one colony (Fig. 1F) is intermediate between these two extremes, suggesting that these shapes are simply variations of the same species.

**Figure 3.**
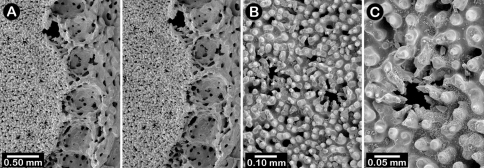
*Cyclohelia lamellata*, USNM 1122488: **A** stereo view of male ampullae in cross section **B–C** male efferent pores.

Male ampullae (Fig. 3A) entirely internal, and elliptical in shape, the greater axis of the ellipse perpendicular to the branch surface, unlike that of the female ampullae. Male ampullae are up to 0.6 mm in greater diameter and 0.45 mm in lesser diameter, occurring in great concentrations and invariably present on almost any broken surface. Each ampulla communicates to the surface by a circular efferent duct, the duct about 0.07–0.09 mm in diameter, which is surrounded by small inward projecting granules, giving the pore a star-shaped appearance (Fig. 3B–C). Of the 81 specimens examined, 34 are female, 29 male, and 18 indeterminate in gender.

#### Distribution.

In addition to the type locality off the Pribilof Islands, this species is common throughout the Aleutian Islands from the Rat Islands to the Islands of Four Mountains at 27–567 m, although it was more commonly collected from the western part of this range at depths of 100–300 m. The digitate form is known only from the western Aleutian Islands from 27–366 m.

### Genus Errinopora

Fisher, 1931

http://species-id.net/wiki/Errinopora

Errinopora Fisher, 1931: 397; 1938: 536.—Boschma 1956: F102; 1957: 57.—Cairns 1983a: 123; 1983b: 462. —Lindner 2005: 79-88.
Protoerrina Broch, 1935: 59; 1936: 99-100.

#### Diagnosis.

(emended from Lindner 2005): Colonies uniplanar to slightly bushy; branches round, elliptical, or lamellar in cross section, often robust with blunt tips. Coenosteal texture reticulate-spinose (with wide slits resulting in a spongy texture) or reticulate-granular; exterior surface of dactylopore spines usually inconspicuously longitudinally ridged; coenosteum orange, pink, and white. One species, *Errinopora cestoporina*, bears numerous perforated mounds on surface. Dactylopores dimorphic, the most common, termed the primary dactylopore spine, is U-shaped and usually robust (thick-walled), occurring randomly, in pseudocyclosystems, or often laterally fusing to form rows or taller terraces that flank rows of gastropores. When dactylopore spines flank both sides of a gastropore row and their dactylotomes are directed toward the gastropores it is termed bilateral or distichoporine; if only one row of spines flank a row of gastropores, then unilateral. If isolated, dactylotomes usually abcauline in orientation. Much smaller flush dactylopores, termed secondary dactylopores, which lack dactylostyles, commonly scattered over coenosteum of many species. Dactylostyles usually well developed, easily seen from external view. Secondary dactylopores much smaller, flush with coenosteum, and lack styles. Gastropores also dimorphic, the primary gastropores being circular in outline, flush with coenosteum (having no lip), and arranged in irregular vertical rows, short horizontal rows, or randomly. Tabulae and ring palisades absent. Gastrostyles lanceolate, covered with longitudinal or oblique, spiny ridges. Smaller secondary gastropores much smaller, having only a small gastrostyle or none at all. Female ampullae superficial hemispheres, often without an obvious efferent pore. Male ampullae usually smaller hemispheres and spongy.

#### Discussion.

The ten species in this genus are differentiated and compared in both a dichotomous key (see below) and tabular key (Table 1); six of them occur exclusively in the Aleutian Islands. Another species was tentatively assigned to this genus by Cairns (1983b), *Errinopora lobata* (Nielsen, 1919), a Paleocene fossil from Denmark. This species was re-examined by Bernecker and Weidlich (1990, 2005), based on subsequently collected non-type material from the Faske Formation in Denmark. They noted that whereas the dactylopore spines were typical of *Errina* or *Errinopora*, the spines did not contain dactylostyles, and thus resembled *Errina* more than *Errinopora*. We have examined the holotype of *Labiopora lobata* Nielsen, 1919 (GM1782), which is a uniplanar colony 10.2 cm tall and 8.0 cm wide, with branches about 0.5 cm in diameter embedded within a *Dendrophyllia* matrix. The colony has pores, or broken bulges, of three sizes: small and elongate (75–110 µm in width), medium and round to somewhat quadratic (0.3–0.53 mm wide), and large, round or somewhat triangular (up to 1.7 mm). The former are quite shallow and probably the result of a reticulate coenosteal surface, whereas the medium-sized pores are deep and possibly represent the gastropores, one of which has a cyclosystem-like structure 1.9 mm in diameter. The largest pores appear to be ruptured ampullae. None of them, however, resemble dactylopore spines like those reported by Nielsen (1919) or Bernecker and Weidlich (1990, 2005), suggesting that the species reported by the latter authors is neither *Errina* nor *Errinopora*. Likewise, the lack of dactylopore spines in the type of *Labiopora lobata* precludes it from being *Errinopora*, and thus we currently suggest an *incertae sedis* placement of this species until further analysis.

Species within *Errinopora* are unique with the Stylasteridae in having both dimorphic gastro- and dactylopores, a condition first noted by Fisher (1938) for three Alaskan species but interpreted exclusively as secondary dactylopores. Careful examination of longitudinal sections of several of these pores reveal that they contain not dactylostyles, but rather small gastrostyles. This implies that most species of *Errinopora* (all but *Errinopora fisheri* and *Errinopora cestoporina* Cairns, 1983, see Table 1) have two types of gastrozooids (feeding polyps), a unique case for stylasterids but previously reported for the hydractiniids *Stylactaria conchicola* (see Namikawa et al. 1992). Secondary gastropores differ from primary gastropores in being narrower and deeper, and in having only a minute or no gastrostyle. All but one species (*Errinopora fisheri*, see Table 1) also have dimorphic dactylopores: a large type surrounded by a prominent horseshoe-shaped spine, and smaller, flush pores only 40–110 µm in diameter, which do not have dactylostyles and are termed secondary dactylopores.

In comparison to other stylasterid genera, *Errinopora* is most similar to *Gyropora* Boschma, 1960, whose only species, *Gyropora africana* Boschma, 1960, has dactylopore spines and gastropores coordinated in pore rows, as in some species of *Errinopora*. *Errinopora* is also similar to *Errina* Gray, 1835 and *Errinopsis* Broch, 1935, whose species may also have thick-walled dactylopore spines. None of these genera, however, include species with dactylostyles, as in *Errinopora*. Among genera with species having dactylostyles, only species of *Errinopora*, *Inferiolabiata* Broch, 1951, and *Paraerrina* Broch, 1942 lack a coordination of gastropores and dactylopores in well-developed cyclosystems (whereas species of *Stenohelia* Kent, 1870, *Stylantheca* Fisher, 1931, *Stylaster* Gray, 1831 and *Calyptopora* Boschma, 1968 do have both dactylostyles and well-developed cyclosystems). *Inferiolabiata* differs from *Errinopora* in many characters, in particular by having thin-walled dactylopore spines (instead of thick-walled) that are markedly truncated at the tip (instead of rounded), whereas *Paraerrina* Broch, 1942 differs in having delicate dactylostyles (instead of robust) and either flush or only slightly raised dactylopore spines—instead of tall and robust (Cairns 1984, 1991). *Errinopora* is also one of the few stylasterid genera with species having calcitic, rather than aragonitic, colonies (Thompson and Chow 1955, Lowenstam 1964, Cairns and Macintrye 1992). The only other stylasterid genera with species known to have mostly calcitic colonies are *Errinopsis*, *Errina*, and one species of *Stylaster*—*Stylaster verrillii* (Dall, 1884) (see Cairns and Macintrye 1992). Within *Errinopora*, only *Errinopora cestoporina*, known solely from the Subantarctic Region, is known to have coralla formed by precipitation of aragonite. This result confirms the more general observation of the prevalence of calcitic stylasterids in the North Pacific (Cairns and Macintrye 1992), possibly related to the shallower depth of the Aragonite Saturation Horizon (ASH) in the Region (Guinotte et al. 2006).

In an attempt to investigate phylogenetic relationships, 37 partial mitochondrial rDNA 16S sequences were obtained for the six species of *Errinopora* from the Aleutian Islands, including the holotypes of *Errinopora dichotoma*, *Errinopora disticha*, *Errinopora fisheri* and *Errinopora undulata*. Based on Lindner et al. (2008) included *Errinopora nanneca* (specimen USNM1027820) and *Errinopora zarhyncha* Fisher, 1938 (specimen USNM1071915), and shows that these species diverged only about 4 million years ago. Moreover, the same study shows that *Cyclohelia lamellata* Cairns, 1991 and *Distichopora borealis* Fisher, 1938, two sympatric Alaskan stylasterids that are also clearly distinguishable with marked morphological differences (see above), may have diverged as recently as 1 million years ago. These results indicate that part of stylasterid species diversity in Alaska may have diverged only within the past 1-4 million years and, at least for *Errinopora*, the results presented herein show that despite the marked morphological differences, some species are not recovered as reciprocally monophyletic lineages (Baum and Shaw 1995, Avise 2000) using mitochondrial rDNA 16S.

#### Type species.

*Errina pourtalesii* Dall, 1884, by original designation.

#### Distribution.

North Pacific: Aleutian Islands, Kurile Islands, Sea of Okhotsk, Sea of Japan, off California. Subantarctic: off Tierra del Fuego, 40–658 m.

**Table 1.** Tabular Key of the Ten Species of *Errinopora* (pscs = pseudocyclsosystems)

	**i*Errinopora fisher***	**a*Errinopora cestoporin***	**a*Errinopora porifer***	**a*Errinopora undulat***	**a*Errinopora distich***	**i*Errinopora pourtalesi***	**a*Errinopora stylifer***	**a*Errinopora zarhynch***	**a*Errinopora nannec***	**a*Errinopora dichotom***
**Dactylopore Spines**										
Arrangement	Tall terraces and pscs	Tall terraces and pscs	Isolated or short unilateral rows	Pscs and short unilateral rows	Distichoporine rows; pscs at base	Distichoporine rows, unilateral rows, isolated, pscs	Distichoporine rows, pscs at base	Short unilateral rows, isolated	Short unilateral rows, pscs, isolated	Short unilateral rows, isolated
Compound Dactylopore Spines	Absent	Absent	Absent	Absent	Present	Present	Absent	Present	Absent	Present
Maximum Height	1.5 mm	1.3 mm	0.46 mm	0.25 mm	0.5–0.9 mm	1.1–1.2 mm	0.5–0.9 mm	to 3 mm	0.4 mm	1.2 mm
Wall Thickness	Not individualized	Thick	Thin	Thin	Thick	Thick	Thick	Thick	Thick	Thick
Dactylotome Shape	Elliptical pores	Slit	Apical pores	Slit	Slit	Slit	Slit	Slit	Slit	Slit
External Surface of Dactylopore Spine	Inconspicuously ridged	Reticulate-Granular	Not ridged	Inconspicuosly ridged	Inconspicuously ridged	Inconspicuously ridged	Inconspicuously ridged	Inconspicuously ridged	Prominently ridged	Inconspicuously ridged
Dactylostyle	Narrow	Robust	Moderate width	Robust	Robust	Robust	Robust	Narrow	Moderate width	Robust
Secondary Dactylopores	Absent	Present	Present	Present	Present	Present	Present	Present	Present	Present
**Corallum**										
Shape	Uniplanar	Bushy	Uniplanar	Lamellate	Uniplanar	Bushy	Uniplanar	Uniplanar	Uniplanar branching or plate-like	Three dimensional
Branch Size	Delicate	Delicate	Delicate	Sheets thin	Robust	Medium	Medium	Large	Delicate	Robust
Branch Cross Section	Circular to elliptical	Circular	Circular to slightly elliptical	Lamellar	Flattened	Circular	Circular to slightly elliptical	Circular to elliptical	Circular to lamellar	Circular to slightly elliptical
Color	Orange	White	White, light orange	Orange	Light orange	Orange	Orange, yellow	Orange	Orange, pink	Orange
**Other Characters**										
Gastropore Diameter	0.3–0.5 mm	0.30–0.55 mm	0.23–0.27 mm	0.30–0.45 mm	0.3–0.7 mm	0.20–0.38 mm	0.4 mm	0.2–1.1 mm	about 0.2 mm	0.3–0.5 mm
Secondary Gastropores	Absent	Absent	Present	Present	Present	Present	Present	Present	Present	Present
Coenosteal Texture	Reticulate-Spinose	Reticulate-Granular	Reticulate-Granular	Reticulate-Spinose	Reticulate-Spinose	Reticulate-Granular	Reticulate-Granular	Reticulate-Spinose	Reticulate-Spinose	Reticulate-Spinose
Unique Features	Gastropore tube constricted; ring palisade present; gastrostyles squat	Conical ampullae; coenenchymal papillae; gastrostyle pedicellate	Dactylopore spines conical		Gastrostyles flattened in cross section	Encrusting base		Gastropore tubes commodious		U-shaped branching axils
Distributon and Depth Range	Aleutian Islands, 455 m	Tierra del Fuego, 359–384 m	Okhotsk Sea, 190–250 m	Aleutian Islands, 350–640 m	Aleutian Islands, 178–536 m	Central California,49–183 m	Off Japan, Okhotsk Sea, 84–379 m	Aleutian Islands,207–658 m	Aleutian Islands,40–517 m	Aleutian Islands,178–217 m

#### Key to the Recent species of Errinopora (bold face = occurs off Alaska)

**Table:** 

1	Dactylopore spines arranged in pseudocyclosystems near base but otherwise predominantly laterally fused into tall terraces flanking the lower side (unilateral) of one or a series of gastropores, their dactylotomes facing the gastropores	2
1'	Dactylopore spines isolated, and/or arranged in pseudocyclosystems, short unilateral rows, or bilateral (distichoporine) rows	3
2	Individuality of fused dactylopore spines lost, the terraces having a continuous edge and the dactylotomes being elliptical pores; ampullae hemispherical; coenosteum orange; no coenosteal papillae; known only from Alaskan	*Errinopora fisheri* sp. n.
2'	Fused dactylopore spines retain individuality, terraces having an irregular edge and dactylotomes being traditionally slit like; ampullae conical; coenosteum white; perforated papillae common on coenosteum; known only from the Subantarctic	*Errinopora cestoporina* Cairns, 1983
3	Dactylopore spines predominantly conical, having a circular dactylotome	*Errinopora porifera* Naumov, 1960
3'	Dactylopore spines horseshoe-shaped, having a slit-like dactlylotome	4
4	Colonies lamellate (sheet-like)	5
4'	Colonies branching	6
5	Colony a continuous, sinuous, thin sheet; pseudocyclosystems predominant form of dactylopore spine arrangement; dactylopore spines very short (0.25 mm) and thin walled	*Errinopora undulata* sp. n.
5'	Colony a series of dissected blades of a larger sheet; pseudocyclosystems occur only at colony base, transverse rows of dactylopore spines being more common; dactylopore spines short (0.40 mm) and thick walled	*Errinopora nanneca* Fisher, 1938 (lamellate form)
6	When arranged in rows, dactylopore spines predominantly in bilateral (distichoporine) rows, the dactylotomes of both rows facing the flanked gastropore row (spines may also be isolated, in pseudocyclosystems, and/or arranged in unilateral rows)	7
6'	When arranged in rows, dactylopores spines predominantly in unilateral rows flanking a single row of gastropores, the dactylotomes of only the proximal row facing the gastropores (spines may also be isolated and/or arranged in pseudocyclosystems especially basally)	9
7	Coenosteum reticulate-spinose; branches and gastrostyles flattened; known only from the Aleutian Islands	*Errinopora disticha* sp. n.
7'	Coenosteum reticulate-granular; branches and gastrostyles circular in cross section; known from off California and off Japan and Russia	8
8	Colonies bushy, with an encrusting base; ampullae small (0.5-0.9 mm in diameter); compound dactylopore spines present; known only from off California	*Errinopora pourtalesii* (Dall, 1884)
8'	Colonies uniplanar without an encrusting base; ampullae large (up to 1.6 mm in diameter); compound dactylopore spines lacking; known only from off Japan and Russia	*Errinopora stylifera* (Broch, 1935)
9	Dactylopore spines tall (up to 3 mm), and often compound; dactylostyle narrow in respect to dactylotome; gastropore tube short and commodious, much larger than gastrostyle	*Errinopora zarhyncha* Fisher, 1938
9'	Dactylopore spines smaller (0.4-1.2 mm), rarely compound; dactylostyle moderate to large with respect to dactylotome; gastropore tube longer and less commodious, providing a close fit for the gastrostyle	10
10	Dactylopore spines short (up to 0.4 mm); gastropores small (0.2 mm in diameter); colonies uniplanar, with angled branch axils	*Errinopora nanneca* Fisher, 1938 (branched form)
10'	Dactylopore spines taller (up to 1.2 mm); gastropores larger (0.3-0.5 mm in diameter); colonies 3-dimensional, with U-shaped branch axils	*Errinopora dichotoma* sp. n.

**Figure 4.**
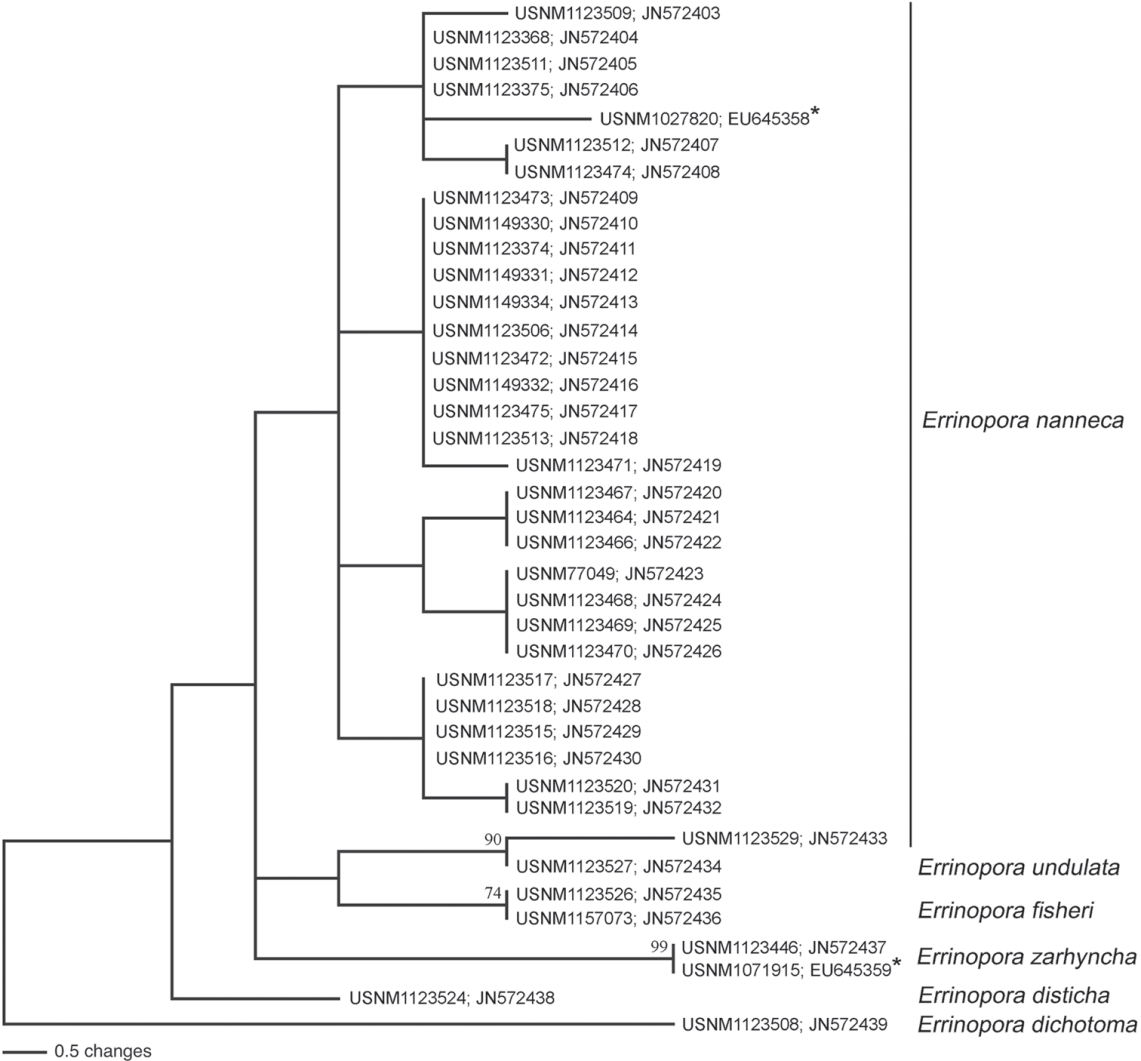
Midpoint rooting phylogeny of the Alaskan species of *Errinopora* estimated using mitochondrial rDNA 16S sequences. Numbers at nodes are the result of 100 bootstrap pseudoreplicates whenever >70. Shown is one of six most parsimonious trees (length: 38; CI: 0.868). Numbers preceded by ‘USNM’ represent specimen catalog numbers at the United States National Museum, Smithsonian Institution. Numbers preceded by ‘EU’ or ‘JN’ represent GenBank accession numbers. Asterisks (*) indicate the two specimens and rDNA 16S sequences of *Errinopora nanneca* and *Errinopora zarhyncha* previously published by Lindner et al. (2008).

### Errinopora fisheri

Lindner & Cairns sp. n.

urn:lsid:zoobank.org:act:9D4515E9-8067-4E6B-8C59-46B775683599

http://species-id.net/wiki/Errinopora fisheri

Figs 5A 6A–K

Errinopora fisheri Lindner, 2005: 100–102, figs 4.6A, 4.14 (unpublished name).

#### Type material.

**Figure 5.**
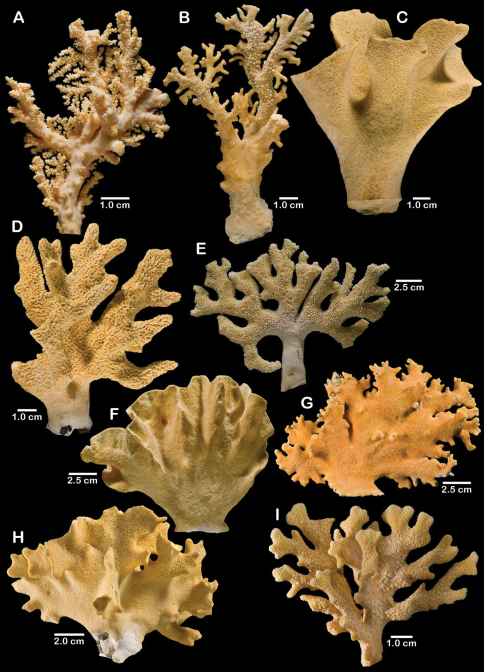
Color figures of skeleton of various Alaskan stylasterids: **A** *Errinopora fisheri* **B, G–I** *Errinopora nanneca* **C, F** *Errinopora undulata*, **D** *Errinopora disticha* **E** *Errinopora zarhyncha*. **A** holotype, USNM 1123526 **B** holotype, USNM 42875 **C** holotype, USNM 112327 **D** holotype, USNM 1123524 **E** holotype, USNM 42874 **F** lateral view of a large paratype, USNM 1123528 **G** lamellate colony with digitate distal branches, USNM 1123462 **H** lamellate colony, USNM 44070 **I** intermediate form between lamellate and digitate, USNM 1123510.

Holotype: *Pacific Knight* 204, a dry female colony, USNM 1123526 (Fig. 5A). Paratype: *Sea Storm* 36, 53°05'45"N, 171°41'56"E, 458 m, 1 branch, USNM 1157073. **Type locality.** 53°06'N, 171°42'E (off Attu Island, Aleutian Islands), 455 m, 31 Jul 1994.

#### Etymology.

Named in honor of Walter K. Fisher, who wrote the original revision of the Aleutian Island stylasterids (Fisher 1938) and described the genus *Errinopora* (Fisher, 1931).

#### Material examined.

Holotype.

#### Description.

Holotype (Fig. 5A) uniplanar with a secondary flabellum at mid-height, 9 cm in height and 6.5 cm in width, with a basal branch diameter of 10 × 7.5 mm. Branches circular to slightly elliptical in cross section and irregularly dichotomous, small-diameter branches often diverging from larger main branches; branch anastomosis absent. Coenosteum reticulate-granular in texture, the strips being 80-90 µm in width and covered with small granules 5-15 µm in diameter; strips bordered by slits 40-45 µm wide. Coenosteum light orange.

**Figure 6.**
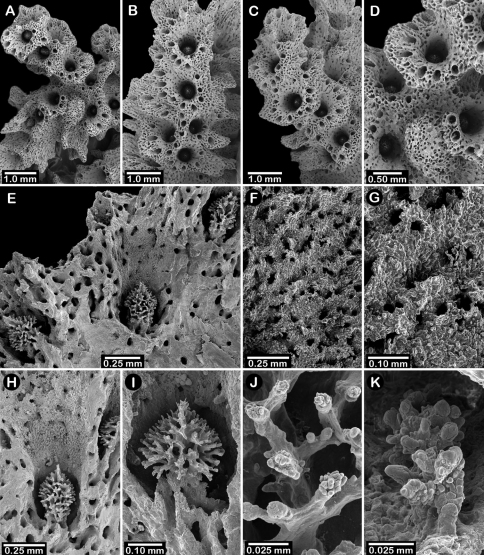
Holotype of *Errinopora fisheri*, USNM 1123526: **A–D** branch tips with dactylopore spines arranged in crescents (female ampulla shown in figure D) **E, H–I** lateral view of gastrostyles **F–G** coenosteal texture **J** detail of gastrostyle spines **K** dactylostyle.

Dactylopore spines favor one face of corallum over the other, arranged in abcauline, crescent-shaped terraces flanking one or two gastropores (Fig. 6A-D). Terraces sometimes almost completely surround a gastropore (Fig. 6C-D), resembling a cyclosystem of 1.5 mm diameter with an adcauline diastema, much as in *Stylaster*, but isolated dactylopore spines also fairly common, especially near base of colony. Dactylopore terraces quite thin (0.35 mm) and up to 1.5 mm in height, and, although laterally fused, the individuality of each dactylopore spine is subsumed into the more continuous terrace structure; compound dactylopore spines absent; exterior surface of spines longitudinally ridged. Terraces often horizontally oriented on branches, and slightly flared around gastropore(s). Dactylotomes not slit-shaped as in most other *Errinopora*, but instead small elliptical pores about 0.27 mm long and 0.15 mm in width occurring only near tips of spine structure; dactylotomes always directed toward a gastropore. Dactylostyles small, about 50 µm in width, having stubby elements up to 25 µm in length and 10-15 µm in diameter. Secondary dactylopores absent.

Gastropores circular, flush with coenosteum, 0.3-0.5 mm in diameter, being fairly consistent in size; gastropores isolated, not arranged in rows; secondary gastropores absent. Gastropore tube cylindrical, but with a slight medial constriction; often a ring palisade is present, the elements 50 µm tall and 15-25 µm in diameter. Gastrostyle squat, an illustrated one being 0.33 mm tall and 0.29 mm in diameter (Fig. 6I). Gastrostyles covered with longitudinal anastomosing ridges as consistent with the genus. Female ampullae (Fig. 6D) hemispherical but often irregular in shape due to protruding dactylopore spines. Female ampullae 1.0–1.2 mm in diameter; efferent pores not observed. Male ampullae unknown.

#### Remarks.

Even though represented by only one specimen, *Errinopora fisheri* can be distinguished from other Alaskan congeners by the distinctively terraced arrangement of its dactylopore spines, its small elliptically-shaped dactylotomes, its squat gastrostyles, and its constricted gastropore tube with a ring palisade (see Dichotomous Key and Table 1). Most of these features, however, are shared with *Errinopora cestoporina*, a species known only from Burdwood Bank and off Tierra del Fuego at 359-384 m. *Errinopora cestoporina* differs in having differently shaped ampullae, a different corallum color (white), small perforated mounds covering the coenosteum, and a differently shaped gastrostyle.

#### Distribution.

Known only from Aleutian Islands: off Attu Island; 455-458 m.

### Errinopora undulata

Lindner & Cairns sp. n.

urn:lsid:zoobank.org:act:CCDBFA1A-6AA3-4C95-A85A-216A3A760B4C

http://species-id.net/wiki/Errinopora_undulata

Figs 5C, F 7A–J

Errinopora undulata Lindner, 2005: 120-122, figs 4.6B, 4.19 (unpublished name).

#### Type material.

Holotype: Vessel and collector unknown, Amukta Pass, 350–500 m ,1 dry female colony 12 cm tall, USNM 1123527 (Fig. 5C). Paratypes: Vessel and collector unknown, Amukta Pass, 356–640 m, 1996, 1 dry female colony, USNM 1123528; south of Semisopochnoi Island, 366 m, 1 dry female colony, USNM 88371. **Type locality.** Aleutian Islands: Amukta Pass, 350–500 m.

#### Etymology.

The specific name *undulata* (from the Latin *undulatus*, meaning wavy) refers to the sinusoidal margin of the lamellate colonies.

#### Material examined.

Types.

#### Description.

Colonies lamellate, but wavy in construction resulting in a continuous, thin (1.7–2.0 mm thick), sinusoidal distal edge. Largest colony (USNM 1123528, Fig. 5F) 13 cm tall and 15 cm wide, with a massive basal branch 4.5 × 2.5 cm in diameter; holotype (Fig. 5C) smaller and less intact, measuring 12 cm tall and 11 cm wide, with a basal branch 3.5 × 2.8 cm in diameter. Parasitic spionid worm tubes found in only one colony (USNM 1123528). Coenosteum quite porous, consisting of a reticulum of narrow spiny strips separated by even wider slits, the spines being 10-15 µm in diameter. Coenosteum orange.

**Figure 7.**
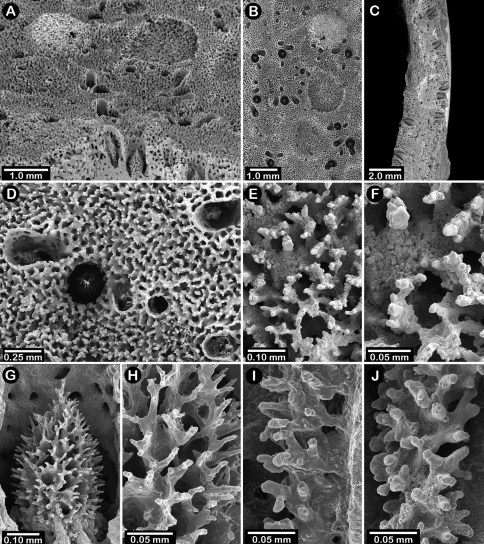
Female paratype of *Errinopora undulata*, USNM 1123528: **A–B, D** plate surface showing female ampullae, dactylopore spines, and gastropores **C** cross section of plate showing several gastrostyles **E–F** coenosteal texture **G–H** lateral view of a gastrostyle and detail of spination **I–J** lateral and apical view of a robust dactylostyle.

Dactylopore spines occur equally on both branch faces, but less abundant toward corallum base. Most dactylopores clustered around isolated gastropores in pseudocyclosystems, 3–5 spines surrounding one pore (Fig. 7B). Pseudocyclosystems most common toward base of colony but also occur frequently on more distal parts of corallum where they are interspersed with short, transverse rows of dactylopore spines that border the distal margins of one or more gastropores, their dactylotomes facing upward (abcauline) toward the gastropore; compound dactylopore spines absent. Dactylopore spines fairly short (rarely taller than 0.25 mm), 0.40–0.45 mm in width, and thin walled, the majority of width being the dactylotome; dactylopore spine walls longitudinally ridged. Dactylostyles robust (Fig. 7I–J), up to 0.12 mm in width, consisting of cylindrical elements up to 72 µm in length and 15 µm in diameter. Secondary flush dactylopores common, about 75 µm in diameter.

Gastropores circular, flush with coenosteum, and 0.3–0.45 mm in diameter; secondary gastropores about 0.19 mm in diameter. Gastropore tubes short and lack a ring palisade. Gastrostyles lanceolate, the figured style (Fig. 7G) 0.54 mm in height and 0.29 mm in diameter, covered with longitudinal anastomosing spiny ridges.

Female ampullae (Fig. 7A–B) irregularly-shaped, flattened hemispheres up to 1.6 mm in diameter. Efferent pores not observed. Male ampullae unknown.

#### Remark.

**.** *Errinopora undulata* is quite similar to the lamellate form of *Errinopora nanneca*, but differs in a number of points, one being its colony form, which is a continuous wavy sheet of corallum, whereas that of *Errinopora nanneca* is more like a series of smaller dissected flat blades of a larger plate. *Errinopora undulata* also has shorter (0.25 vs 0.4 mm) and thin-walled (vs thick-walled) dactylopore spines, and larger gastropores (0.45 mm vs 0.20 mm in diameter) (see Dichotomous Key and Table 1).Of the 247 known stylasterid species (Appeltans et al. 2011: WoRMS database: www.marinespecies.org) only five have adopted a lamellate corallum shape, four of these occurring in the Aleutian Islands (*Cyclohelia lamellata*, *Stylaster repandus*, *Errinopora nanneca*, and *Errinopora undulata*), the fourth being the Hawaiian *Distichopora anceps* Cairns, 1978.

#### Distribution.

Known only from the Aleutian Islands: Amukta Pass and south of Semisopochnoi Island; 350–640 m (unconfirmed).

### Errinopora disticha

Lindner & Cairns sp. n.

urn:lsid:zoobank.org:act:3CA47961-430E-4FDF-BB00-15C6DDB19829

http://species-id.net/wiki/Errinopora_disticha

Figs 5D 8A–J

Errinopora disticha Lindner, 2005: 97–99, figs 4.2A-B, 4.7B, 4.13 (unpublished name).

#### Type material.

Holotype: a dry female colony 10 cm in height, coll. Renfro, USNM 1123524 (Fig. 5D). Paratypes: also from type locality, 1 female, USNM 1123521; *Alaskan Leader* 35, 53°01'48"N, 170°06.62'W, 172–178 m, 4 Jun 2002, 2 male, USNM 1123525; *Alb-*3480, 52°06'N, 171°45'W, 518 m, 8 Jul 1893, 2 males in alcohol, USNM 43768; *Alb-*3480, see above, 1 male, USNM 1148212 (ex USNM 52247, paratype of *Errinopora zarhyncha*); 52°03'26"N, 179°12.34'E, 475 m, 27 Apr 2000, 1 male, USNM 1123523; 51°52'46"N, 179°17'24"E, 536 m, 16 Jul 2000, 2 female, USNM 1123522. **Type locality.** 51°53'12"N, 179°17.40'E (west of Semisopochnoi I., Petrel Bank), 530 m.

**Etymology.** The specific name *disticha* (from the Greek *distichos*, meaning “of two rows") refers to the short distichoporine pore rows of this species.

**Material examined.** Types.

**Description.** Colonies uniplanar to multiplanar, consisting of cylindrical to highly compressed branches, the greater axis of compressed branches oriented in plane of colony flabellum and reaching up to two times length of lesser axis. Branching dichotomous and non-anastomotic, terminating in blunt tips and forming U-shaped axils between branching. Holotype (Fig. 5D) 10 cm tall and equally wide, with a basal branch diameter of 22 × 20 mm; largest specimen (USNM 1123523) 13.5 cm in height. Parasitic spionid polychaete tubes found in only one corallum (USMM 1123525). Coenosteum typical for the genus: reticulate spinose, the narrow strips separated by broad porous slits, resulting in a very porous surface. Coenosteum light orange; branch cores white or lighter orange.

**Figure 8.**
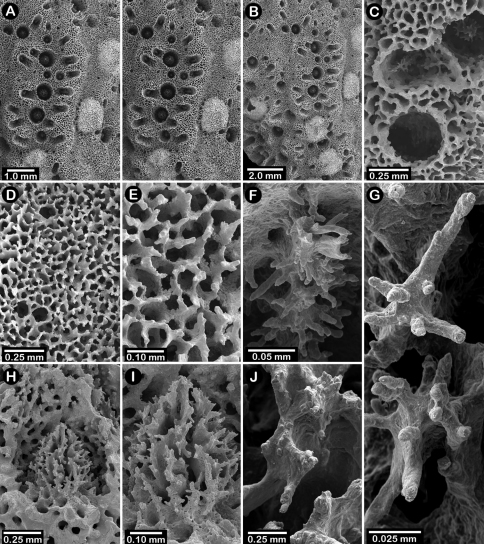
Holotype of *Errinopora disticha*, USNM 1123524: **A–B** distichoporine pore rows and female ampullae (A a stereo view) **C** gastropore and adjacent dactylopore spine with dactylostyle **D–E** coenosteal texture **F–G** dactylostyle **H–I** lateral view of gastrostyle **J** detail of gastrostyle spines.

Dactylopore spines mostly arranged in long meandering distichoporine (bilateral) rows of up to 18 laterally fused spines (Fig. 8A-B). Two rows or terraces of dactylopore spines usually flank each gastropore row, the dactylotomes facing the gastropore; however, the number of dactylopore spines is often unequal on either side of a pore row and are occasionally absent from one side. Toward corallum base, pore rows decrease in length, often resulting in a single gastropore surrounded by 3–6 dactylopore spines approximating a pseudocyclosystem. Dactylopore spines fairly short (0.5–0.9 mm in height) and thick-walled, the dactylotome consisting of one-third to half width of spine; compound dactylopore spines common. Dactylostyles robust, up to 75 µm in width, consisting of cylindrical elements up to 53 µm in length and 5–10 µm in diameter; exterior surface longitudinally ridged. Secondary dactylopores, which lack styles, very common on coenosteum, especially back sides of dactylopore spines, these pores being circular, flush with the surface, and measure about 75–110 µm in diameter.

Gastropores circular, flush with coenosteum, and variable in size, ranging from 0.3–0.7 mm in diameter, both size classes often adjacent to one another. Gastropores usually closely spaced and unilinearly arranged in a shallow sulcus created by adjacent dactylopore spine terraces. Gastropore tubes cylindrical, fairly shallow, and lack a ring palisade; secondary gastropores 0.19–0.30 mm in diameter. Gastrostyles lanceolate but somewhat stout, the figured symmetrical style (Fig. 8H–I) being 0.49 mm in height and 0.36 mm in diameter, however, many gastrostyles are asymmetrical, being elliptical to flattened in cross section. Gastrostyles bear longitudinal, spiny, anastomosing ridges, and fill most of gastropore cavity.

Female ampullae (Fig. 8A–B) hemispherical to somewhat flattened (1.3–1.5 mm in diameter), their basal perimeter often slightly undercut, and often with an irregular or ridged surface. Discrete efferent pores never observed. Male ampullae small, porous, hemispherical blisters 0.4–0.6 mm in diameter, often occurring in great concentrations on coenosteum.

#### Remarks.

*Errinopora disticha* is the only Alaskan *Errinopora* species to have its dactylopore spines arranged bilaterally in distichoporine rows flanking a row of gastropores, however two other species have a distichoporine arrangement: *Errinopora pourtalesii* (California) and *Errinopora stylifera* (Japan to Okhotsk Sea) (see Dichotomous Key and Table 1). *Errinopora disticha* differs from those two species in having reticulate-spinose coenosteum (vs reticulate-granular), and flattened gastrostyles and branches. Of the 10 specimens examined, 4 are female and 6 male.

#### Distribution.

Known only from the Aleutian Islands: off Petrel Bank, Amukta Pass, and off Four Kings Islands; 178-536 m.

### Errinopora zarhyncha

Fisher, 1938

http://species-id.net/wiki/Errinopora_zarhyncha

Figs 5E 9A–L

Errinopora zarhyncha Fisher, 1938: 539-541, pl. 68, pl. 69, fig. 1.—Boschma 1957: 58.—Thomson and Chow 1955: 23, 30 (mineralogy).—Not Naumov 1960: 559.—Lowenstam 1964: 377 (mineralogy).—Lowe 1967: 82.—Cairns 1983b: 462.—Cairns and Macintrye 1992: 100-101 (mineralogy).—Cairns et al. 1999: 43 (listed).—Heifetz 2002: 22 (listed).—Wing and Barnard 2004:27, 57, fig. 27.—Lindner 2005: 123-127, fig. 4.2D, 4.10, 4.20 (redescription, key).—Heifetz et al. 2005: 133, 137 (listed).—Stone and Shotwell 2007: 108 (listed).—Jamieson et al. 2007: 224 (listed).—Lindner et al. 2008: 3, and supplemental Table S1: 3 (phylogeny and DNA sequences).
Errinopora zarhincha : Lowenstam 1964: 382-383 (misspelling).

#### Type material.

Holotype, *Alb*-3480, a dry male colony 14 cm in height, USNM 42874 (Fig. 5E). Paratypes: *Alb*-3480, 3 dry female branches, USNM 52247; *Alb*-3480, 1 branch, CAS 28186. **Type locality.** *Alb*-3480: 52°06'N, 171°45'W (Amukta Pass, Aleutian Islands), 518 m.

#### Material examined.

*Shishaldin*, 53°56'24'N, 179°49'31"E, 318 m, 14 May 2000, 1 male, USNM 1123447; *Shishaldin*, 54°54'05"N, 178°37.27'E, 410 m, 11 Feb 2000, 3 female branches, and SEM stubs 1542–43, USNM 1123448; *Vesteraalen* 941–59, 52°02'00"N, 172°12'W, 658 m, 14 Jun 1994, 3 branches of a female colony, both dry and in alcohol, USNM 96234; *Vesteraalen* 941–62, 51°58'N, 172°40'W, 207 m, 15 Jun 1994, 1 female, USNM 96233; *Vesteraalen*, 51°13'17"N, 179°05'57"E, 488 m, 4 Jun 2000, 1 male, USNM 1071915; 51°13'43"N, 179°49'31"E, 465–529 m, 13 Jun 2000, large male colony, USNM 1123446; Types.

#### Description.

**Figure 9.**
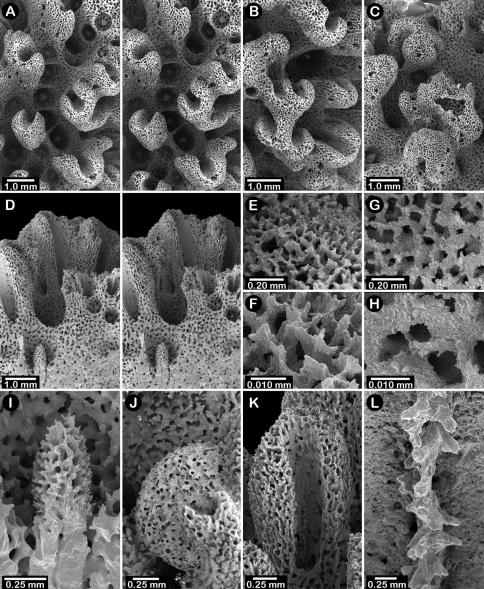
*Errinopora zarhyncha*, **A–I, K–L** male from USNM 1071915, **J** female from USNM 1123448: **A–C** branch surface showing tall dactylopore spines, gastropores, and ruptured male ampullae in figure C **D** stereo view of gastro– and dactylostyles and dactylopore spines **E–H** coenosteal texture **I** lateral view of a gastrostyle **J** female ampulla **K** dactylotome of a dactylopore spine **L** dactylostyle.

Colonies uniplanar or multiplanar, robust, with fairly close dichotomous branching, leaving little space between branches; branch anastomosis occurs but uncommon. Largest colony 26 cm in height and 27 cm in width, with a massive basal branch 4.3 cm in diameter (USNM 1123446). Branches circular to slightly flattened in cross section, attenuating to thick (7–15 mm in diameter), blunt tips. Parasitic spionid polychaete worms form tubes along branch axes in two colonies (USNM 96233, 96234), the tubes Fig. 8 in cross section. Coenosteum quite porous in texture (Fig. 9E–H), consisting of a reticulum of thin (25 µm), spinose ridges, separated by wide slits or series of pores. This texture also present on ampullae and dactylopore spines, in the latter the coenosteal strips forming longitudinal ridges. Coenosteum orange.

Dactylopore spines occur on all branch surfaces and are quite variable in orientation, sometimes forming rows of 10–13 spines laterally fused into a short tier on one side of a pore row (unilaterally), the dactylotomes usually facing upward (abcauline), but dactylopore spines also oriented with their dactylotomes facing downward (adcauline) or laterally, and occasionally are isolated; compound dactylopore spines common. Dactylopore spines quite large and thick-walled, up to 3 mm in height and 1.5 mm in width, the dactylotome occupying one-fourth to one-third width of spine; exterior surface longitudinally ridged. Small, circular, flush secondary dactylopores, measuring only 0.08–0.11 mm in diameter, occur on walls of dactylopore spines, often several on each spine. Whereas dactylopore spines are quite large, dactylostyles are small and thin, only about 30–35 um in width, bearing short spines up to 23 µm in length and 14 µm in diameter.

Gastropores circular and quite variable in size, up to 1.1 mm in diameter, often arranged linearly in valleys created by adjacent rows of dactylopores. Large gastropores often sit directly adjacent to much smaller ones; secondary gastropores about 0.38 mm in diameter. Gastropore fairly shallow, lacking a ring palisade, affording an easy view of base of pore, as the gastrostyle occupies only a small part of gastropore cavity. Gastrostyles lanceolate and slender, up to 0.9 mm in height, and bear longitudinal anastomosing ridges, the gastrostyle size commensurate with diameter of gastropore tube.

Female ampullae inconspicuous (Fig. 9J) and not common, rarely seen in cross section branch fracture. Female ampullae hemispherical, often overshadowed by tall dactylopore spines or becoming incorporated into dactylopore spines. Efferent pores elusive; when detectable they are lateral in position, and about 0.7 mm in diameter, but more often a spent female ampulla lacks its upper half or is simply a crater in the coenosteum, a function of its thin, porous coenosteal cover. Male ampullae equally inconspicuous (Fig. 9C), roughly hemispherical, highly porous, and only about 0.6–0.7 mm in diameter.

#### Remarks.

*Errinopora zarhyncha* is one of three species in the genus having a predominantly unilateral arrangement of dactylopore spines in which only one row of laterally fused spines (usually the proximal row) have their dactylotomes facing a gastropore or gastropore row, the other species being *Errinopora nanneca* and *Errinopora dichotoma* (see Dichotomous Key and Table 1). *Errinopora zarhyncha* differs from the other two species in having very tall dactylopore spines, relatively small dactylostyles, large robust colonies, and gastropore tubes that are much larger than their gastrostyles (see Dichotomous Key and Table 1). Naumov (1960) reported this species from the Kurile Islands, but based on his description in which he reports gastropore diameters of only 0.20-0.25 mm and dactylopore spines only 0.6 mm in height, we conclude that he is referring to a different, and as yet unknown species. Of the 8 lots of specimens examined, 4 are female, and 4 male. Coralla were determined to be calcitic by Thompson and Chow (1955) and Cairns and Macintrye (1992).

#### Distribution.

Endemic to Aleutian Islands in a somewhat disjunct distribution: Amchitka Pass, Bowers Bank, and off Seguam Island; 207-658 m.

### Errinopora nanneca

Fisher, 1938

http://species-id.net/wiki/Errinopora_nanneca

Figs 5B,G–I 10A–K

Errinopora nanneca Fisher, 1938: 538–539, pl. 66, fig. 1, pl. 67, pl. 69, figs 2, 2a.—Boschma 1957: 57.—Naumov 1960: 559, fig. 404.—Cairns 1983b : 462.—Cairns et al. 1999: 43 (listed).—Cairns and Macintrye 1992: 100-101 (mineralogy).—Heifetz 2002: 22 (listed).—Lindner 2005: 87 (redescription, key), 103-106, figs 4.2E, 4.8, 4.15.—Heifetz et al. 2005: 133, 137 (listed).—Wing and Barnard 2005: 27 (listed).—Brooke and Stone 2007: 528, figs 2G, 3C.—Stone and Shotwell 2007: 107 (listed).—Jamieson et al. 2007: 224 (listed).—Lindner et al. 2008: 3, and supplemental Table S1: 3 (phylogeny and DNA sequences).
Errinopora nannacea : Cairns 1983a: 127 (incorrect spelling).
Errinopora nummeca : Lowe 1967: 83 (incorrect spelling).
Errinopora pourtalesi : Wing and Barnard 2004: 58, fig. 28 (USNM 1123450).

#### Type material.

Holotype: *Alb*-3599, a dry female colony 13 cm in height, USNM 42875 (Fig. 5B). Paratypes: *Alb*-3599, 6 colonies, including that figured by Fisher (1938: plate 67), 4 female, 1 male, 1 indet., all dry, USNM 52263. All other specimens mentioned by Fisher from *Alb*-3599 and 4777 are expressed excluded from type status according to ICZN (1999) article 72.4.6. **Type locality.** *Alb*-3599: 52°05'N, 177°40'E (off Kiska Island, Rat Islands, Aleutian Islands), 101 m, 9 Jun 1894.

**Material examined.** *Alb*-3480, 52°06'N, 171°45'W, 517 m, 8 Jul 1891, 1 indet., part of type series of *Errinopora zarhyncha*, ex. USNM 52247; *Alb*-3599, type locality, 8 female, 8 male, USNM 52248; *Alb*-4777, 52°11'N, 179°49'E, 95 m, 5 Jun 1906, 11 female, 7 male, USNM 44070, 52249, 60299, and 62715; *Alaskan Leader* 35, 53°01'48"N, 170°06'12"W, 172–178 m, 4 Jun 2002, 1 female, 1 male, USNM 1123451 and 1123532; *Alaskan Leader* 54, 51°45'48"N, 179°06'08"E, 90–467 m, 11 Jun 2002, 1 female, USNM 1123378; *Delta*, 51°13'30"N, 179°53'17" E, 40 m, 16 Jul 2002, 1 indet. in alcohol, USNM 1123377; *Delta* 5599, 52°33.47'N, 179°26.44'E, 225 m, 15 Jul 2002, 1 male, USNM 1123452; *Delta* 5605, 51°50'55"N, 179°48'13"W, 125 m, 17 Jul 2002, 1 male, 4 indet., USNM 1123450 and 1123462; *Delta* 5607, 51°23'57"N, 179°48'35"W, 182 m, 16 Jul 2002, 1 female, USNM 1123459; *Delta* 5620, 51°57'40"N, 176°00'42"E, 150 m, 24 Jul 2002, 2 female with fragments in alcohol, USNM 1027820 and 1123510; *Delta* 5622, 51°57.5'N, 176°50.1'W, 140 m, 24 Jul 2002, 2 female, 1 male, and fragments in alcohol, USNM 1123468, 1123472, and 1123472; *Delta* 5625, 51°57.7'N, 176°50.2'W, 130 m, 25 Jul 2002, 1 female, USNM 1123473; *MF* 833–47, 51°55'36"N, 176°52'48"W, 201 m, 5 Aug 1983, 1 female and 1 male, USNM 77049; *MF* 833–49, 52°00.2'N, 176°21.4'W, 115 m, 5 Aug 1983, 1 male in alcohol, USNM 77031; *MF* 833–56, 52°02'06"N, 176°22'36"W, 249 m, 6 Aug 1983, 1 female, USNM 77044; *MF* 833–69, 52°02.8'N, 179°27.2'E, 63 m, 25 Aug 1983, 1 indet., in alcohol, USNM 77030; *Pacific Knight* 941–61, 52°17'N, 173°06'W, 0–143 m, 17 Jun 1994, 1 female, USNM 96254; *Sea Storm* 86, 51°36'21"N, 176°17'39"W, 345 m, 3 Jul 2002, 1 female in alcohol, USNM 1123475; *Sea Storm* 92, 51°33'34"N, 177°40'W, 367 m, 4 Jul 2002, 3 female, 3 male, USNM 1123529, 1123469–70, and 1123376; *Sea Storm* 100, 51°42'59"N, 175°47'07"E, 86 m, 7 Jul 2002, 1 indet., in alcohol, USNM 1123520; *Sea Storm* 105, 52°08'59"N, 175°06'47"E, 201 m, 8 Jul 2002, 1 indet. in alcohol, USNM 1123519; *Sea Storm* 108, 52°11'32"N, 175°17'E, 208 m, 8 Jul 2002, 1 male in alcohol, USNM 1123517; *Sea Storm* 111, 52°16'20"N, 175°59'13"E, 137 m, 9 Jul 2002, 1 indet. in alcohol, USNM 1123518; *Sea Storm* 112, 52°15'12"N, 176°00'42"E, 137 m, 9 Jul 2002, 1 indet. in alcohol, USNM 1123512; *Sea Storm* 114, 52°02'29"N, 177°39'E, 130 m, 10 Jul 2002, 1 male, USNM 1123515; *Sea Storm* 115, 52°04'16"N, 177°19'04"E, 165 m, 10 Jul 2002, 2 indet. in alcohol, USNM 1123474 and 1123513; *Sea Storm* 116, 52°04'10"N, 177°14.4'E, 87–94 m, 11 Jul 2002, 5 male, 1 indet., USNM 1123516; *Sea Storm* 118, 52°00'14"N, 177°19'04"E, 104–111 m, 11 Jul 2002, 2 female, 2 male, USNM 1123375 and 1123471; *Sea Storm* 122, 52°02'49"N, 179°25'18"E, 143 m, 13 Jul 2002, 1 indet., USNM 1123511; *Sea Storm* 126, 52°13'22"N, 179°49'26"W, 115 m, 13 Jul 2002, 1 female in alcohol, USNM 1123466; *Sea Storm* 128, 52°03'22"N, 179°48.13'W, 242 m, 14 Jul 2002, 1 female in alcohol, USNM 1123374; *Sea Storm* 157, 52°12'35"N, 172°12'20"W, 348 m, 23 Jul 2002, 1 female, USNM 1123467; *Vesteraalen* 941–50, 52°34'N, 170°40'W, 0–88 m, 13 Jun 1994, 3 female, 3 male, USNM 96252 and 1138188; *Vesteraalen* 941–59, 52°12'N, 172°12'W, 0–360 m, 14 Jun 1994, 3 male, USNM 96251 and 1123514; *Vesteraalen* 941–61, 52°05'N, 172°26'W, 0–156 m, 15 Jun 1994, 5 female, USNM 96253; *Vesteraalen* 941–151, 52°11'N, 179°44'E, 0–151 m, 10 Jul 1994, 1 indet., USNM 96257; *Vesteraalen* 941–153, 52°10'N, 179°43'E, 0–94 m, 10 Jul 1994, 1 female, 4 indet., USNM 96531; *Vesteraalen* 941–154, 52°04'N, 179°47'E, 0–254 m, 11 Jul 1994, 1 male, USNM 96258; *Vesteraalen* 941–163, 51°37'N, 178°25'E, 0–155 m, 18 Jul 1994, 1 female, 1 male, USNM 96256; *Vesteraalen* 941–185, 52°04'N, 176°30'E, 0–91 m, 24 Jul 1994, 1 male, USNM 96255;*Vesteraalen* 3, 52°37'48"N, 169°47'16"W, 80 m, 21 May 2001, 1 female in alcohol, USNM 1123368; Gulf of Alaska, 219 m, 1 indet., USNM 77414; Renfro, coll., 52°02'17"N, 179°25'21"E, 236 m, 5 Apr 2000, 1 female, USNM 1123455; 52°18'N, 179°48'35"W, 490 m, 28 May 2000, 1 indet., USNM 1123457.

#### Description.

Colonies quite variable in shape. Most colonies examined uniplanar, consisting of irregularly dichotomous, non-anastomotic branching (e.g., the holotype, Fig. 5B). The opposite extreme is multilobate and multiplanar colonies (Fig. 5H), composed of thin blades of corallum set in a three dimensional arrangement. Virtually all intergrades in colony shape were observed, including some having both large flattened lobes and slender branches (Fig. 5G). Tallest colony examined (USNM 96252) 21 cm in height with a basal branch diameter of 3.5 cm, but a broken colony having a basal diameter of 4 cm (USNM 1123516) implies an even larger size. Although distal branches often circular in cross section, more often they are somewhat compressed in branching plane. Parasitic spionid polychaete worms often form tubes along axis of branches, Fig. 8 in cross section. Coenosteum reticulate-spinose, the narrow strips only about 60 µm wide and bordered by slits of equal width, each strip bearing irregularly unilinearly arranged spines, altogether producing a porous or rough coenosteal texture. Coenosteum light orange to light pink.

**Figure 10.**
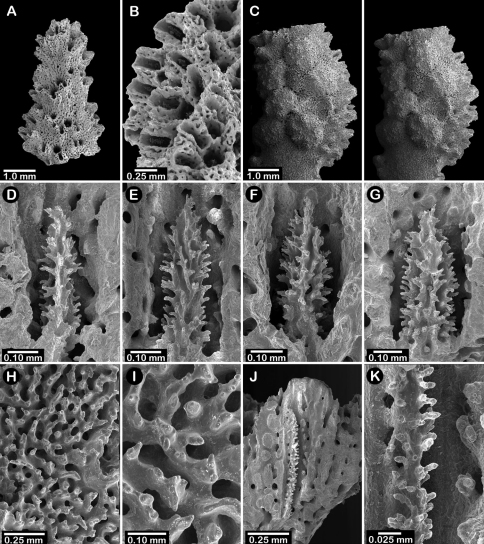
*Errinopora nanneca*, **A–C** female holotype, **D–K** USNM 96252: **A–B** dactylopore spines on branch tips **C** stereo view of clustered female ampullae **D–G** lateral views of gastrostyles **H–I** coenosteal texture **J–K** dactylostyle.

Dactylopore spines isolated or arranged in transverse to oblique rows on distal branches, their dactylotomes facing upward (abcauline), their edges often fusing with edges of adjacent dactylopore spines. On more proximal branches and the basal branch, dactylopore spines fewer in number, and arranged in pseudocyclosystems, short rows, isolated, or as circles around small islands of 2–5 gastropores. Dactylopore spines strongly favor one face of corallum, and are much less common on opposite face. Dactylopore spines relatively small, only about 0.4 mm in maximum height and 0.30-0.35 mm in width, the dactylotome occupying middle third. Small (40–115 µm in diameter) secondary dactylopores flush with coenosteum common. Outer surface of dactylopore spines prominently ridged; inner surface bears a moderately robust dactylostyle (40–50 µm in width, Fig. 10J–K) composed of elements up to 18 µm tall and 7 µm in diameter.

Gastropores circular and flush with coenosteum, 0.15-0.44 mm in diameter, the average about 0.20 mm. Gastropore tube cylindrical, without a ring palisade. Gastrostyles lanceolate, up to 0.55 mm in height, bearing spinose longitudinal, sometimes anastomosing, ridges that themselves bear small spines up to 32 µm long and 8 µm in diameter. Smaller secondary gastropores, lacking gastrostyles, also present, these 0.11–0.19 mm in diameter.

Female ampullae (Fig. 10C) large hemispheres 1.1–1.8 mm in diameter, occurring fairly densely and equally on both corallum faces. Dactylopore spines often occur on female ampullae. Efferent pores rarely observed, but are lateral and up to 0.5 mm in diameter. Male ampullae smaller mounds 0.4–0.7 mm in diameter, clustered, and somewhat irregular in shape. Both types of ampullae porous, like the coenosteum.

#### Remarks.

*Errinopora nanneca* is one of three species in the genus having a predominantly unilateral arrangement of dactylopore spines in which only one row of laterally fused spines (usually proximal to gastropores) have their dactylotomes facing a gastropore or gastropore row, the other species being *Errinopora zarhyncha* and *Errinopora dichotoma* (see Dichotomous Key and Table 1). *Errinopora nanneca* is distinguished from *Errinopora zarhyncha* in the account of the latter species, but differs from *Errinopora dichotoma* in having shorter dactylopore spines and smaller gastropores, uniplanar colonies, and prominently ridged dactylopore spines. Of the 126 specimens examined, 58 are female, 42 male, and 26 indeterminate in gender. The corallum was found to be 100% calcitic according to Cairns and Macintrye (1992).

#### Distribution.

Aleutian Islands from eastern Rat Islands to Islands of Four Mountains, including Petrel Bank; 40-517 m.

### Errinopora dichotoma

Lindner & Cairns sp. n.

urn:lsid:zoobank.org:act:7200AE40-A33D-4D86-A96C-1B6FC259A159

http://species-id.net/wiki/Errinopora_dichotoma

Figs 11D 12A–M

Errinopora dichotoma Lindner, 2005: 93-96, figs 4.2H, 4.7A, 4.12 (unpublished name).

#### Type material.

**Figure 11.**
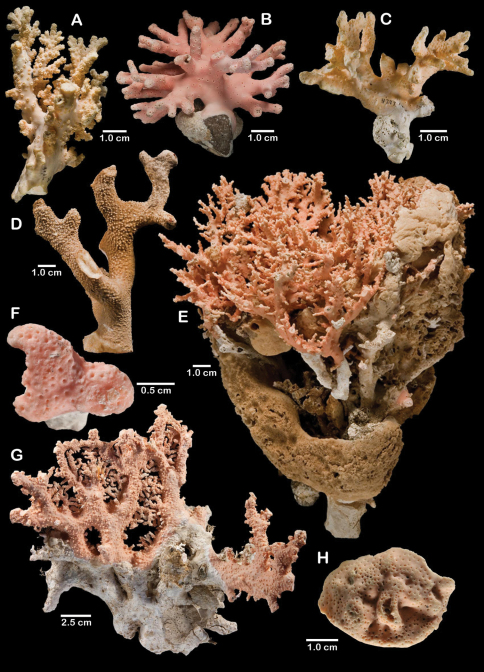
Color figures of skeleton of various Alaskan stylasterids: **A, E, G** *Stylaster brochi* **B, F, H** *Stylaster verrillii* **C** *Stylaster stejnegeri*, **D** *Errinopora dichotoma*. **A** holotype, USNM 43264 **B** arborescent colony, USNM 1027819 **C** holotype, USNM 43271 **D** holotype, USNM 1123508 **E** large delicate sponge encrusted colony, USNM 1123304 **F** holotype of *Allopora moseleyi*, USNM 6851 **G** massive colony, USNM 1123006 **H** syntype, USNM 4193.

Holotype: *Dominator* 971–73, 1 alcohol-preserved male colony 11.5 cm in height, USNM 1123508 (Fig. 11D). Paratypes: *Alaskan Leader*, 53°01'42"N, 170°05.99'W, 200–400 m, 4 Jun 2000, 1 male, USNM 1123507; *Alaskan Leader* 35, 53°01'48"N, 170°06'12"W, 172–178 m, 4 Jun 2002, 1 female, 1 male, USNM 1137600; *Dominator* 971–73, topotypic, 1 dry branch, male, USNM 1148143. **Type locality.** *Dominator* 971–73, 52°33'10"N, 179°25'18"E (off northwestern Petrel Bank), 217 m, 26 Jun 1997.

#### Etymology.

The specific name *dichotoma*, meaning “in two parts" (from the Greek *dicha*, meaning “in two" and *tomos*, meaning “part" or “slice"), referring to the dichotomous branching mode of this species.

#### Material examined.

Types.

#### Description.

**Figure 12.**
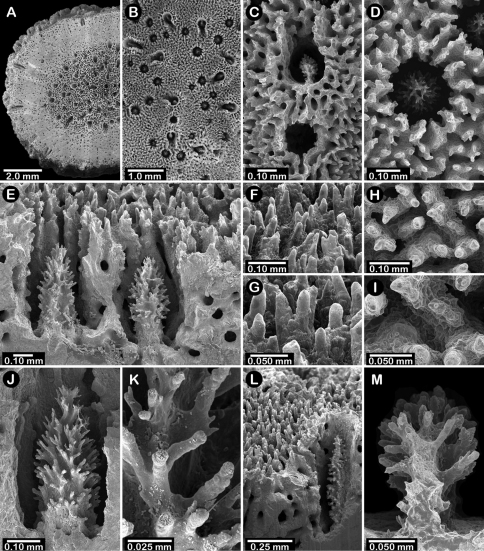
Holotype of *Errinopora dichotoma*, USNM 1123508: **A** branch cross section **B** colony surface showing gastro– and dactylopores **C** dactylopore with dactylostyle (upper) and secondary gastropore (lower) **D** gastropore **E, J** gastrostyles **F–I** coenosteal texture **K** gastrostyle spines **L–M** dactylostyle.

Colonies three dimensional, robust, and sparsely branched, branching equally and widely dichotomously, resulting in broad U-shaped axils; branch anastomosis absent. Largest colony (holotype, Fig. 11D) 11.5 cm in height, with a basal branch diameter of 16 × 20 mm. Branches circular to elliptical in cross section, attenuating to thick (7–8 mm in diameter), blunt tips; tips of most branches missing (broken) from type material. As in most species of *Errinopora*, coenosteum quite porous (Fig. 12F–I), composed of a reticulum of thin, spinose strips separated by wide slits or series of pores, the spines being up to 55 µm in height and 15–24 µm in diameter. Coenosteum orange, branch cores being white.

Dactylopore spines occur on all branch surfaces and quite variable in orientation (Fig. 12B), sometimes forming transverse or longitudinal rows of up to 7 laterally fused spines, spines sharing the same laterally fused row sometimes oriented in opposite directions, their dactylotomes facing in opposite directions. Isolated dactylopore spines also present. Dactylopore spines of moderate size and thick-walled, up to 1.2 mm in height and 1.1 mm in width, the dactylotome occupying one-fourth to one-third width of spine. Small (80 µm diameter), circular secondary dactylopores common. Dactylostyles robust (Fig. 12C, L–M), each about 0.125 mm in width, composed of elements up to 40 µm in height and 10-12 µm in diameter.

Gastropores circular, sometimes arranged in rows, otherwise randomly arranged, 0.3-0.5 mm in diameter; pores lack a ring palisade. Secondary gastropores (Fig. 12C, lower) 0.22–0.30 mm in diameter. Gastrostyle occupies most of gastropore cavity. Gastrostyles lanceolate, up to 0.5 mm in height, bearing longitudinal, anastomosing, spinose ridges.

Intact female ampullae never observed, but coenosteal depressions 1.0–1.1 mm in diameter are common in one specimen, these presumed to be spent female ampullae. Male ampullae small, porous hemispheres 0.5–0.6 mm in diameter.

#### Remarks.

*Errinopora dichotoma* is one of three species in the genus having a predominantly unilateral arrangement of dactylopore spines in which only one row of laterally fused spines (usually proximal to gastropores) have their dactylotomes facing a gastropore or gastropore row, the other species being *Errinopora nanneca* and *Errinopora zarhyncha*. *Errinopora dichotoma* is compared to those species in their respective accounts and in the Dichotomous Key and Table 1. Of the five specimens examined, 1 was presumed to be female and 4 male.

#### Distribution.

Endemic to Aleutian Islands: off Petrel Bank, off Islands of Four Mountains; 178–217 m (Cairns 1992; Appeltans et al. 2011).

### Genus Stylaster

Gray, 1831

http://species-id.net/wiki/Stylaster

#### Diagnosis.

Colonies branching in a flabellum or a bush shape (in only one case lamellate). Coenosteum usually reticulate-granular but may be linear-imbricate; coenosteum of many colors and hardness. Gastro- and dactylopores arranged in conventional cyclosystem arrangement, with only one gastrostyle per cyclosystem; pores of the peripheral type; supernumerary dactylopores often present. Cyclosystems arranged on branch edges and/or on corallum faces, or uniformly on all branch surfaces, but not unifacially. Gastropore tube single-chambered, but may be constricted by a ring palisade. Dactylostyles present. Ampullae usually superficial.

#### Discussion.

The genus and its various grouping are discussed by Cairns (1983b) and a key to all stylasterid genera, including the groups of *Stylaster*, is provided by Cairns (1992). Currently there are 80 recognized Recent species and 7 fossil species (Appeltans et al. 2011: WoRMS data base: www.marinespecies.org). It is by far the most species-rich and diverse genus of the stylasterids.

#### Type species.

*Madrepora roseus* Pallas, 1766, by subsequent designation (Milne Edwards and Haime 1850: xxii), a member of Group B.

#### Distribution.

Oligocene to Recent: cosmopolitan from depths of 0-2010 m (Cairns 1992; Appeltans et al. 2011).

### *Stylaster* (Group A)

**Diagnosis.** Species of *Stylaster* in which coralla form branching colonies, and in which cyclosystems are uniformly arranged on all sides of cylindrical, blunt branches.

**Discussion.** Previously called *Allopora*, these species were synonymized with *Stylaster* (see Cairns 1983b) because no definitive break could be established between species having uniformly arranged and sympodially arranged cyclosystems.

#### Stylaster brochi

(Fisher, 1938)

http://species-id.net/wiki/Stylaster_brochi

Figs 11A, E, G 13A–L

Allopora brochi Fisher, 1938: 517-518, pl. 42, figs 3-3d, pl. 44, pl. 45, fig 1.—?Naumov 1960: 569-570, text-fig. 413.— Boschma 1957: 19.
Allopora abei Eguchi 1968: 34, pl. 34, figs 1-3.—Mori 1980: 17 (listed).
Stylaster brochi : Cairns 1983b: 429.—Heifetz 2002: 22 (listed).—Wing and Barnard 2004: 11, 27.—Heifetz et al. 2005: 133, 137 (listed).—Stone and Shotwell 2007: 108 (listed).—Brooke and Stone 2007: 521, figs 2A, 3A.—Jameison et al. 2007: 224 (listed).
Stylaster abei .—Cairns 1983b: 430.
Stylaster polyorchis .—Lindner et al. 2008: 3, supplemental Table S1: 2.
Stylaster campylecus .—Lindner et al. 2008, 3, supplemental Table S1: 2.

##### Type material.

*Allopora brochi*: *Alb*-4777: The dried holotype is deposited at the USNM (43264, also SEM stub 1497), measuring 9 cm in height (Fig. 11A); a paratype is also deposited at the CAS (28183). **Type locality.** *Albatross* 4777: 52°11'N, 179°49'E (Petrel Bank, Aleutian Islands), 79–95 m.

*Allopora abei*: Holotype, dry, Dept. of Mineral Sciences, Tohoku University (1001). **Type locality.** Off the Aleutian Islands, exact locality and depth unknown.

##### Material examined.

Holotype, USNM 43264; *Alb*-3599, 52°05'N, 177°40'E, 101 m, 9 Jun 1894, 1 female, USNM 76814 (part of type lot of *Stylaster campylecus*); *Alb*-4779, 52°11'N, 179°57'W, 99–102 m, 5 Jun 1905, 1 female, USNM 44069; *Alaskan Leader* 35, 53°01'42"N, 170°05'59"W, 200–400 m, 4 Jun 2000, 1 male, USNM 1122516; *Delta* 5607, 51°23'02"N, 179°01'38"W, 126 m, 18 Jul 2002, 1 female, USNM 1122486; *Delta* 5620, 51°57'40"N, 176°50'01"W, 150 m, 24 Jul 2002, 5 female, 5 males, 5 indet., some in alcohol, USNM 1112304, 1123302, 1123304, 1123345–1123350; *Delta* 5622, 51°57'37"N, 176°49'58"W, 140 m, 24 Jun 2002, 2 indet., USNM 1123303; *Delta* 5624, 51°47'40"N, 176°49'58"W, 140 m, 25 Jun 2002, 1 male, USNM 1123300; *Delta* 5625, 51°57'42"N, 176°50'01"W, 156 m, 25 Jun 2002, 1 female, 1 indet., USNM 1027821; *Delta* 6000–10D-12, 51°50.828'N, 179°49.674'E, 112 m, 5 Jul 2003, 1 female, AB10–0002; *Dominator* 971–73, 52°33'10"N, 172°20'58"W, 217 m, 6 Jun 1997, 1 male in alcohol, USNM 1123366; *Dominator* 971–135, 51°37'22"N, 178°35'E, 163 m, 14 Jul 1997, 1 female, USNM 1123364; *Let's Go*, 51°57'N, 178°11'E, 0–247 m, 30 Aug 1986, 3 males, USNM 96259; *MF* 801–70, 52°03'24"N, 179°25'06"E, 174 m, 29 Jul 1980, 1 female, USNM ; *MF* 833–47, 51°55'36"N, 176°52'48"W, 201 m, 5 Aug 1983, 1 male, USNM 77048; *Ocean Olympic*, 52°04'31"N, 177°12'28"E, 293 m, 4 Mar 2000, 1 male, USNM 1122540; *Pacific Knight* 941–36, 53°02'N, 170°13'W, 0–178 m, 11 Jun 1994, 8 male, USNM 96265, 96525, and 96542; *Pacific Knight* 941–37, 52°17'N, 170°40'W, 0–155 m, 12 Jun 1994, 1 male, USNM 96269; *Pacific Knight* 941–40, 52°41'N, 170°49'W, 0–126 m, 12 Jun 1994, 1 male, 1 indet., USNM 96270 and 96534; *Pacific Knight* 941–42, 52°55'N, 170°24'W, 0–225 m, 13 Jun 1994, 1 male, USNM 96522; *Pacific Knight* 941–49, 52°46'N, 171°45'W, 0–340 m, 14 Jun 1994, 1 female, USNM 96521; *Pacific Knight* 941–121, 51°38'N, 178°19'W, 0–375 m, 5 Jul 1994, 3 female, USNM 96262; *Pacific Knight* 941–204, 5306'N, 171°42'E, 9–455 m, 31 Jul 1994, 1 female, USNM 96523; *Sea Storm* 90, 51°36'30"N, 177°10'48"W, 217 m, 4 Jul 2002, 1 male in alcohol, USNM 1123009; *Sea Storm* 91, 51640'51"N, 177°10'49"W, 82 m, 4 Jul 2002, 1 male, USNM 1123021; *Sea Storm* 100, 51°42'59"N, 175°47'07"E, 86–94 m, 7 Jul 2002, 1 indet. in alcohol, USNM 1076928; *Sea Storm* 101, 51°45'27"N, 175°40'06"E, 83 m, 7 Jul 2002, 1 female, 2males, USNM 1123024, -26, -27; *Sea Storm* 105, 52°08'59"N, 175°06'47"E, 201 m, 8 Jul 2002, 1 male, USNM 1123023; *Sea Storm* 111, 52°16'20:N, 175°29'13"E, 137 m, 9 Jul 2002, 1 male and 1 female in alcohol, USNM 1123000, 1123013; *Sea Storm* 113, 52°03'07"N, 177°23'55"E, 128 m, 10 Jul 2002, 1 male, USNM 1123007; *Sea Storm* 115, 52°04'16"N, 177°19'04"E, 165 m, 10 Jul 2002, 1 indet. in alcohol, USNM 1123020; *Sea Storm* 116, 52°04'10"N, 177°14'25"E, 87–94 m, 11 Jul 2002, 1 female, USNM 1123006; *Sea Storm* 118, 52°00'14"N, 177°49'40"E, 104–111 m, 11 Jul 2002, 1 female, USNM 1123283; *Sea Storm* 122, 52°02'49"N, 179°25'18"E, 143 m, 13 Jul 2002, 6 male colonies, some in alcohol, and SEM stub 1499, USNM 1123043–44, 1123046, 1123048–49; *Sea Storm* 124, 52°16'24"N, 179°55'47"E, 88 m, 13 Jul 2002, 1 male in alcohol; *Sea Storm* 129, 51°53'24"N, 179°44'07"E, 84–93 m, 14 Jul 2002, 1 male, USNM 1123032; *Sea St*orm 130, 52°12'10"N, 176°12'40"E, 86 m, 15 Jul 2002, 1 female, USNM 1123002; *Sea Storm* 146, 52°15'55"N, 174°48'02"W, 113 m, 20 Jul 2002, 1 male in alcohol, USNM 1123019; *Shishaldin*, 51°53.84'N, 177°44.88'W, 374 m, Feb 2000, 1 male, USNM 1122495; *Shishaldin*, 52°27'14"N, 179°30'54"E, 218 m, 2 Feb 2000, 1 female, USNM 1122521; *Vesteraalen* 3, 52°38'12"N, 169°46'35"W, 75 m, 21 May 2000, 1 female, USNM 1123373; *Vesteraalen* 5, 54°40'43"N, 169°06'11"W, 102 m, 21 May 2000, 1 female, 1 male, USNM 1123372; *Vesteraalen* 8, 53°09'28"N, 167°04'14"W, 154 m, 22 May 2000, 1 male in alcohol, USNM 1123371; *Vesteraalen* 941–36, 52°56'N, 169°31'W, 0–227 m, 10 Jun 1994, 1 male, USNM 96261; *Vesteraalen* 941–151, 52°10'N, 179°44'E (topotypic), 0–90 m, 10 Jun 1994, 1 female, USNM 96267; *Vesteraalen* 941–153, 52°10'N, 179°43'E (topotypic), 0–94 m, 10 Jun 1994, 2 female, USNM 96268; *Vesteraalen* 941–167, 51°54'N, 178°20'E, 0–150 m, 19 Jul 1994, 3 male, SEM stub 1498, USNM 96264; *Vesteraalen* 941–185, 52°03'N, 176°31'E, 0–91 m, 24 Jul 1994, 1 female, USNM 96263; *Vesteraalen* 941–210, 53°07'N, 170°56'E, 0–92 m, 30 Jul 1994, 2 males, USNM 96266; University of Washington, 51°32'N, 179°15'W, 278–289 m, 1 Sep 1968, 1 female, USNM 96250; McClusky, coll., 51°53'14"N, 179°47'50"E, 351–393 m, 30 Mar 2000, 1 female, USNM 1122448; off Sharma, AK, depth unknown, 2 female, USNM 76539;

##### Description.

Colonies variable in shape, ranging from planar (Fig. 11G) to multi-planar to bushy (Fig. 11A, E), the growth form possibly moderated by commensals (see Remarks). Branch tips usually blunt and circular to slightly flattened in cross section, 2.5–3.0 mm in diameter, more slender if affected by commensals; branch anastomosis not uncommon. Largest colony (USNM 96523) 28 cm in height; colonies attached by massive, dense basal branches up to 4.2 cm in diameter (USNM 96262). Coenosteum reticulate-granular in texture, coenosteal strips 50–60 µm wide, separated by thin slits about 10 µm wide; granules rounded, 7–8 µm in diameter. All colonies infested with spionid polychaetes (*Polydora*) and their characteristic binary axial tubes (see Remarks). Coenosteum pale orange.

**Figure 13.**
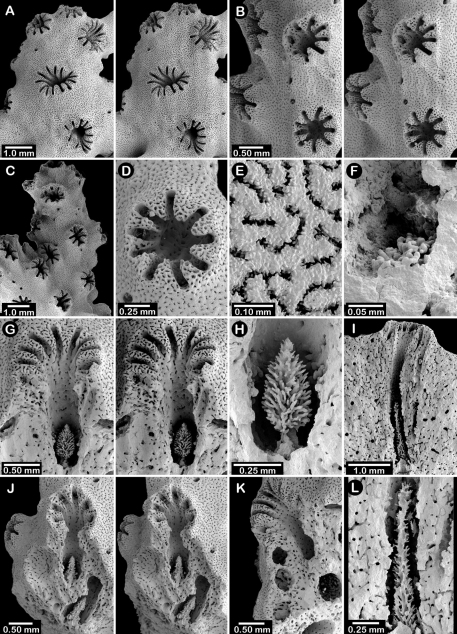
*Stylaster brochi*, **A, E, G–H, K** male from USNM 96264 **B–D, F, J** female syntype, USNM 43264 **I, L** male from USNM 1123049: **A** stereo view of cyclosystems on branch tip **B** stereo view of cyclosystems and supernumerary dactylopores **C** branch tip **D** a cyclosystem **E** coenosteal texture **F** dactylostyle **G** stereo view of longitudinal section through a cyclosystem **H** a gastrostyle surrounded by its ring palisade **I, L** an elongate gastrostyle **J** longitudinal section of a cyclosystem showing curvature of gastropore tube, sectioned female ampulla, and two gastrostyles **K** cross section through several male ampullae and a gastropore tube.

Cyclosystems circular, 0.9–1.1 mm in diameter, and slightly (about 1 mm) raised above coenosteum; cyclosystems uniformly arranged on three or all four sides of a branch, rarely in a linear fashion. Gastropores 0.35–0.40 mm in diameter. Gastropore tubes cylindrical, curved (Fig. 13J), and long (often 2–3 times length of gastrostyle), the length and curvature of tube making it difficult to see gastrostyle tip when viewed from above. Ring palisade absent or diffuse, the elements about 50 µm in height and diameter. Gastrostyles lanceolate and up to 0.7 mm in height, bearing long spines up to 70 µm in length.

Dactylotomes 80-110 µm in width; dactylostyles poorly developed, the cylindrical elements about 20 µm in height and 8 µm in diameter. Range of dactylopores per cyclosystem 6–13 (n = 50, average = 9.22 (σ = 1.43), and mode = 10). Supernumerary dactylopores quite common on coenosteum (Fig. 13A–C) and even on pseudosepta, presenting as small (0.09-0.11 mm in diameter), circular, slightly raised (rimmed) pores. Pseudosepta variable in width (Fig. 13D), in the same cyclosystem varying between 0.11 and 0.30 mm; adcauline diastemas also frequently present, each about twice pseudoseptal width.

Female ampullae (Fig. 13J) superficial hemispheres 0.8-1.1 mm in diameter; efferent pores rarely expressed, but if present, occurring in lateral position and somewhat recessed into coenosteum, each about 0.25 mm in diameter. Male ampullae (Fig. 13K) primarily internal, visible on coenosteal surface only as low mounds 0.3-0.5 mm in diameter, often with a small (30 µm diameter) apical efferent pore. Male ampullae often occur in high density clusters, directly adjacent to one another.

##### Remarks.

Virtually every colony of *Stylaster brochi* examined contained commensal spionid polychaetes of the genus *Polydora* (K. Fauchald, per. comm.) , such that this character was informally used to distinguish it from other *Stylaster* species, such as *Stylaster campylecus*. Almost every branch is bored axially by this robust worm, which forms binary longitudinal tubes 0.7-0.8 mm in diameter that are figure 8-shaped in cross section (Fig. 15K). Several worms may occur in the same colony, each having one or more efferent openings somewhere on the colony surface. It is easy to conjecture an advantage to the worm in this relationship, i.e. a secure place to live and protection from predators, but it is difficult to imagine an advantage to the stylasterid, as the worm must weaken the strength of the branches as well as compete for the same filtered plankton in the water. Thus, it would seem that these polychaetes are parasites on the coral. Large colonies of *Stylaster brochi* also form substantial three dimensional habitats for a variety of invertebrates, including sponges, bryozoans, hydroids, barnacles, bivalves, and ophiuroids. Heavy encrustation by sponges seems to promote a more delicate colony growth form, in which the terminal branches are only 1.5 mm in diameter (Fig. 11E). In a monograph otherwise devoted to stylasterids from Sagami Bay, Japan, Eguchi (1968) inexplicably described *Allopora abei* from “the Aleutian Islands.” Although the type was not examined, and Cairns (1983b) suggested a synonymy with *Stylaster polyorchis* Fisher, 1938, the illustrations provided by Eguchi suggest a synonymy with *Stylaster brochi*.Of 86 colonies examined, 35 are female, 40 male, and 11 indeterminate, resulting in a fairly equal sex ratio.*Stylaster brochi* is one of the most common and one of the largest growing stylasterids in the Aleutian Islands. It can be distinguished from other Alaskan species in *Stylaster* (Group A) by its usually planar growth mode, higher number of dactylopores per cyclosystem, and variable width of its pseudosepta (see Table 2 for additional comparisons).

##### Distribution.

Widespread throughout Aleutian Islands from west of Attu Island to Unalaska (including Petrel Bank), with one disjunct record near Sharma (near Anchorage); 75–351 m, but most records between 100–200 m.

**Table 2.** Tabular Key of the Northeast Pacific Species of *Stylaster* (AI= Aleutian Islands, BB=Bowers Bank, PB=Petrel Bank, GOA=Gulf of Alaska, CS=Cyclosystem, PS=Pseudosepta, GS=gastrostyle, DP=Dactylopore)

	*Stylaster brochi*	*Stylaster stejnegeri*	*Stylaster verrillii*	*Stylaster repandus*	*Stylaster venustus*	*Stylaster californicus*	*Stylaster campylecus*	*Stylaster leptostylus*	*Stylaster trachystomus*	*Stylaster parageus parageus*	*Stylaster parageus columbiensis*	*Stylaster alaskanus*	*Stylaster elassotomus*
Colony: Shape; Branching	Planar to bushy; Branch tips blunt	Arborescent; Branch tips blunt	Arborescent; Branch tips blunt	Lamellar (solid)	Planar to slightly bushy; Slender, blunt	Arborescent, large; Blunt tipped	Planar; Delicate	Planar, dichotomous; Delicate	Planar; Delicate branches	Bushy (multi-flabellate); Verydelicate	Bushy; Delicate	Planar; Branches forming sieve-like reticulum	Bushy; Delicate
Coenosteal Color	Orange	Orange, Pink	Light orange	Light orange, pink	Violet, Pink	Rose, red, purple	White, orange, pink	White	Pale orange, pink	White	White	Orange, pink, white	White
Spionid Worm Tubes	Present	Present	Present	Present	Present	Present	Absent	Absent	Common	Common	Absent	Absent	Absent
Arrangement of Cyclosystems	All sides of branch	All sides of branch	All sides of branches	Transverse rows	All sides of branches	All sides of branches	Branch edges and anterior face	Branch edges and anterior face	Branch edges and anterior face	Branch edges and anterior face	Branch edges and anterior face	Only on branch edges	Only on branch edges
Cyclosystems: Shape and Size	Circular; 0.9-1.1 mm	Circular; 1.0-1.2 mm	Circular; 1.0-1.2 mm	Circular; 1.0-1.15 mm	Polygonal, raised; 0.7-0.9 mm	Circular or irregular; 0.7-1.0 mm	Variable, slightly flared; 1.0-1.3 mm	Circular; 1.0-1.1 mm	Asymmetrical, slightly curved; Not visible	Circular; 0.9-1.0 mm	Circular to elliptical; 1.1-1.5 mm	Circular to irregular; 0.9-1.3 mm	Circular to elliptical; 1.0-1.2 mm
Gastropore Tube: Shape; Visibility of GS Tip	Cylindrical, curved; Difficult to see	Cylindrical, curved; Not visible	Straight rounded upper, cylindrical lower); Visible	Funnel-shaped, straight; Visible	Straight rounded upper, cylindrical lower); Visible	Straight, funnel-shaped; Visible	Cylindrical, curved; Difficult	Funnel-shaped above, cylindrical below; Visible	Funnel-shaped above, cylindrical below; Visible	Cylindrical, curved; Not visible	Funnel-shaped upper, cylindrical lower;Visible	Cylindrical, straight; Easilyvisible	Cylindrical, curved; Rarely seen
Ring Palisade	Absent or rudimentary	Absent	Robust	Rudimentary	Robust	Present	Absent to rudimentary	Absent	Rudimentary	Well-developed	Well-developed	Well-developed	Rudimentary
Dactylopores/Cyclosystem; Supernumerary Dactylopores	6-13 (x=9.22); Common	5-11 (x=6.46); Common	5-10 (x=7.10); Absent	1-11 (x=3.94); Absent	4-8 (x=5.70); Absent	3-8 (x=5.52); Common	7-17 (x=11.94); Absent	7-12 (x=9.76); Absent	8-18 (x=11.82); Rare	5-11 (x=8.54); Rare	6-13 (x=9.38); Rare	7-14 (x=11.30); Absent	11-17 (x=14.40); Common
Dactylostyles	Rudimentary	Rudimentary	Robust	Robust	Robust	Robust	Rudimentary	Moderate	Rudimentary	Well-developed	Well-developed	Rudimentary	Rudimentary
Ampullae: Female; Male	Superficial; Partially internal	Superficial, ridged; Unknown	Bothinternal	Superficial; Primarily internal	Bothinternal	Bothinternal	Bothsuperficial	Bothsuperficial	Superficial, papillose; Superficial	Superficial; Partially internal	Both superficial	Superficial, ridged; Superficial	Unknown; Superficial
Other Characters	Pseudosepta of variable width	Coenosteal papillae common	Coenosteal papillae common		Coenosteal papillae common; Cssoccasionally linked	Coenosteal papillae common; Css often linked	Coenosteal strips linear near css		Ps porous and wide; Coenosteal ridges	Gastropores only 0.25-0.30 mm in diameter	Gastropores 0.45-0.50 mm in diameter	Coenosteal papillae occassionally	Dactylotomes very shallow

#### Stylaster stejnegeri

(Fisher, 1938)

http://species-id.net/wiki/Stylaster_stejnegeri

Figs 11C 14A–J

Allopora stejnegeri Fisher, 1938: 518-519, pl. 42, figs 2–2b, pl. 56.—Boschma 1957: 28.
Stylaster stejnegeri : Cairns 1983b: 429.—Wing and Barnard 2004: 11, 28.—Heifetz et al. 2005” 134, 137 (listed).—Stone and Shotwell 2007: 108 (listed).—Jameison et al. 2007: 224 (listed).

##### Type material.

Holotype, 1 female colony, and SEM stub 1500, dry, USNM 43271 (Fig. 11C). **Type locality.** *Albatross* 4777: 52°11'N, 179°49'E (Petrel Bank, Aleutian Islands), 79–95 m.

##### Material examined.

Holotype.

##### Description..

The holotype (Fig. 11C) is a small, arborescent colony 6 cm in height and 7 cm in width, with a basal branch diameter of 9.3 mm. Branches cylindrical and blunt tipped, the tips measuring 3-5 mm in diameter. Coenosteum reticulate-granular in texture, coenosteal strips 50-55 µm wide, separated by slits 13-15 µm wide. Coenosteum also covered with small papillae (nematopores). Binary spionid worm tubes present along branch axes. Coenosteum light orange to pink.

Cyclosystems on all sides of branches, circular, 1.0–1.2 mm in diameter. Gastropores circular, 0.35–0.45 mm in diameter. Gastropore tube cylindrical and usually slightly curved such that gastrostyle tip is not visible from apical view; ring palisade absent. Gastrostyle lanceolate.

**Figure 14.**
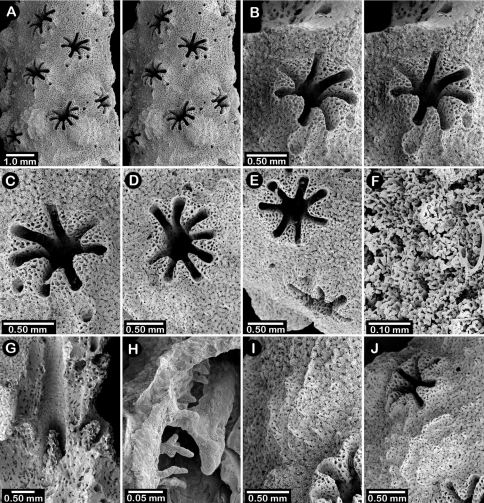
Holotype of *Stylaster stejnegeri*, USNM 43271: **A** stereo view of branch with cyclosystems, female ampullae, and supernumerary dactylopores **B** stereo view of a cyclosystem **C–E** cyclosystems with adjacent supernumerary dactylopores **F** coenosteal texture **G** longitudinal section of a gastropore tube **H** dactylostyles **I–J** ridged female ampullae.

Cyclosystems flush to only slightly raised above coenosteum; surface of pseudosepta porous. Dactylotome width 0.08-0.11 m. Range of dactylopores per cyclosystems 5-11 (n = 50, average = 6.46 (σ = 1.03), and mode = 6). Small (0.11–0.14 mm diameter), circular supernumerary dactylopores fairly common (Fig. 14A-C). Dactylostyles rudimentary (Fig. 14H).

Female ampullae (Fig. 14A, I–J) superficial hemispheres up to 1.1 mm in diameter, often bearing low ridges radiating from their apex. Male ampullae unknown.

##### Remarks.

Although only one specimen is known of this species, it does appear to be distinctive. Perhaps most similar to *Stylaster brochi*, it differs from that species in having flush cyclosystems, fewer dactylopores per cyclosystem, and ridged female ampullae (Table 2).

##### Distribution.

Known only from one specimen from the type locality.

#### Stylaster verrillii

(Dall, 1884)

http://species-id.net/wiki/Stylaster_verrillii

Figs 11B, F, H 15A–M

**Figure 15.**
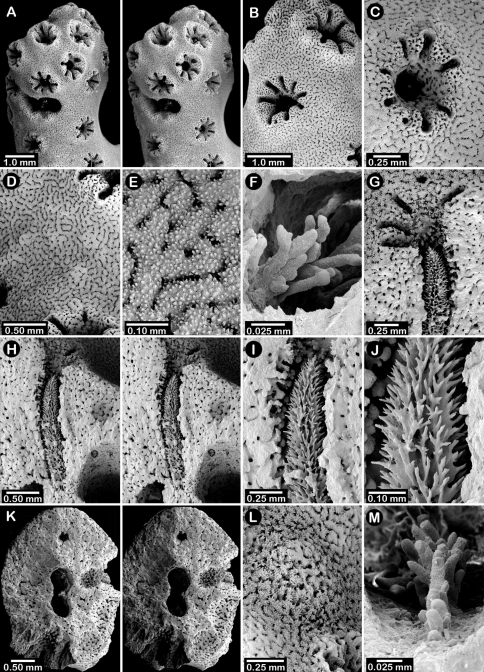
*Stylaster verrillii*, **A, F, L–M** male from USNM 76524, **B,** **G–J** male from USNM 1123517, **C–E** USNM 1123299, **K** female from USNM 76528: **A** stereo view of blunt branch tip showing cyclosystems and spionid tubes **B–C** cyclosystems **D–E** coenosteal texture **F, M** dactylostyle **G–J** lateral view of gastrostyles (H a stereo view) **K** stereo view of axial spionid tubes and cross section of female ampullae **L** male ampulla.

Allopora verrillii Dall, 1884: 111-113.—Fisher 1931: 391–392.
Allopora moseleyi Dall, 1884: 113.—Fisher 1931: 391-392.
Allopora verrilli : Fisher 1938: 521-522, pl. 54, fig. 3, pl. 57, pl. 76, figs 5–6.—Naumov 1960: 567, text-fig. 411.
Stylaster (Allopora) norvegicus forma *pacifica*Broch 1936: 52, text fig. 15c-d (in part: record from Snake Island, Strait of Georgia, British Columbia).
Stylaster (Allopora) verrillii : Broch 1942: 6.
Allopora norvegicus pacifica .—Lowenstam 1964: 382 (mineralogy).
Stylaster verrillii : Cairns 1983b: 429.—Heifetz 2002: 22 (listed).—Wing and Barnard 2004: 11, 28, fig. 31.— Heifetz et al. 2005: 134, 137.—Stone and Shotwell 2007: 108 listed).—Brooke and Stone 2007: 522, fig. 2B.—Jameison et al. 2007: 224 (listed).— Lindner et al. 2008: 3, supplemental Table S1 (phylogeny and DNA sequences).
Stylaster ?norvegicus .—Jameison et al. 2007: 224 (listed).

##### Type material.

*Allopora verrillii*: 5 badly worn, dry, female syntypes, the largest 44 mm in diameter (USNM 4193, Fig. 11H). All branches are eroded from the colony, thus resembling an encrusting colony. Collected together with four dry topotypic specimens (USNM 8850).

##### Type locality.

Chika Island, Akutan Pass, near Unalaska, Aleutian Islands, on beach.

*Allopora moseleyi*: Holotype, 1 small (18 mm) dry colony, USNM 6851 (Fig. 11F).

##### Type locality.

Kiska Harbor, Kiska Island, Aleutian Islands, on beach.

##### Material examined.

Types of the two species; *Alb-*4777, 52°11'N, 179°49'E, 95 m, 5 June 1906, 1 female, 1 male, 1 indet., and SEM stub 1501, USNM 53394 and 76524 (mentioned by Fisher 1938); unnumbered *Albatross* station, Sucia Islands, near San Juan Island, Washington, 16 Sep 1890, 2 female, 11 male colonies, and SEM stub 1513, USNM 76526–28 (mentioned by Fisher 1938); *Delta* 5622, 51°57'36.54"N, 176°49'58.32"W, 140 m, 24 Jul 2002, 1 male, and SEM stub 1503, USNM 1123299; *Delta* 5625, 51°57'42"N, 176°50'01"W, 155 m, 25 Jul 2002, 1 male, USNM 1027819; *Patricia Lee*, 51°53.44'N, 179°47.7'E, 298 m, 1 indet., AB00–41b; *Sea Storm* 122, 52°02'49"N, 179°25'18"E, 143 m, 13 Jul 2002, 2 female colonies in alcohol, USNM 1123058 (attached to *Distichopora borealis*, USNM 1123061); *Sea Storm* 123, 52°10'53"N, 179°37'02"E, 124 m, 13 Jul 2002, 3 female, 2 male, 1 indet., USNM 1123053, 1123056; *Sea Storm* 129, 51°52'24"N, 179°44'07"E, 84–93 m, 14 Jul 2002, 1 male in alcohol, USNM 1123051; Sea Storm 155, 52°38'43"N, 172°27'27"W, 393 m, 22 Jul 2002, 1 female, USNM 1122763; *Vesteraalen* 941–40, 52°53'N, 169°59'W, 0–62 m, 11 Jun 1994, 1 female, USNM 96260; *Vesteraalen* 941–151, 52°10'36"N, 179°43.7'E, 87–92 m, 10 Jul 1994, 1 male, and SEM stub 1502, USNM 1123537; south of Semisopochnoi Islands, 366 m, 1 indet., USNM 88368; Ralston Island, near Juneau, depth unknown, 1 indet., AB04–21; Stubbs Island, BC, depth unknown, 2 indet., AB02–02; Wooden Island, AK, depth unknown, 2002, 1 dry, AB02–150b; Middle Cross Sound, AK, 15–21 m, 27 Jul 1978, 1 indet., AB78–120a; North Pass, Lincoln Island, AK, depth unknown, 2 indet. in alcohol, AB05–37.

##### Description.

Colonies arborescent, dichotomously branching to form a three dimensional bush; branch tips blunt to slightly clavate, 3.0–4.5 mm in diameter. Largest colony (USNM 1027819, Fig. 11B) 3.5 cm tall, 7.0 cm wide, and attached by a basal branch 11 mm in diameter. Colonies usually attached to stones or bivalve shells. Coenosteum reticulate-granular in texture, coenosteal strips 52–55 µm wide, separated by thin slits 10–13 µm wide. Strips covered with small (8–10 µm in diameter) spines, occurring 4–5 across width of a strip, conferring a fine, granular or “sugary" texture. Coenosteal papillae common. Most colonies infested with spionid worms and their characteristic binary tubes (Fig. 15A, K). Coenosteum light orange.

Cyclosystems occur on all sides of branches, circular, and 1.0–1.2 mm in diameter. Gastropore circular and small (0.25–0.30 mm in diameter). Gastropore tubes funnel-shaped near surface but cylindrical adjacent to gastrostyle, straight, and often quite long in thick branches. Ring palisade robust near gastrostyle tip, composed of squat, clavate elements about 40 µm in diameter. Gastrostyles lanceolate to elongate (up to 1.5 mm, Fig. 15H), with a pointed tip; gastrostyles covered with anastomosing, longitudinal, spiny ridges, the spines quite long (up to 65 µm in length) and sharp.

Cyclosystems flush to only slightly raised above coenosteum. Dactylotomes 0.07–0.10 mm in width, sometimes becoming obsolete within a system by infilling of the axial slit, resulting in just an apical pore. Dactylostyles robust (Fig. 15F, M), composed of tall slender cylindrical elements up to 60 µm in height and 10–15 µm in diameter. Range of dactylopores per cyclosystem 5–10 (n = 50, average = 7.10 (σ=1.16), and mode = 7). Pseudosepta triangular; diastemas rare.

Female ampullae primarily internal (Fig. 15K), with only a slight superficial swelling; internal diameter of ampullae 0.7–0.8 mm. Efferent pores of female ampullae never observed. Male ampullae (Fig. 15H, L) also primarily internal, visible on surface only as small dimples about 0.4 mm in diameter; internal cavity diameter 0.3–0.5 mm.

##### Remarks.

**Figure 16.**
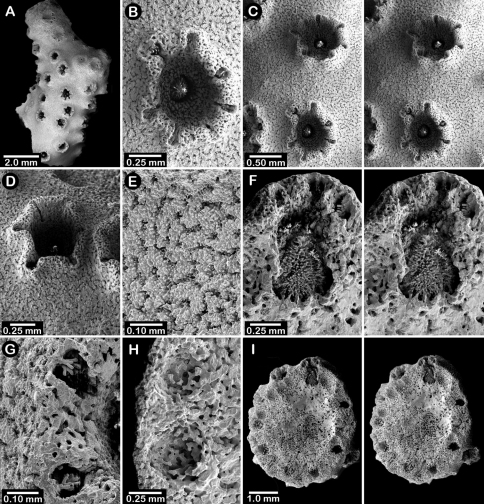
*Stylaster venustus*, **A, E–I** USNM 76545, **B–D** holotype, MCZ 5525: **A** blunt branch tip **B–D** cyclosystems (C a stereo view) **E** coenosteal texture **F** stereo view of a gastrostyle and ring palisade **G** two dactylostyles **H** cross section of two internal male ampullae **I** stereo view of branch cross section showing many internal male ampullae.

**Figure 17.**
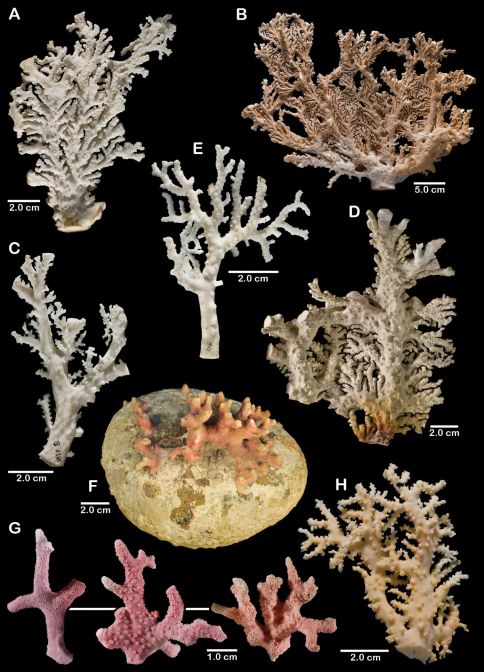
Color figures of skeleton of various Alaskan stylasterids: **A–D** *Stylaster campylecus* **E** *Stylaster leptostylus* **F–G** *Stylaster venustus* **H** *Stylaster trachystomus* **A** holotype, USNM 42870 **B** holotype of *Allopora polyorchis*, USNM 43266 **C** syntype of *Allopora campyleca tylota*, USNM 86000 **D** holotype of *Allopora moseleyanus*, USNM 42869 **E** holotype of *Allopora moseleyanus leptostylus*, USNM 43270 **F** holotype, MCZ 5525 **G** three branches, USNM 76545 **H** holotype, USNM 43265.

*Stylaster verrillii* is similar to *Stylaster venustus* (Figs 16A–I, 17F–G), but differs in having orange, bushy coralla (not pink to purple planar colonies, Fig. 17G); larger cyclosystems (1.0–1.2 mm vs 0.7–0.9 mm in diameter); larger diameter and blunt distal branches; triangular (not rectangular, Fig. 16B) pseudosepta; and straight-sided gastropore tubes (not hemispherical, Fig. 16B)(also see Table 2). Both species are confined to relatively shallow water and have overlapping distributions off British Columbia and Washington. According to the ICZN (1999) article 31.1.3, the original spelling *verrillii* should be preserved in preference to *verrilli*.

##### Distribution.

Known from Kiska Harbor, Aleutian Islands to Sucia Islands, Washington; 21-393 m, although most records between 60-155 m.

#### Stylaster repandus

Lindner & Cairns sp. n.

urn:lsid:zoobank.org:act:255EF5E7-5BFA-428A-A5B9-CC47C1A28385

http://species-id.net/wiki/Stylaster_repandus

Figs 27A–C 28A–M

##### Type material.

Holotype: 1 large dry male colony, and SEM stub 1504, USNM 1122740 (Fig. 27A-B). Paratypes: 52°21.20'N, 171°02.77'W, 475 m, 1 Sep 2000, 1 dry female colony, and SEM stub 1505, USNM 1122739; Amukta Pass, depth unknown, 1996, 2 dry male colonies, USNM 1122741. **Type locality.** 52°17.86'N, 170°58.28'W (southeast of Amukta Island), 375 m, 12 Sep 2000.

##### Etymology.

The specific name *repandus* (from the Latin, meaning “bent backwards”, “undulate”) refers to the complexly folded coenosteal lamellae of this species.

##### Material examined.

Types.

##### Description.

Colonies firmly attached to a hard substrate by a robust basal stem and encrusting base, the base being 9 cm in diameter and the basal stem 5 cm in diameter in the case of the holotype. Immediately above basal stem the colony divides into two or three lamellae (Fig. 27C), each lamella (or sheet) of coenosteum increasing its surface area by folding its surfaces into a complex three-dimensional structure similar to that of *Cyclohelia*. Short (up to 15 mm) cylindrical branches project from plane of colony, but always in response to housing a parasitic spionid worm tube. Holotype (Fig. 27A–B) 23 cm tall and 19 cm in width. Coenosteal lamellae thin at edge, only 2.0–2.5 mm thick, and thus easily damaged during collection; basal lamellae up to 10 mm in thickness. All colonies examined heavily infested with spionid worm tubes. Coenosteum reticulate-granular in texture, the strips being about 30 µm wide, bordered by slits about 9–19 µm thick; granules spinose. Coenosteum covered uniformly with small papillae (Fig. 28B). Coenosteum light orange, but central core a light shade of pink.

Cyclosystems equally common on both faces of lamellae, and even occur on lamellar edges, often arranged in transverse rows of up to 15 cyclosystems that parallel lamellar edge; diameter of cyclosystems 1.0–1.15 mm. Gastropore tube funnel-shaped near branch surface, and straight but constricted, the upper diameter being about 0.34 mm in diameter, the constriction corresponding to a diffuse ring palisade, the blunt elements being up to 60 µm tall and 40 µm in diameter. Gastrostyles lanceolate, pointed, and bear longitudinal, spinose ridges. Illustrated style (Fig. 28D) 0.53 mm in height and 0.26 mm in basal diameter.

Cyclosystems almost flush with coenosteum, the dactylopore slits raised only slightly above coenosteum; dactylotomes 0.15-0.17 mm in width. Based on 74 cyclosystems the range of dactylopores per cyclosystem is 1-11 (average = 3.94 (σ = 2.05), and mode = 4). In many cyclosystems there is an adcauline diastema (Fig. 28E), often produced by the infilling of 1–3 adcauline dactylopores, these dactylopores becoming obsolete. Dactylostyles robust, composed of an almost unilinear arrangement of cylindrical, blunt to clavate elements up to 60 µm tall and 13–15 µm in diameter (Fig. 28D, H, J–K).

Female ampullae (Fig. 28L–M) low swellings on coenosteum 0.9–1.1 mm in diameter (internal diameter 0.7–0.8 mm), each with a peripheral efferent pore 0.3–0.4 mm in diameter that faces upward. Male ampullae (Fig. 28I) densely arranged, superficially visible only as inconspicuous mounds 0.5–0.6 mm in diameter (internal diameter 0.4–0.5 mm), often with an irregularly shaped apical efferent pore about 60 µm in diameter.

##### Remarks.

Among the 80 Recent species in the genus *Stylaster* (Appeltans et al. 2011: www.marinespecies.org), *Stylaster repandus* is the only one to have a lamellate growth form. It is very similar in growth form to *Cyclohelia lamellata* and *Errinopora undulata*, and is one of five stylasterid species to have lamellate colonies (see Discussion of *Errinopora undulata*).

##### Distribution.

Known only from three localities in the vicinity of Amukta Island, Aleutian Islands; 375–475 m.

### *Stylaster* (Group B)

**Diagnosis.** Species of *Stylaster* in which coralla form branching colonies, and in which cyclosystems are arranged primarily on branch edges, but also on anterior and occasionally posterior sides of branches; branches usually delicate.

**Discussion.** Called the “annectant” group by Cairns (1983b) because it links the morphology of Groups A and C regarding cyclosystem arrangement.

#### Stylaster campylecus

(Fisher, 1938)

http://species-id.net/wiki/Stylaster_campylecus

Figs 17A–D 18A–M

Allopora campyleca Fisher, 1938: 505–506, pls. 34, 36.—Broch 1942: 101.—Boschma 1957: 19–20.—Not Lowenstam 1964: 382 (probably *Stylaster parageus parageus*).
Allopora polyorchis Fisher, 1938: 503–505, pl. 35, fig. 1, pl. 37, 38.—Naumov 1960: 581.—Boschma 1957: 27.
Allopora campyleca tylota Fisher, 1938: 509–510, pl. 41, figs 2–2e.—Naumov 1960: 580.
Allopora moseleyana Fisher, 1938: 512–514, pl. 49, fig. 2, pl. 51 (not pl. 50), pl. 53, fig. 1 (not forma *leptostyla*).—Boschma 1957: 23.—?Naumov 1960: 577.
Allopora campyleca campyleca .—Naumov 1960: 578.
Stylaster campylecus .—Cairns 1983b: 430.—Not Cairns and Macintrye 1992: 100 (probably *Stylaster parageus parageus*).—Brooke and Stone 2007: 523, 524, fig. 2C.—Not Lindner et al. 2008: 3, Table S1 (=*Stylaster brochi*).
Stylaster moseleyanus .—Cairns 1983b: 429.—Wing and Barnard 2004: 11, 28, fig. 30.—Heifetz et al. 2005: 124137 (listed).—Stone and Shotwell 2007: 108 (listed).—Jamieson et al. 2007: 224 (listed).
Stylaster polyorchis .—Cairns 1983b: 430.—Cairns and Macintrye 1992: 100–101 (mineralogy).—Heifetz 2002: 22 (listed).—Wing and Barnard 2004: 11, 28.—Heifetz et al. 2005: 134, 137 (listed).—Stone and Shotwell 2007: 108 (listed).—Jamieson et al. 2007: 224 (listed).—Not Lindner et al. 2008: 3, Table S1 (=*Stylaster brochi*).
Stylaster campylecus tylotus .—Cairns 1983b: 430.—Wing and Barnard 2004: 27 (listed).—Heifetz et al. 2005: 134 (listed).—Stone and Shotwell 2007: 108 (listed)—Jameison et al. 2007: 224 (listed).
Stylaster campylecus campylecus .—Wing and Barnard 2004: 11, 27, fig. 32.—Heifetz et al. 2005: 134 (listed).—Stone and Shotwell 2007: 108 (listed)—Jamieson et al. 2007: 224 (listed).

##### Type material.

*Allopora campyleca*: Holotype: *Alb*-3480, 1 dry male colony, now in two pieces, length 16 cm, and SEM stub 1524–25, 1545, USNM 42870 (Fig. 17A). Paratypes: *Alb*-3480, 7 dry colonies and 7 in alcohol, and SEM stub 1526, USNM 43767, 52262, 52265, 58099, and CAS 28297; *Alb*-2852 (missing in 2011); *Alb*-2858, 3 dry colonies (not considered conspecific with *Stylaster campyleca*); *Alb*-3599, 2 dry colonies (1 male is *Stylaster campylecus*, 1 female is *Stylaster brochi*), USNM 76814; *Alb*-4230, 1 dry male colony (not considered to be conspecific with *campyleca*); *Alb*-4302, 2 female dry colonies, USNM 76816. **Type locality.** *Alb*-3480: 52°06'N, 171°45'W (Amukta Pass, Aleutian Islands), 518 m.

*Allopora polyorchis*: Holotype: *Alb*-3480, 1 large dry colony now in two pieces, and SEM stubs 1527–28, USNM 43266 (Fig. 17B). Paratypes: *Alb*-3480: about 15 dry branch fragments, USNM 76540–76542, CAS 28293. **Type locality.** *Alb*-3480: 52°06'N, 171°45'W (Amukta Pass, Aleutian Islands), 518 m.

*Allopora campyleca tylota*: Syntypes: *Alb*-4781, 4 dry female colonies, the largest 15 cm in height, and SEM stub 1539, USNM 43263, and 12 dry male branches, and SEM stub 1540, USNM 86000 (Fig. 17C). **Type locality.** *Alb*-4781: 52°14'30"N, 174°13'E (off Agattu I., Near Islands, Aleutians), 882 m.

*Allopora moseleyana*: Holotype: *Alb*-4781: 1 dry male colony now in two pieces, largest 13 cm in length, USNM 42869 (Fig. 17D). Paratypes: *Alb*-4781, 1 dry male colony, USNM 86003; *Alb*-3480, 3 female and 1 male dry colonies, and SEM stub 1519, USNM 76556, 76557; *Alb*-3480, 2 dry female and 1 male colonies (considered as *Stylaster leptostylus*), USNM 76555. **Type locality.** *Alb*-4781: 52°14'30"N, 174°13'E (off Agattu I., Near Islands, Aleutians), 882 m.

##### Material examined.

Types of *Allopora campyleca*, *Allopora polyorchis*, *Allopora campyleca tylota*, and *Allopora moseleyana*; *Alb*-3480, numerous branch fragments (topotypic), USNM 76565; *Alaskan Leader*-35, 53°01'42"N, 170°05'59"W, 200–400 m, 4 Jun 2000, 1 female, USNM 1122510; *Alaskan Leader* 64, 53°07'N, 166°53'42"W, 324–766 m, 21 Jun 2002, 1 female, USNM 1122484; *Alb*-4781, type locality, 1 female, USNM 76700; *Ballyhoo*, 54°47'20"N, 178°46'53"E, 254 m, 5 Jun 2000, 1 female, USNM 1122535; *Ballyhoo*, 54°42.44'N, 178°46.83'E, 250 m, 5 Jun 2000, 1 female, AB00–0013; *Delta*, 51°50'55"N, 179°49'26"W, 125 m, 17 Jul 2002, 1 indet., USNM 1123463; *Delta Alfa*, western Aleutian Islands, depth unknown, 1961, 1 female, USNM 52447; *Dominator* 971–181, 51°27'43"N, 176°38'20"E, 384 m, 27 Jul 1997, 2 female, 2 male, USNM 1123363; *Jason* 2095–7-7, 51°48.693'N, 173°49.965'W, 843 m, 25 Jul 2004, 1 male, AB09–0013; *Jason* 2102–7-8, 51°30.56'N, 177°55.36'W, 824 m, 2 Aug 2004, 6 male, AB09–0012; *Jason* 2101–9-1, 51°31.43'N, 177°57.5'W, 494 m, 2 Aug 2004, 1 male, AB09–0015; *Jason* 2103–15–1, 51°50.968'N, 179°51.007'E, 678 m, 1 male, AB08–0037; *Jason* 2104–1-5, 51°43.83'N, 179°36.0'W, 1011 m, 5 Aug 2004, 1 female, 1 male, AB08–0038 and AB09–0016; *MF* 833–38, 51°42'54"N, 178°51'30"W, 582 m, 3 Aug 1983, 1 male in alcohol, USNM 77055; *MF* 833–47, 51°55'36"N, 176°52'48"W, depth unknown, 5 Aug 1983, 1 female, USNM 96529; *North Pacific*, 52°04'22"N, 176°58'01"E, 384 m, 20 Oct 2000, 1 male, USNM 1122539; *Ocean Olympic*, 52°09'06"N, 176°09'48"E, 384 m, 2000, 1 male, USNM 1122536; *Pacific Knight* 941–49, 52°46'N, 171°45'W, 0–340 m, 14 Jun 1994, 1 female, USNM 96528; *Pacific Knight* 941–75, 52°31'N, 173°30'W, 0–213 m, 20 Jun 1994, 1 male, USNM 96527; *Pacific Knight* 941–204, 53°06'N, 171°42'E, 0–455 m, 31 Jul 1994, 1 female, 1 male, USNM 96249 and 1123534; *Patricia Lee*, 52°21'09"N, 179°32'52"E, 375 m, 2000, 1 female, USNM 1122500; *Sea Storm* 90, 51°36'30"N, 177°10'48"W, 202–217 m, 4 Jul 2002, 1 male in alcohol, USNM 1123012; *Sea Storm* 91, 51°40'51"N, 177°10'49"W, 82 m, 4 Jul 2002, 1 male, USNM 1123284; *Sea Storm* 92, 51°33'34"N, 177°36'59"W, 367 m, 4 Jul 2002, 1 female in alcohol, 1 male, USNM 1123028–29; *Sea Storm* 107, 52°10'28"N, 175°14'14"E, 214 m, 8 Jul 2002, 1 female, 2 male in alcohol, USNM 1123001, -05, -31; *Sea Storm* 108, 52°11'32"N, 175°17'E, 208 m, 8 Jul 2002, 1 female, 1 male, both in alcohol, USNM 1122998 and 1123041; *Sea Storm* 109, 52°17'16"N, 175°20'56"E, 238 m, 8 Jul 2002, 2 indet. in alcohol, USNM 1122997 and 1123536; *Sea Storm* 111, 52°16'20"N, 175°59'13"E, 137 m, 9 Jul 2002, 1 male, 1123034; *Sea Storm* 112, 52°15'12"N, 176°00'42"E, 137 m, 9 Jul 2002, 1 indet., USNM 1122999; *Sea Storm* 134, 52°14'38"N, 176°01'25"E, 138 m, 15 Jul 2002, 1 indet. in alcohol, USNM 1123033; *Sea Storm* 138, 52°13'50"N, 175°14'31"E, 146 m, 16 Jul 2002, 1 female, 1 male, USNM 1122769 and 1122965; *Sea Storm* 148, 52°28'16"N, 173°19'10"W, 194 m, 21 Jul 2002, 2 male, USNM 1122748 and 1122752*; Sea Storm* 150, 52°30'47"N, 173°29'W, 213 m, 21 Jul 2002, 1 female, USNM 1122758; *Sea Storm* 151, 52°33'40"N, 173°19'10"W, 203 m, 21 Jul 2002, 1 female, 1 male, USNM 1122755–6; *Sea Storm* 155, 52°38'43"N, 172°16'23"W, 393–401 m, 22 Jul 2002, 2 indet., USNM 1122761, -64; *Shishaldin*, 54°07'N, 179°45'E, 366 m, 20 Feb 2000, 1 female, USNM 1122529; *Shishaldin*, 54°24'03"N, 179°36'11"E, 313 m, 25 Feb 2000, 1 female, USNM 1122532; *Shishaldin*, 53°56'54"N, 179°49'31"E, 318 m, 14 mar 2000, 1 indet., USNM 1122568; Chew, coll., 51°32'N, 179°15'E, 218–289 m, 1 Sep 1968, 3 female, USNM 76573 and 96532; Haaga, coll. 53, 51°24;12:N, 177°37'20"W, 12 Jun 2000, 1 female, USNM 1122515; Haaga, coll., 54, 51°45;48"N, 178°09'47"W, 441–791 m, 10 Jun 2000, 1 female, 1 male, USNM 1122496 and AB02–30; McCloskey, coll., 52°03'26"N, 179°12'20"E, 475 m, 27 Apr 2000, 2 indet., USNM 1122449 and 1122461; McCloskey, coll., 52°17'45"N, 179°28'57"E, 28 May 2000, 1 female, USNM 1122964; McCloskey, coll., 51°16'46"N, 178°56'01"E, 373 m 21 May 2000, 2 indet., USNM 1122463; Miller, coll., 51°31'09"N, 176°53'14"W, 375 m, 2000, 1 female, USNM 1122498; Myer, coll.,52°15'N, 173°30'W, 208–274 m, 20 Nov 1973, 4 female, USNM 76567; Slear, coll., 52°07'54"N, 177°14'46"E, 238 m, 8 Dec 2000, 1 female, USNM 1122453; Slear, coll., 51°54'04"N, 179°17'28"E, 567 m, 9 Dec 2000, 2 female, USNM 1122450, -51; Stone, coll., 51°51.133'N, 179°50.9'E, 630 m, 8 Aug 2005, 1 female, AB05–0055; Tierny, coll., Cross Sound, Alexander Archipelago, 334 m, 30 Jul 1953, 1 indet., USNM 76569;

##### Description.

**Figure 18.**
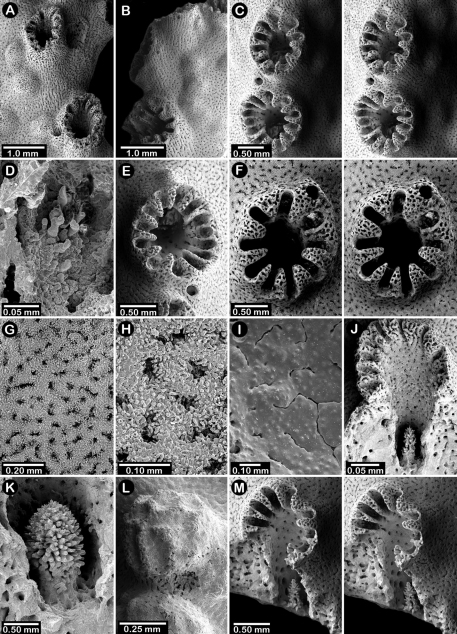
*Stylaster campylecus*, **A** male syntype of *tylota* form, USNM 86000, **B–H, M** male holotype of *campylecus*, USNM 42870, **I** male holotype of *polyorchis* form, USNM 43266**, J** female syntype of *tylota* form, USNM 43263, **K** male holotype of *moseleyanus* form, USNM 42869, **L** female paratype of *campylecus*, USNM 52262: **A–B** distal branches showing male ampullae and striate coenosteum **C** stereo view of two aligned cyclosystems **D** rudimentary dactylostyle **E–F** cyclosystems **G–H** porous coenosteal texture **I** porcellaneous coenosteal texture **J** longitudinal section of a cyclosystem **K** gastrostyle **L** female ampulla **M** stereo view of a damaged cyclosystems showing curved gastropore, small dactylostyles, and lack of a ring palisade.

Corallum essentially uniplanar or multiplanar, with occasional short side branches oriented perpendicular to flabellum. Branches anastomose only in larger colonies, the largest colony being the holotype of *Stylaster polyorchis*, which is 28 cm tall and 35 cm broad, having a massive, dense basal branch diameter of 3.9 cm (Fig. 17B). Distal branches circular to slightly flattened in cross section; larger-diameter branches often rectangular in cross section, the longer axis of the rectangle oriented perpendicular to plane of flabellum. Commensal spionid worm tubes not present. Coenosteum reticular-granular in texture, the coenosteal strips 50–55 µm in width, the slits being a discontinuous series of elongate pores about 15 µm in width; strips covered by small granules. Although generally reticulate in texture, coenosteal strips parallel and straight on exsert abaxial side of each cyclosystem (Fig. 18A). In some specimens the coenosteum is porcellaneous (*polyorchis* form, Fig. 18I), whereas in others (typical form, Fig. 18G–H) it appears to be more porous. Coenosteum white, pale orange, and pale pink.

Cyclosystems linearly arranged on both edges of branches, as well as on anterior face, and occasionally on posterior face. Those on branch edges often closely spaced, sometimes directly adjacent to one another (Fig. 18C) or even coalescent. Cyclosystems circular, elliptical, or irregular in shape, 1.0–1.3 mm in diameter, slightly flared, and standing slightly exsert from branch; gastropores circular, 0.40–0.45 mm in diameter. Gastropore tubes cylindrical and usually slightly curved (Fig. 18M) on distal branches, such that gastrostyle tip is difficult to see; ring palisade often absent or only poorly developed. Gastrostyles lanceolate, about 0.5 mm in height, and occupying only lower quarter of gastropore tube; H:D about 2.3–2.5. Gastrostyle bears spines up to 0.4 mm in length.

Dactylotomes 0.10–0.11 mm in width; dactylostyles inconspicuous. Range of dactylopores per cyclosystem is 7–17 (n = 50, average = 11.94 (σ = 2.4), mode = 10). Supernumerary dactylopores absent. Pseudosepta slender and fairly uniform in width, 0. 11–0.13 mm in width, an adcauline diastema of about twice pseudoseptal width sometimes present.

Female ampullae (Fig. 18L) large, smooth, superficial hemispheres 1.0–1.1 mm in diameter, often having a lateral efferent pore about 0.25 mm in diameter. Male ampullae (Fig. 18A–B) superficial swellings 0.4–0.5 mm in diameter, often clustered on anterior face.

##### Remarks.

Fisher (1938: 505) discussed the similarities of *Stylaster campylecus* and *Stylaster polyorchis*, but concluded they were different species based on six minor differences: *Stylaster polyorchis* had smaller cyclosystems, straighter gastropore tubes, no ridges on the inside of the gastropore tube, often linked cyclosystems, a ring palisade, and wrinkled female ampullae. Based on more detailed SEM observations on many more specimens, these differences are considered to be intraspecific variation, the holotype of *Stylaster campylecus* itself having some linked cyclosystems and wrinkled female ampullae. The ring palisade is absent in most coralla, but in some specimens is weakly developed. *Stylaster campylecus tylotus* was also compared to but differentiated from typical *Stylaster campylecus* by Fisher (1938) in having a more delicate corallum, a larger ring palisade, a stouter gastrostyle, larger male ampullae, and more dactylopores per cyclosystem, but closer examinations also shows all these character to be within the range of variation of typical *Stylaster campylecus* (see Table 2). Likewise, no significant differences could be found between *Stylaster moseleyanus* and *Stylaster campylecus*, and thus these four taxa are considered to be the same. Interestingly, types of three of the four taxa were collected at *Albatross* station 3480, prompting Fisher (1938: 514) to remark about “the extraordinary number of species and subspecies dredged at station 3480,” considering the possibility that hybridism might be taking place.*Stylaster campylecus* and *Stylaster brochi* are the two most commonly collected stylasterids in the Aleutian Islands. *Stylaster campylecus* can be distinguished from other Alaskan species in *Stylaster* (Group B) by its slightly flared cyclosystems and the linearity of the coenosteal strips near the cyclosystems (Table 2). The corallum was found to be 100% aragonitic according to Cairns and Macintrye (1992). Of 94 colonies examined, 44 are female, 39 male, and 11 indeterminate, resulting in a fairly equal sex ratio.

##### Distribution.

Known from throughout the Aleutian Islands from Agattu Island to Unalaska, including Petrel and Bowers Banks, two disjunct records in Alexander Archipelago; 82-1011 m, but most records from 150-500 m.

#### Stylaster leptostylus

(Fisher, 1938) new rank

http://species-id.net/wiki/Stylaster_leptostylus

Figs 17E 19A–I

Allopora moseleyana forma *leptostyla* Fisher, 1938: 514, pl. 52, figs, 1–3.
Allopora moseleyana Fisher, 1938: in part (pl. 50).
Stylaster moseleyanus forma *leptostylus*.—Cairns 1983b: 429.

##### Type material.

Holotype: *Alb*-3480, 1 dry male colony 8.5 cm in height, and SEM stub 1544, USNM 43270 (Fig. 17E). Paratypes: *Alb*-3480, 4 female, 2 male colonies, dry, and SEM stub 1536–38, USNM 76817. **Type locality.** *Alb*-3480: 52°06'00"N, 171°45'00"W (off Seguam Island, Amukta Pass), 518 m.

##### Material examined.

Types. *Alb*-3480, 2 female, 1 male colonies, dry, USNM 76555 (paratype of *Allopora moseleyana*).

##### Description.

Corallum uniplanar, branching equal and dichotomous; no anastomosis of branches. Largest colony (the holotype, Fig. 17E) 8.5 cm in height and 7.0 cm in width, with a basal branch diameter of 8.4 mm. All branches circular in cross section. Commensal spionid polychaetes absent. Coenosteum reticulate-granular in texture, the coenosteal strips 45–55 µm in width, the slits about 15 µm in width; strips; strips not linear near cyclosystems. Coenosteum white.

**Figure 19.**
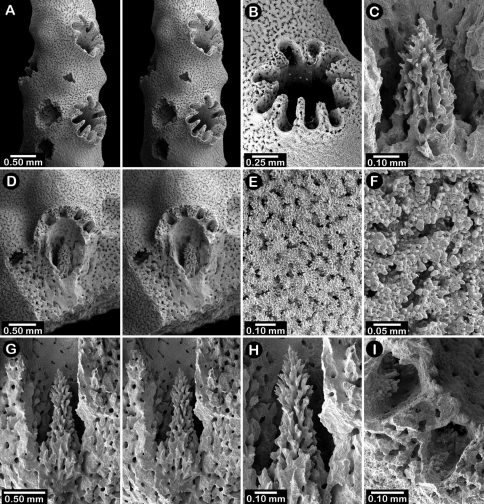
*Stylaster leptostylus*, **A–B, D–I** paratypes from USNM 76817, **C** holotype, USNM 43270: **A** stereo view of a male branch **B** a cyclosystem **C** gastrostyle **D** stereo view of a gastrostyle and female ampullae **E–F** coenosteal texture **G–H** gastrostyle **I** two dactylostyles.

Cyclosystems occur on branch edges and anterior face, but rarely on posterior face, those on edges not aligned in rows. Cyclosystems circular, 1.0–1.1 mm in diameter, and raised only slightly above branch coenosteum. Gastropores about 0.35 mm in diameter, leading to a slightly curved, funnel-shaped upper tube that narrows to a cylindrical lower part that houses the gastrostyle (Fig. 19G–H); ring palisade absent. Gastrostyles elongate-lanceolate, up to 0.6 mm in length, occupying lower half of gastropore tube, its pointed tip easily seen in an intact cyclosystem; H:D about 3.5.

Dactylotomes 0.10-0.13 mm wide; dactylostyles moderate in size, elements up to 50 µm in length. Range of dactylopores per cyclosystem 7–12 (n = 50, average = 9.76 (σ = 1.92), mode = 9). Supernumerary dactylopores absent. Pseudosepta slender and fairly uniform in width (0.10–0.11 mm), an adcauline diastema of twice that width sometimes present.

Female ampullae (Fig. 19D) large, smooth, superficial hemispheres 0.9-1.1 mm in diameter, occasionally having a lateral efferent pore about 0.20 mm in diameter. Male ampullae (Fig. 19A) superficial swellings 0.45-0.55 mm in diameter, often clustered on anterior and posterior branch faces; apical efferent pores about 25 µm in diameter.

##### Remarks.

Fisher (1938) originally differentiated forma *leptostyla* from typical *Allopora moseleyana* (=*Stylaster campylecus*) based on its more slender gastrostyle and smaller male ampullae. We cannot find a difference in ampulla size between forma *leptostyla* and typical *Stylaster campylecus*, but the gastrostyle of *leptostyla* is longer and more slender than that of *Stylaster campylecus*, occupying more of the gastropore tube (and thus having a higher H:D ratio) and being more visible from external view. In addition to this difference, forma *leptostyla* differs from *Stylaster campylecus* in having equal, non-anastomosing branching; branches circular in cross section; less exsert cyclosystems; non-linearly arranged cyclosystems; fewer dactylopores per cyclosystem; and consistently reticulate coenosteal strips (see Table 2). For these reasons this form is elevated to species rank but is considered to be closely related to *Stylaster campylecus*.

##### Distribution.

Known only from type locality.

#### Stylaster trachystomus

(Fisher, 1938) new rank

http://species-id.net/wiki/Stylaster_trachystomus

Figs 17H 20A–M

Allopora campyleca trachystoma Fisher, 1938: 510-511, pl. 45, fig. 2, pl. 46, pl. 54, figs 1–1b.—?Naumov 1960: 579.—Lowenstam 1964: 382 (mineralogy).
Stylaster campylecus trachystomus .—Cairns 1983b: 430.—Cairns and Macintrye 1992: 100-101 (mineralogy).—Wing and Barnard 2004: 27.—Heifetz et al. 2005: 134 (listed).—Stone and Shotwell 2007: 108 (listed).—Jameison et al. 2007: 224 (listed).

##### Type material.

Holotype: *Alb*-4784, 1 dry female colony 9 cm in height, and SEM stubs 1516, 1541, USNM 43265 (Fig. 17H). Paratypes: *Alb*-4784, 2 female, 2 male, 1 indet. colonies, and SEM stub 1517–18, dry, USNM 76811. **Type locality.** *Alb*-4784: 52°55'40"N, 173°26'E (off East Cape, Attu Island, Aleutians), 247 m.

##### Material examined.

Types; *Delta* 5999–8E-3, 51°21.042'N, 179°30.483'W, 115 m, 4 Jul 2003, 1 male, AB10–0001; *Delta* 6230–20–18, 52°28.142'N, 173°35.882'W, 190 m, 8 Jul 2004, 5 male branch fragments in alcohol, AB10–0004; *Shishaldin*, 54°54.09'N, 178°37.27'E, 366 m, 11 Feb 2000, 1 female, 2 male, AB00–45.

##### Description.

**Figure 20.**
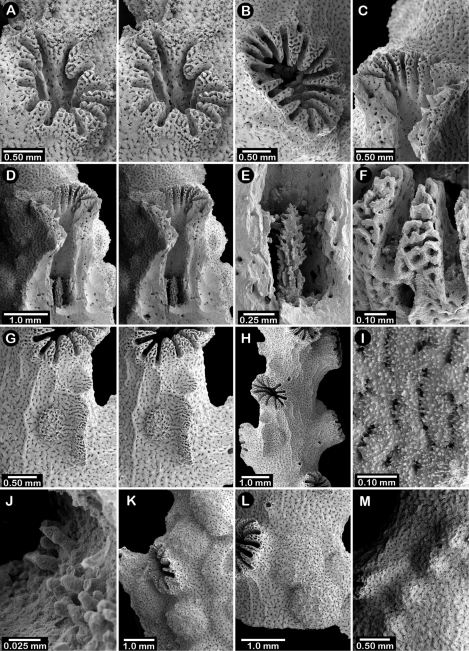
*Stylaster trachystomus*, **A–L** holotype, USNM 43265, **M** male paratype, USNM 76811: **A** stereo view of a cyclosystem showing internal decurrent ridges within gastropore **B** a cyclosystem **C** a damaged cyclosystem showing decurrent ridges **D** stereo view of longitudinal section of a cyclosystem and some female ampullae **E** a gastrostyle and ring palisade **F** porous pseudosepta **G** stereo view of ridged coenosteum and a small female ampulla **H** branch segment **I** coenosteal texture **J** rudimentary dactylostyle **K–L** clusters of female ampullae **M** male ampullae.

Corallum uniplanar, occasionally with short side branches oriented perpendicular to flabellum; branch anastomosis occurs only in large-diameter basal branches. Largest colony (paratype) 12 cm tall and 8 cm wide, with a basal branch diameter of 3 × 1.5 cm. Distal branches circular to slightly flattened in cross section. Commensal spionid worm tubes common. Coenosteum reticulate-granular in texture, the coenosteal strips 70–80 µm wide, the slits discontinuous and about 10-15 µm wide, the small granules producing a rough texture. On exsert cyclosystems, coenosteal strips are parallel and straight. Most coralla bear some short coenosteal ridges, called “spinous outgrowths” by Fisher (1938: 511), these thin ridges aligned with the branch (up to 1 mm long, 1 mm tall, and only 0.1 mm in width) or aligned with some of the pseudosepta on exterior wall of a cyclosystem (Fig. 20G–H). Coenosteum pale pink-orange.

Cyclosystems arranged uniformly on branches, not linearly, but fewer in number on posterior face. Cyclosystems rarely circular in shape, rather elliptical or asymmetrical, the longer axis of the ellipse up to 1.8 mm. Gastropore tubes cylindrical, slightly curved, and quite deep (up to 3 mm); a well-developed ring palisade present near gastrostyle tip, composed of squat elements about 50 µm in height and width. Gastrostyles lanceolate, up to 0.67 mm, having a H:D of about 3.1, and occupying lower quarter of gastropore tube. Because of curvature of the long gastropore tube, the short gastrostyle, and the wide pseudosepta, the gastrostyle tip is rarely seen when viewed from above.

Dactylotomes 0.085–0.10 mm in width, supernumerary dactylopores being quite rare, usually associated with pseudosepta. Dactylostyle inconspicuous (Fig. 20J). Range of dactylopores per cyclosystem 8–18 (n = 50, average = 11.82 (σ = 2.15), and mode = 11). Pseudosepta slender (0.07–0.21 mm wide), adcauline diastemas twice that width sometimes present; surface of pseudosepta quite porous (Fig. 20F).

Female ampullae (Fig. 20D, G, K–L) superficial hemispheres 1.0–1.3 mm in diameter, having a knobby or papillose surface; efferent pores not evident in material at hand. Male ampullae (Fig. 20M) also superficial swellings 0.50–0.55 mm in diameter. Both types of ampullae clustered on anterior and posterior branch faces.

##### Remarks.

Fisher (1938) described this taxon as a subspecies of *Stylaster campylecus*, distinguishing it from the nominate subspecies by having spongy pseudosepta (Fig. 20F), decurrent pseudoseptal ridges on the inside of the gastropore tube (Fig. 20C), and spiny coenosteal outgrowths (Fig. 20G–H). *Stylaster trachystomus* is otherwise similar to typical *Stylaster campylecus* in gastropore tube and gastrostyle shape and number of dactylopores per cyclosystem, but is also similar to *Stylaster brochi* in arrangement of cyclosystems and in hosting commensal spionid polychaetes; it also has unique characteristics as mentioned above (see also Table 2). Although few colonies are known of this species, because it does present a unique set of characteristics, it is considered valid and herein elevated to species rank. The corallum was found to be 100% aragonitic according to Cairns and Macintrye (1992).

##### Distribution.

Aleutian Islands: from off Attu Island and north of Amalia Island (Andreanof Islands), including Bowers Bank; 115–366 m.

#### Stylaster parageus parageus

(Fisher, 1938) new rank

http://species-id.net/wiki/Stylaster_parageus_parageus

Figs 21A 22A–J

Allopora campyleca paragea Fisher, 1938: 507-509, pl. 41, figs 1-1d, pl. 43.—Thompson and Chow 1955: 30 (mineralogy).
Stylaster (Allopora) boreopacificus forma *typica*.—Broch 1936: 56-60, in part: specimens from Alaska, pl. 8, fig. 22, pl. 9, figs 23, pl. 10, figs 24-25, text figs 17c-d.
Stylaster campylecus .—Lowenstam 1964: 382 (mineralogy).
Stylaster campylecus parageus .—Cairns 1983b: 430.—Cairns and Macintrye 1992: 100-101 (mineralogy).—Wing and Barnard 2004: 27 (listed), not fig. 32.—Heifetz et al. 2005: 134 (listed).—Stone and Shotwell 2007: 108 (listed).—Jameison et al. 2007: 224 (listed).

##### Type material.

**Figure 21.**
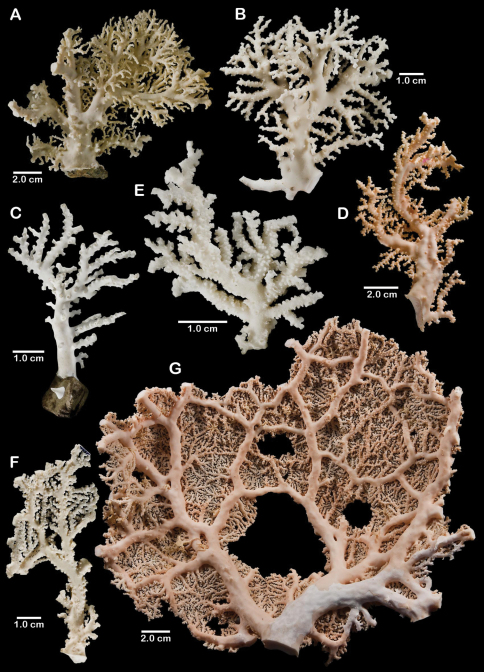
Color figures of skeleton of various Alaskan stylasterids: **A** *Stylaster parageus parageus*, **B** *Stylaster parageus columbiensis* **C** *Stylaster elassotomus* **D** *Stylaster crassiseptum* **E–G** *Stylaster alaskanus*. **A** holotype, USNM 42871 **B** holotype, USNM 1122462 **C** holotype, USNM 43268 **D** holotype, USNM 1122531 **E** holotype of *Stylaster gemmascens alaskanus*, USNM 43269 **F** holotype of *Stylaster cancellatus*, USNM 43267 **G** large colony, USNM 1122454.

Holotype: 1 dry male colony 13 cm in height, plus many tiny branch fragments, and SEM stub 1514, USNM 42871 (Fig. 21A). Paratypes: *Alb*-4245, 1 female colony, and SEM stub 1515, USNM 76812; near Sitka, Alaska, coll. E. R. Ricketts, 1 female, deposition unknown (not seen); Alaska, deposition unknown (not seen); Yakutat Bay, Alaska, 152 m, specimens reported by Broch (1936), Zoological Museum Copenhagen (not seen). **Type locality.** Tenakee Springs, near Juneau, Alaska, depth unknown.

##### Material examined.

Types; *Alaskan Leader* 21–91A, 59°31'18"N, 144°42'42"W, 401–600 m, 31 Jul 2002, 1 male, USNM 1122485; Lambert, coll., 55°18'N, 129°57'18"W, 23 m, 28 Mar 1976, 1 male in alcohol, USNM 76976; Freege and Worth, coll., 57°48'N, 134°02'W, 30 m, 17 Apr 2000, 4 branches in alcohol, USNM 1122523; “an Indian", coll., Sitka Bay, depth unknown, about 1884, 2 male branches, USNM 4192.

##### Description.

**Figure 22.**
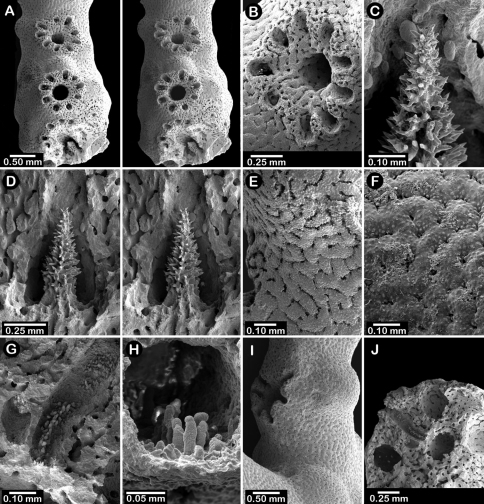
*Stylaster parageus parageus* **A–E, G–H, J** holotype (male), USNM 42871 **F** I female paratype, USNM 76812: **A** stereo view of three cyclosystems and male ampullae **B** a cyclosystem **C** gastrostyle tip surrounded by ring palisade **D** stereo view of a gastrostyle **E–F** coenosteal texture **G–H** rudimentary dactylostyles **I** female ampullae **J** cross section of internal male ampullae.

Corallum consisting of overlapping flabella, also with short branchlets oriented perpendicular to flabella, producing what Fisher (1938: 507) calls “subflabellate’ or a “flattened bush.” Branches do not anastomose, are circular to slightly flattened in cross section, and distally are quite delicate. Largest colony (the holotype, Fig. 21A) 13 cm tall and wide, with a basal branch diameter of 3.2 cm. Commensal spionid worm tubes common. Coenosteum reticulate-granular in texture (Fig. 22E–F), the coenosteal strips 60–65 µm in width, the slits only 6-8 µm wide, the strips covered with low rounded granules, altogether presenting a smooth dense aspect. Coenosteum white.

Cyclosystems occur on branch edges and anterior face, but rarely on posterior face. Cyclosystems circular in shape, small (0.9-1.0 mm in diameter, not 0.6 mm as stated by Fisher 1938), and only slightly elevated above coenosteum; gastropores circular and quite narrow (0.25–0.30 mm in diameter, Fig. 22B). Gastropore tubes cylindrical, slightly curved, such that gastrostyle tip rarely seen in view from above; well-developed ring palisade present (Fig. 22C), consisting of elongate elements oriented longitudinally, each about 45 µm in length and 30 µm in width. Gastrostyles lanceolate, up to 0.5 mm in height, and occupying the lower half of third of the gastropore tube; H:D about 2.7.

Dactylotomes 0.09–0.10 mm wide, the inner slit very shallow, resulting in a ring of essentially apical dactylopores surrounding a thick-walled gastropore tube (Fig. 22B). Dactylostyles well developed, the elements up to 35 µm in height and 10–11 µm in diameter (Fig. 22H). Range of dactylopores per cyclosystem 5–11 (n = 50, average = 8.54 (σ = 1.20), mode = 8). Supernumerary dactylopores present but not common. Pseudosepta 0.12–0.13 mm in width; diastemas rare.

Female ampullae (Fig. 22I) superficial hemispheres 0.8-1.0 mm in diameter, the lateral efferent pores being 0.15-0.20 mm in diameter. Male ampullae (Fig. 22A, J) partially submerged in coenosteum (internal) on distal branches, entirely internal on larger-diameter branches, the outer diameter being about 0.5 mm, the internal diameter of internal ampullae about 0.42 mm; male ampullae often clustered on branch faces.

##### Remarks.

Although Fisher (1938: 508) included this taxon as a subspecies of *Stylaster campylecus*, considering it to be “the southern shallow-water race of *campyleca,”* there are sufficient differences to warrant raising this subspecies to species rank (Table 2). Distinctive features include its bushy delicate colony, extremely small gastropores surrounded by a thick wall, and relatively low number of dactylopores per cyclosystem. Geographically it occurs only off southeastern Alaska in relatively shallow water, not in the Aleutian Islands. Comparisons to the other subspecies are made in the following account.

##### Distribution.

Bays and inland passages of southeastern Alaska from off Kayak Island to just north of Dixon Entrance (i.e., Prince of Wales Islands and Portland Canal); 23-401 m.

#### Stylaster parageus columbiensis

Lindner & Cairns ssp. n.

urn:lsid:zoobank.org:act:8B19F022-F84F-4AA7-B16B-E29DB8A8756D

http://species-id.net/wiki/Stylaster_parageus_columbiensis

Figs 21B 23A–J

Allopora campyleca Fisher, 1938: 506, in part (specimen from *Alb*-4230).

##### Type material.

Holotype: FOMC 1162 (209), male colony, dry, and SEM stubs 1389–91, USNM 1122462 (Fig. 22B). Paratypes: *Alaskan Leader* 21–108, 54°27'N, 133°55'48"W, 215–563 m, 10 Jul 2002, 1 male, USNM 1122483; *Alb*-4230, 55°N, 131°W, 198–439 m, 7 Jul 1903, 1 male, USNM 76815 (paratype of *Allopora campylecus*); FOMC 1158 (206), 48°07'546"N, 125°05'46"W, 246 m, 10 Jul 2008, 1 male in alcohol, USNM 1122465; FOMC 1159 (161), 48°07'56"N, 125°05'45"W, depth unknown, 10 Jul 2008, 3 males, USNM 1122472; FOMC (162), 48°07'56"N, 125°05'46"W, depth unknown, 10 Jul 2008, 1 male in alcohol, USNM 1122471; FOMC 1159 (166), 48°07'46"N, 125°05'41"W, 273 m, 10 Jul 2008, 1 male in alcohol, USNM 1122473; FOMC 1159 (169), 48°07'48"N, 125°05'41"W, 264 m, 10 Jul 2008, 1 male in alcohol, USNM 1122467; FOMC 1162 (203 and 205), 48°08'26"N, 125°11'28"W, 265 m, 12 Jul 2008, 2 males in alcohol, USNM 1122464, and -68; Lindner, coll., AL470 (41), Race Rocks, near Sooke Community, Straits of Juan de Fuca, British Columbia, depth unknown, Jul 2002, 1 male, USNM 1096625; Lindner, coll., AL466 (25), off Triangle Island, British Columbia, depth unknown, 1 male in alcohol, USNM 1096628; *Ropos* 956 (103), 48.15605°N, 124.9973°W, 288 m, 30 May 2006, 1 male, USNM 1117117; *Ropos* 957 (116), 48°08'42"N, 125°11'26"W, 285 m, 31 May 2006, 1 female in alcohol, SEM stub 1402, USNM 1117112. **Type locality.** 48°08'31"N, 125°11'01"W (off Cape Flattery, Washington), 273 m.

##### Etymology.

Named for the region from which it is primarily known: British Columbia.

##### Material examined.

Types.

##### Description.

**Figure 23.**
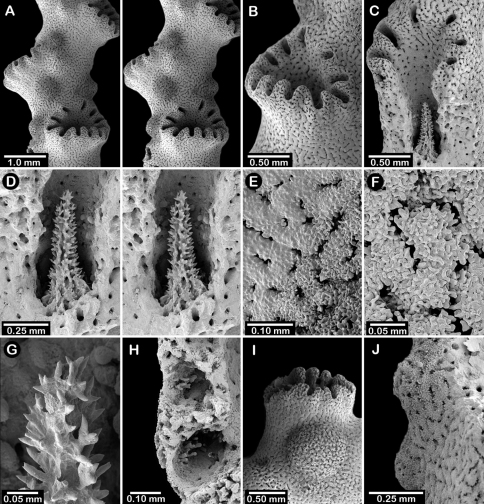
*Stylaster parageus columbiensis*, **A–H, J** holotype (male), USNM 1122462, **I** female paratype, USNM 1117112: **A** stereo view of branch segment including male ampullae **B** a cyclosystem **C** longitudinal fracture of a cyclosystem **D** stereo view of a gastrostyle and rudimentary ring palisade **E–F** rough and smooth coenosteal texture **G** gastrostyle tip and ring palisade **H** two dactylostyles **I** female ampulla **J** male ampullae.

Corallum shape and branching similar to that of typical subspecies: bushy or flattened bushy with delicate terminal branches. Holotype 7 cm tall and 7 cm wide, with a basal branch diameter of 10.1 × 8.1 mm; largest corallum (USNM 1096625, Fig. 21B) 8.5 × 8.0 cm, with a massive basal branch diameter of 3 cm. Commensal spionid worm tubes absent. Coenosteum reticulate-granular in texture, the coenosteal strips 50–60 µm wide, bordered by slits about 10 µm wide, the strips originally covered with irregularly-shaped nodules that are subsequently covered with smoother granular coenosteum (Fig. 23E shows the transition). Coenosteum white.

Cyclosystems occur exclusively on edges of distal branches, but also on anterior face of larger-diameter branches. Cyclosystems circular to elliptical in shape, the larger axis ranging from 1.1 to 1.5 mm, the abaxial edge slightly raised above coenosteum; gastropores circular, 0.45–0.50 mm in diameter. Gastropore tubes somewhat inflated in upper half, changing to a narrow cylinder proximally; a well-developed ring palisade present as in the typical subspecies, the narrow pointed gastrostyle tip projecting through the ring palisade constriction into upper chamber (Fig. 23G) and thus easily visible from above. Gastrostyles elongate-lanceolate (Fig. 23D), occupying lower half of gastropore tube, and up to 0.67 mm in length, having a H:D ratio of 3.1–3.7.

Dactylotomes 0.09–0.10 mm in width, the inner slit short but longer than that of typical subspecies. Dactylostyles well developed, the cylindrical elements up to 38 µm in height and about 14 µm in diameter. Range of dactylopores per cyclosystem 6–13 (n = 50, average = 9.38 (σ = 1.43), and mode = 9). Supernumerary dactylopores rare. Pseudosepta 0.17–0.28 mm in width; diastemas uncommon.

Female ampullae (Fig. 23I) smooth superficial hemispheres 0.9–1.0 mm in diameter, with lateral efferent pores about 0.23 mm in diameter. Male ampullae (Fig. 23J) superficial swellings up to 0.5 mm in diameter, with small apical efferent pores. Both types of ampullae clustered on anterior and posterior faces.

##### Remarks.

Subspecies *columbiensis* resembles the typical subspecies in many ways, including colony shape and branching, and ring palisade shape, but also differs in a number of small but consistent characteristics (Table 2). Subspecies *columbiensis* has larger cyclosystems and gastropores, a slightly higher average number of dactylopores per cyclosystem, a more commodious upper gastropore chamber and deeper dactylotome slits, and a more elongate gastrostyle that is easily seen from above. Dixon Entrance appears to be the border between the two subspecies, the typical subspecies occurring to the north and *columbiensis* occurring to the south of this body of water. Because of the overall similarity of the two taxa, and their consistent minor differences, including their geographic separation, *columbiensis* is considered as a subspecies or *Stylaster parageus*. Of 16 colonies examined, 15 are male and one is female.

##### Distribution.

Entire coast of British Columbia (Canada) from Dixon Entrance to off Cape Flattery, Washington (USA); 246–285 m.

### Stylaster (Group C)

**Diagnosis.** Species of *Stylaster* in which coralla form branching colonies, and in which cyclosystems are arranged exclusively on branch edges; branching delicate.

#### Stylaster alaskanus

Fisher, 1938

http://species-id.net/wiki/Stylaster_alaskanus

Figs 21E–G 24A–M

Stylaster gemmascens alaskanus Fisher, 1938: 500–502, pl. 47–48, pl. 54, fig. 2.—Boschma 1957: 11.
Stylaster cancellatus Fisher, 1938: 502–503, pl. 35, figs 2–2c, pl. 39–40.—Boschma 1957: 4.—Cairns 1983b: 430 (listed).—Heifetz 2002: 22 (listed).—Wing and Barnard 2004: 11, 27, fig. 33.—Heifetz et al. 2005: 22, 134, 137 (listed).—Stone and Shotwell 2007: 108 (listed).—Brooke and Stone 2007: 525–530, fig. 2D, 3B.—Jameison et al. 2007: 224 (listed).—Lindner et al. 2008: 3, supplemental Table S1: 2 (phylogeny and DNA sequence).
Stylaster alaskanus : Cairns 1983b: 430 (elevation to species rank).—Wing and Barnard 2004: 11, 27.—Stone and Shotwell 2007: 108 (listed).
Stylaster alaskana : Heifetz et al. 2005: 133 (listed).—Jameison et al. 2007: 224 (listed but misspelled).

##### Type material.

*Stylaster gemmascens alaskanus*: Holotype, *Alb*-3480, 1 dry male branch fragment 4.5 cm in length, USNM 43269 (Fig. 21E); 5 (perhaps one broken from the four mentioned by Fisher) dry paratype branches (2 female, 2 male, 1 indet.), and SEM stubs 1511–12, *Alb*-3480, USNM 86006. **Type locality.** *Alb*-3480: 52°06'N, 171°45'W (Amukta Pass, Aleutian Islands), 518 m.

*Stylaster cancellatus*: Holotype, *Alb*-3480, 1 dry female branch fragment 9 cm in height, and SEM stub 1509, USNM 43267 (Fig. 21F); 7 dry paratype branches (2 female, 5 male), and SEM stub 1510, *Alb*-3480, USNM 86005. **Type locality.** *Alb*-3480: 52°06'N, 171°45'W (Amukta Pass, Aleutian Islands), 518 m.

##### Material examined.

Types of *Stylaster alaskanus* and *Stylaster cancellatus*; *Alaskan Leader* 35, 51°50.9'N, 175°08'W, 178–365 m, 14 Jun 2002, 1 male, AB02–31;*MF* 833–38, 51°42'54"N, 178°51'30"W, 582 m, 3 Aug 1983, 2 female, USNM 96526; *MF* 833–56, off Great Sitkin I., 249 m, 6 Aug 1983, 2 male, USNM 77045; *Alaska Sea*, 52°09'24"N, 173°44'19"E, 318 m, 5 Feb 2000, 1 indet., USNM 1122566; *Alb*-3480, see above, 8 female and 1 male (non-type), USNM 76722; *Dominator* 971–135, 51°37'22"N, 178°26'28"W, 163 m, 14 Jul 1997, 2 female, USNM 1123365; *Dominator* 971–201, 51°54'28"N, 175°49'00"E, 378 m, 1 Aug 1997, 1 indet., USNM 1123361; *Ocean Olympic*, 52°04.78'N, 177°13.39'E, 256 m, 1 female, AB00–57; *Pacific Knight* 941–204, 53°06'N, 171°42'E, 455 m, 31 Jul 1994, 4 female some in alcohol, USNM 96246 and 1123533; *Sea Storm* 36, 53°05'45"N, 171°41'56"E, 453–458 m, 19 Jun 2002, 1 female, 1 male, USNM 1123277–78; *Sea Storm* 108, 52°11'32"N, 175°16'54"W, 208–215 m, 8 Jul 2002, 1 indet. in alcohol, USNM 1027822; *Sea Storm* 138, 52°13'50"N, 175°24.2'E, 146 m, 16 Jul 2002, 2 female, 2 male, USNM 1122766–68, 1122898; *Sea Storm* 148, 52°28'16"N, 173°29'35"W, 194 m, 21 Jul 2002, 2 female, USNM 1122757; *Shishaldin*, 54°24'04"N, 177°37'43"E, 154 m, 9 Feb 2000, 1 female, USNM 1122533; *Shishaldin*, 54°24'03"N, 179°36'11"E, 313 m, 25 Feb 2000, 4 indet., USNM 1122532; McClusky, coll., 52°01'47"N, 179°56'14"W, 362 m, 5 May 2000, 1 female, USNM 1122454; McClusky, coll., 51°17'58"N, 179°00'48"E, 265 m, 25 May 2000, 3 male, USNM 1122455 and AB01–92; University of Washington, 51°32'N, 179°15'W, 278–289 m, 1 Sep 1968, 4 male, USNM 76632 and 96530; A. Vatter, coll., 52°04'25"N, 177°11'00"E, depth unknown, 1 male, USNM 1122466.

##### Description.

**Figure 24.**
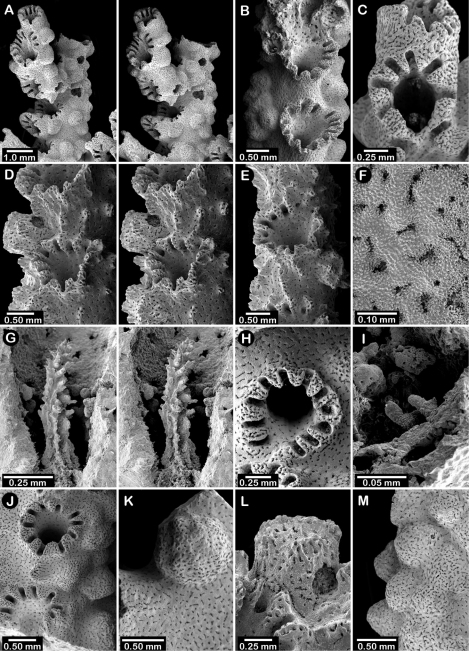
*Stylaster alaskanus*, **A–C, M** male paratype of *Stylaster cancellatus*, USNM 86005 **D–E, H, J, L** female paratype of *Stylaster gemmascens alaskanus*, USNM 86006, **F–G, I, K** holotype (female) of *Stylaster cancellatus*, USNM 43267: **A** stereo view of branch tip with male ampullae **B–C, J** cyclosystems and male ampullae **D** stereo view of cyclosystem and female ampulla with a distinct efferent pore **E** cyclosystem and female ampulla **F** coenosteal texture **G** stereo view of gastrostyle and ring palisade **H** a cyclosystem **I** rudimentary dactylostyle **K–L** female ampullae **M** cluster of male ampullae.

Colonies uniplanar or multiplanar, additional flabella usually oriented perpendicular to original flabellum. Branches highly anastomotic, smooth large-diameter branches forming a framework for infilling by smaller-diameter branches, resulting is a sieve-like reticulum (Fig. 21G). Largest colony (USNM 1122768) 28 cm in height, having a massive, dense basal branch diameter of 5 cm. Distal branches circular to rectangular in cross section. Spionid worm tubes do not occur in this species. Coenosteum reticulate-granular in texture, the coenosteal strips 50–60 µm wide, bordered by short slits 6–8 µm wide; strips covered with small granules 6–7 µm in width; strips arranged linearly adjacent to cyclosystems. In some colonies, such as the type of *Stylaster alaskanus*, linearly arranged, apically perforate processes up to 0.2 mm tall (Figs 24D–E, J) occur on coenosteum, these structures of unknown function. Coenosteum light orange, pink, or white.

Cyclosystems circular, elliptical or irregular in shape, 0.9–1.3 mm in greater diameter, occurring exclusively on branch edges; gastropores circular, 0.40–0.45 mm in diameter. Gastropore tubes straight and funnel-shaped, having a well-developed ring palisade (Fig. 24G) composed of rounded elements about 40 µm in diameter. Gastrostyles slender and pointed, up to 0.5 mm in height, the gastrostyle tip easily seen when viewed from above. Very rarely two gastrostyles may be present in one gastropore tube.

Dactylotomes 80–85 µm in width; dactylostyles inconspicuous. Range of dactylopores per cyclosystem 7–14 (n = 50, average = 11.30 (σ = 1.57), and mode = 13). Supernumerary dactylopores absent. Pseudosepta of cyclosystems usually only slightly exsert, but if two cyclosystems are in close proximity, pseudosepta may become quite exsert. Pseudosepta of uniform thickness; diastemas, if present at all, are narrow.

Female ampullae (Fig. 24D–E, K–L) prominent hemispheres up to 0.9 mm in diameter, often bearing several low ridges or short spines; lateral efferent pore about 0.25 mm in diameter. Male ampullae (Fig. 24A–B, J, M) superficial, densely clustered, and also irregular in shape, up to 0.60 mm in diameter, each with a small efferent pore 40–60 µm in diameter. Efferent pores sometimes at the apex of a small curved spine.

##### Remarks.

Originally described as a subspecies of *Stylaster gemmascens* by Fisher (1938), Cairns (1983b) later raised *alaskanus* to species status. Herein it is synonymized with *Stylaster cancellatus*, but since it has page priority in Fisher (1938), *Stylaster alaskanus* is considered to be the senior synonym (see synonymy above). The types of *Stylaster alaskanus* differ from most other specimens assigned to this species in having numerous coenosteal papillae, called “thorny outgrowths” by Fisher (1938: 501), which are often aligned in short ridges. Also, the coenosteum is white and the branches of the type series do not anastomose. However, all these differences are considered to be intraspecific variation, as some other specimens assigned to this species have a white corallum, and small colonies, like the types of *Stylaster alaskanus*, usually do not have anastomosing branches. Furthermore, rarely some coralla of “*cancellatus*” have coenosteal papillae, which may be a reaction to an unfavorable environmental habitat. Thus, the type series of *Stylaster alaskanus*, which was collected at the same station as the type of *Stylaster cancellatus*, is considered to be a somewhat aberrant specimen of what was more widely known as *Stylaster cancellatus*. A similarly shaped, sieve-like, reticulate colony is known for *Errinopsis reticulum* Broch, 1951, however, in that species the reticulum is composed of equally-sized branches, whereas in *Stylaster alaskanus* the reticulum is composed of thick and thin branches. *Stylaster alaskanus* is distinguished from all other species in its group by having a corallum shaped as a sieve-like reticulum. It is further distinguished from those Alaskan species of group C by having ridged female ampullae (Table 2). Among the 59 specimens examined, 25 are female, 22 male, and 12 indeterminate, a fairly even sex ratio.

##### Distribution.

Known from throughout Aleutian Islands from west of Attu Island to Amukta Pass, including Bowers Bank; 146-582 m.

#### Stylaster elassotomus

Fisher, 1938

http://species-id.net/wiki/Stylaster_elassotomus

Figs 21C 25A–G

Stylaster elassotomus Fisher, 1938: 499-500, pl. 41, fig. 3, pl. 42, figs 1–1c, pl. 49, fig. 1.—Boschma 1957: 7.—Wing and Barnard 2004: 11 (key), 28.—Heifetz et al. 2005: 134, 137 (listed).—Stone and Shotwell 2007: 108 (listed).—Jameison et al. 2007: 224 (listed).

##### Type material.

Holotype: 1 dry male colony, and SEM stub 1507, USNM 43268 (Fig. 21C). The pale pink paratype 5 cm in length (USNM 86007) mentioned by Fisher is an unidentified *Stylaster* having little in common with the holotype. **Type locality.** *Albatross* 4781, 52°14'30"N, 174°13'E (off Agattu Island), 882 m.

##### Material examined.

Types.

**Description.** Holotype (Fig. 21C) colony bushy, 5.5 cm tall and 4.5 cm wide, attached by a basal branch 6 mm in diameter. Branches do not anastomose and are circular in cross section. Coenosteum reticular-granular in texture, the strips 55–60 µm in width, separated by slits 10–14 µm wide; strips covered with small angular granules. Spionid worm tubes absent. Coenosteum white.

**Figure 25.**
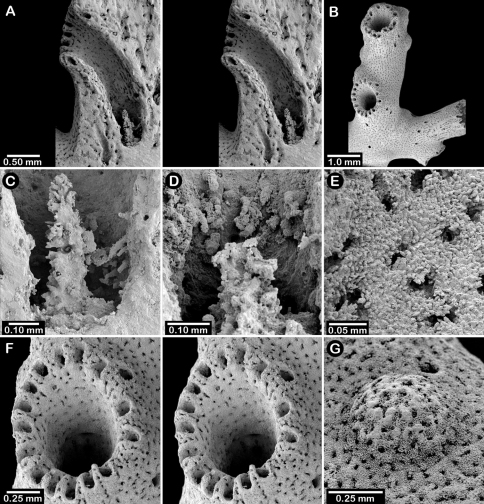
Holotype (male) of *Stylaster elassotomus*, USNM 43268: **A** stereo view of curved gastropore tube **B** cyclosystems and supernumerary dactylopores **C** poorly preserved gastrostyle and ring palisade **D** truncated gastrostyle showing ring palisade **E** coenosteal texture **F** stereo view of cyclosystem showing shallow dactylotomes **G** male ampulla.

Cyclosystems circular to slightly elliptical, 1.0–1.2 mm in diameter, arranged exclusively on branch edges in an alternating fashion, most projecting perpendicular to branch. Gastropores circular and quite large (0.5–0.6 mm in diameter), occupying up to 55% of cyclosystem diameter (Fig. 25F). Gastropore tube cylindrical, curved, and long, such that gastrostyle tip is rarely seen when viewed from above (Fig. 25A). A delicate ring palisade (Fig. 25C–D) occurs near gastrostyle tip, composed of slender cylindrical elements up to 60 µm long and 25 µm in diameter. Gastrostyle slender (H:D = 4.4), the illustrated style 0.53 mm in height, occupying lower quarter of gastropore tube (Fig. 25A).

Dactylotomes 60–80 µm wide but very short as well as having short internal slits, thus contributing to the large size of the gastropore tube (Fig. 25F); dactylostyles inconspicuous. Range of dactylopores per cyclosystem 11–17 (n = 21, average = 14.40 (σ = 1.36), and mode = 15). Supernumerary dactylopores common (Fig. 25B), 70–100 µm in diameter. Pseudosepta same width as dactylotomes; diastemas rare.

Female ampullae unknown. Male ampullae (Fig. 25G) hemispherical, 0.5–0.6 mm in diameter.

##### Remarks.

Despite access to a diverse stylasterid collection from the Aleutian Islands, no unequivocal additional specimens could be identified as *Stylaster elassotomus*, even the paratype of the species not considered to be conspecific. Its distinctive characteristics of having a highly curved gastropore tube, slender ring palisade elements, and a very large gastropore surrounded by very short dactylotomes and pseudosepta distinguish it from all other Alaskan species (see Table 2).

##### Distribution.

Known only from type locality.

#### Stylaster crassiseptum

Lindner & Cairns sp. n.

urn:lsid:zoobank.org:act:D638F3B7-D697-40BE-B502-5D4F5F9E519B

http://species-id.net/wiki/Stylaster_crassiseptum

Figs 21D 26A–J

##### Type material.

Holotype: dry female branch fragment 12 cm in length, plus many smaller broken pieces, and SEM stubs 1520–1521, 1546, USNM 1122531 (Fig. 21D). Paratypes: *North Pacific*, 52°04'17"N, 176°59'21"E , 366 m, date unknown, 1 female, USNM 1122511; *Shishaldin*, 54°00'41"N, 179°46'52"E, 291 m, 14 Mar 2000, 1 female, USNM 1122497; *Shishaldin*, 53°56'24"N, 179°49'31"E, 318 m, 14 Mar 2000, 1 male, and SEM stub 1522–23, USNM 1122519; *Shishaldin*, 51°48.49'N, 174°29.92'W, 531m, 2000, 1 female, USNM 112527. **Type locality.** *Shishaldin* station, 53°59'50"N, 179°46'52"E (off Bowers Bank, Aleutian Islands), 291 m, 5 Mar 2000.

##### Etymology.

The specific name *crassiseptum* (from the Latin *crassus*, meaning “thick" + *septum* meaning “wall"), in reference to the wide pseudosepta of the cyclosystems.

##### Material examined.

Types.

##### Description.

Colonies primarily uniplanar, having no branch anastomosis. Holotype (Fig. 21D) a branch fragment 12 cm in length and 1 cm in basal branch diameter; largest specimen (USNM 1122527) an intact colony 24 cm in height and 19 cm wide, with a basal branch diameter of 4.5 cm. Distal branches circular in cross section, basal branches somewhat rectangular in cross section, the longer axis perpendicular to colony plane; symbiotic polychaetes absent. Coenosteum reticulate-granular in texture, coenosteal strips 60–70 µm in width, separated by very narrow slits only 3–5 µm wide; coenosteal strips not linearly arranged near cyclosystems. Coenosteal granules very low and smooth, conferring a shiny or porcellaneous texture to branches. Coenosteum dense and uniformly pale orange.

**Figure 26.**
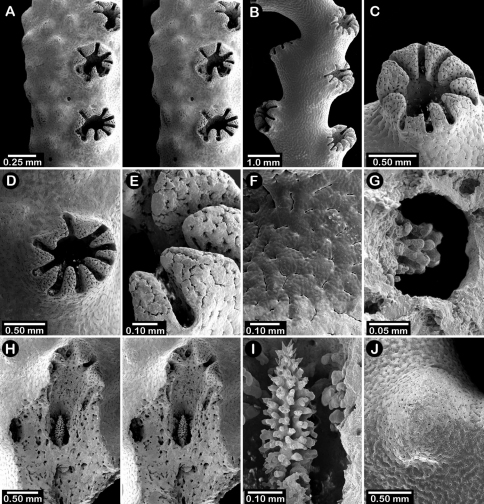
*Stylaster crassiseptum* **A, D, H–I** male paratype, USNM 112519, **B–C, E–G, J** holotype (female), USNM 1122531: **A** stereo view of cyclosystems and a cluster of male ampullae **B** branch segment showing sympodial arrangement of cyclosystems **C–D** cyclosystems **E** thick pseudosepta and thin dactylotomes **F** coenosteal texture **G** robust dactylostyle **H** stereo view of gastropore tube and male ampulla **I** gastrostyle surrounded by well–developed ring palisade **J** female ampulla.

Cyclosystems circular, slightly exsert, and relatively small (0.7–1.0 mm in diameter), occurring exclusively on branch edges (Fig. 26A) in alternating fashion (Fig. 26B), most projecting perpendicular to branch. Cyclosystems well spaced, separated by 1–3 cyclosystem diameters from one another on one side of a branch. Gastropores circular, about 0.5 mm in diameter; gastropore tubes cylindrical and slightly curved, having a well-developed ring palisade (Fig. 26I) composed of numerous large squat elements up to 35 µm in height and 50 µm in diameter. Gastrostyles lanceolate and slender, the figured style being 0.48 mm in height and only 0.15 mm in diameter (Fig. 26I), the tip usually seen when viewed from above. Gastrostyle covered with blunt spines up to 33 µm in length.

Dactylotomes slender and uniform in width (60–70 µm); dactylostyles quite robust (Fig. 26G), the large cylindrical elements (up to 45um tall and 13 µm in diameter) almost completely filling the lower dactylopore, but because of the narrow dactylopores and exsert pseudosepta, the dactylostyles are barely visible in an intact cyclosystem. Range of dactylopores per cyclosystem 6–12 (n=60, average = 8.89 (σ = 1.4), mode = 9). Supernumerary dactylopores not present. Pseudosepta solid, exsert, and variable in width (0.15–0.31 mm in width), or up to five times width of adjacent dactylotomes (Fig. 26C–E). Diastemas rare.

Female ampullae (Fig. 26J) smooth superficial hemispheres 0.9–1.1 mm in diameter, with a lateral efferent pore about 0.3 mm in diameter. Male ampullae (Fig. 26A, H) often clustered, slightly irregular in shape, 0.45–0.50 mm in diameter, often with one or more tiny (28–30 µm in diameter) apical efferent pores.

##### Remarks.

Although similar to *Stylaster alaskanus*, *Stylaster crassiseptum* is distinguished by having non-anastomosing branches, a curved gastropore tube, relatively wide pseudosepta, and more robust dactylostyles (Table 2).

##### Distribution.

Aleutian Islands from off Kiska to off Atka Islands, Bowers Bank; 291–531 m.

### Genus Stylantheca

Fisher, 1931

http://species-id.net/wiki/Stylantheca

#### Diagnosis.

Colonies encrusting, forming thin laminae on rocks and shells, rarely forming short stubby branches. Coenosteum reticulate-granular and of many colors; coenosteal papillae quite common. Gastro- and dactylopores arranged in cyclosystems, often with more than one gastrostyle per cyclosystem; pores of the peripheral type; supernumerary dactylopores rare. Gastropore tube single-chambered, but may be constricted by a ring palisade. Dactylostyles usually robust. Ampullae usually internal.

#### Discussion.

As summarized by Cairns (1983b), species attributed to this genus have been placed in the genera *Allopora*, *Stylaster*, and as a subgenus of *Stylaster*. *Stylantheca papillosa* (Dall, 1884) is indeed very similar to *Stylaster* (Group A, i.e., *Allopora*). At present the only characters differentiating *Stylantheca* from *Stylaster* (Group A) is its tendency to form encrusting colonies, and its tendency to have more than one gastrostyle per cyclosystem, although there are exceptions to both of those criteria. Deeper water colonies of *Stylantheca papillosa* often have short stubby branches, and the northern range of *Stylantheca papillosa* sometimes has only one gastrostyle per cyclosystem (see Remarks of *Stylantheca papillosa*). It is likely that *Stylantheca* belongs to the *Stylaster* genus, perhaps as a fifth group, i.e., “Group E”, of species sensu Cairns (1983b) that would include encrusting species with a tendency to have multiple gastrostyles per cyclosystems. But until more specimens have been collected to study morphological variation, we choose to retain the genus *Stylantheca*.

#### Type species.

*Stylantheca porphyra* Fisher, 1931, by monotypy.

#### Distribution.

From the Alaska Peninsula to Lower California, 0–27 m.

### Stylantheca papillosa

(Dall, 1884)

http://species-id.net/wiki/Stylantheca_papillosa

Figs 27D–G 29A–L

**Figure 27.**
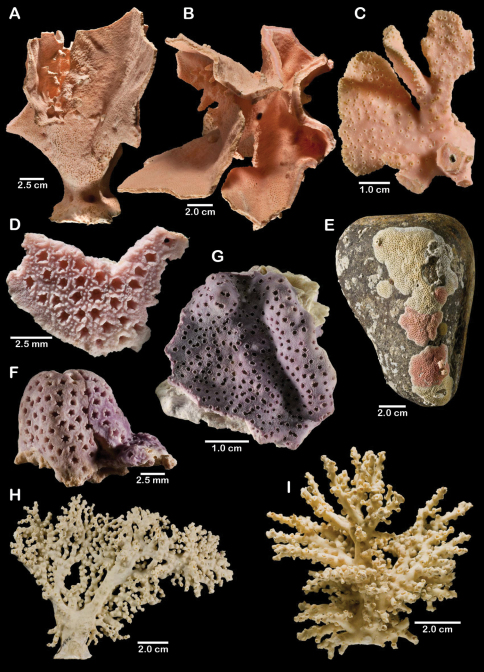
Color figures of skeleton of various Alaskan stylasterids: **A–C** *Stylaster repandus* **D–G** *Stylantheca papillosa* **H–I** *Crypthelia trophostega*: **A–B** lateral and apical views of holotype, USNM 1122740 **C** small paratype colony, USNM 1122741 **D** holotype of *Allopora papillosa*, USNM 6852 **E** encrusting colony, AB02–0005 **F** syntype of *Allopora petrograpta*, USNM 43272 **G** holotype of *Stylaster porphyrea*, USNM 43018 **H** holotype, USNM 42876 **I** arborescent colony, USNM 1122435.

Allopora papillosa Dall, 1884: 113-114.—Fisher 1938: 527–528, pl. 54, fig. 4, pl. 59, fig. 3.
Stylantheca porphyra Fisher, 1931: 395–397, pl. 15, figs 1, 1a, pl. 16, figs 5, 5a-b, pl. 17, figs 6, 6a-c.—Boschma 1956: F100, text fig. 81–1a-b; 1960: 426–427, text figs 1e-g.—Cairns 1983b: 430, 481–483, figs 18A-I, 24H, 27G, J.—Wing and Barnard 2004: 10, 27.—Heifetz et al. 2005: 133 (listed).—Stone and Shotwell 2007: 108 (listed).—Whitmire and Clarke 2007: 154 (listed).—Jamieson et al. 2007: 224 (listed).
Allopora petrograpta Fisher, 1938: 530–531, pl. 54, figs 5, 5a, pl. 59, fig. 4.—Frichman 1974: 245–258 (larval development).
Allopora porphyra : Fisher 1938: 528–530, pl. 59, figs 1–2, pl. 60, pl. 61,figs 1, 1a, pl. 70, figs 2, 2a.—Thompson and Chow 1955: 30 (mineralogy).
Stylaster (Allopora) porphyrus : Broch 1942: 102.
Stylaster (Stylantheca) porphyra : Boschma 1951: 39, text fig. 5b.
Stylantheca petrograpta : Cairns 1983b: 430.—Wing and Barnard 2004: 10, 27.—Heifetz et al. 2005: 133 (listed).—Stone and Shotwell 2007: 108 (listed).—Whitmire and Clarke 2007: 154 (listed).—Jamieson et al. 2007: 224 (listed).—Lindner et al. 2008: 3, and supplemental Table 1: 2 (phylogeny and DNA sequences).
Stylantheca papillosa : Cairns 1983b: 430.—Wing and Barnard 2004: 10, 27.—Heifetz et al. 2005: 133 (listed).—Stone and Shotwell 2007: 108 (listed).—Jamieson et al. 2007: 224 (listed).
Stylaster porphyra : Jamieson et al. 2007: 224 (listed).

#### Type material.

*Allopora papillosa*: holotype, a small (11 mm long) dry encrusting fragment, USNM 6852 (Fig. 27D).

#### Type locality.

Coal Harbor, Unga Island, Shumagin Islands, Alaska Peninsula, 11 m.

*Stylantheca porphyrea*: holotype (USNM 43018, Fig. 27G) and 13 paratype colonies (USNM 43019, 43276, 43277 and SEM stub 136), all dry.

#### Type locality.

Pescadero Point, Carmel Bay, California (36°33'42"N, 121°57'13"W), intertidal.

*Allopora petrograpta*: two small (15 and 17 mm in length) dry, male encrusting fragments, syntypes, and SEM stub 1506, USNM 43272 (Fig. 27F). **Type locality.** Kyack Island, mouth of Sitka Harbor, Alaska, intertidal.

#### Material examined.

Types of the three named species; Middle of Cross Sound, AK, 15–21 m, 27 Jul 1978, 5 female colonies, AB78–120; South of Yasha Island, Chatham Strait, AK, depth unknown, 31 Jul 1976, 8 female colonies in alcohol, and SEM stubs 1532–34, AB76–55; Wooden Island, AK, depth unknown, 2002, 1 indet., AB02–150; Wooden Island, AK, depth unknown, 15 May 2009, 1 male in alcohol, AB09–10; Race Rocks, Victoria, British Columbia, depth unknown, 2002, 8 colonies dry and in alcohol, USNM 1073478; Seontary Island, Victoria, BC, 10 m, Jul 2002, 1 large male colony, USNM 1096626; 48°18'N, 123°31.8'W, 12.1 m, 6 Sep 1973, 1 female colony, USNM 76559; cactus island Channel, BC, 15 m, 1 male, AB02–0006; Lion's Gate Bridge, Burrard Inlet, British Columbia, 18 m, Jan 2002, 2 indet., AB02–0005; Turn Island, Washington, 0–27 m, 6 Jul 1995, 10 colonies, USNM 1084659; Puget Sound, Washington, 27 m, 2, CAS 117458–59; Squaw Island, Oregon, 20 Jul 1962, 1 female colony, USNM 45685; 43°18'15"N, 124°23'56"W (Charleston, Oregon), intertidal, 2 indet in alcohol, USNM 1086321; Pillar Point, Half Moon Bay, California, intertidal, 18 Feb 1996, 16 colonies, USNM 1084663; Pigeon Point, California, intertidal, 12 Jul 1995, 12 colonies (forma *porphyra*), USNM 1084662.

#### Description.

The typical form is variable in colony shape, those specimens living in a high energy shallow-water environment usually being encrusting, the layer of coenosteum sometimes less than 1 mm thick (Fig. 27E), the gastrostyle base almost resting on the hard substrate. In deeper water, colonies produce short (up to 17 mm), knobby, clavate, cylindrical branches, which originate from the basal encrustation. Only rarely will these branches bifurcate. Although the types of *Stylantheca papillosa* and *Stylantheca petrograpta* are small, beach-worn fragments (Figs 27D, F), the species produces mats up to 30 cm in diameter. Parasitic spionid polychaetes (*Polydora* ?*alloporis* Light, 1970) usually present, forming binary tubes/paired burrows throughout the coenosteum. Coenosteum reticulate-granular in texture, the strips about 50–55 µm in width, separate by slits 12–15 µm wide. Short, porous, conical papillae (nematopores?) common on coenosteum, each about 0.13 mm in diameter and equally tall (Fig. 29B, D, F–G). Coenosteum purple, pink, red, and occasionally white, the tips of the clavate branches usually white.

Cyclosystems uniformly arranged on encrustations and on all sides of branchlets; diameter of cyclosystems 0.9–1.2 mm. Gastropore tube highly constricted, the upper larger portion being infundibuliform to spherical, the lower chamber spherical and almost completely occupied by the gastrostyle(s), the two portions of the chamber constricted near gastrostyle tip. Gastropore tube diameter about 0.3 mm, the tube constriction about half that diameter. Just above the constriction numerous small (up to 45 µm in length) papillae form a wide ring palisade (Fig. 29K) further reducing access to lower chamber. Gastrostyles quite variable and sometimes irregular in shape, including globose (Fig. 29K), lanceolate, or triangular. Regardless, gastrostyles bear quite long (up to 50 µm long and 15 µm in diameter) spines that completely obscure the underlying gastrostyle shaft. Usually there is only one gastrostyle per cyclosystem, but occasionally the upper chambers of two cyclosystems are linked together resulting in two gastrostyles in one cavity, and rarely there appears to be 2 gastrostyles in one normal gastropore chamber.

In well-preserved colonies, dactylopores are slightly raised above surface of coenosteum; dactylotomes 0.10–0.12 mm wide. Number of dactylotomes per cyclosystem variable, depending on size of cyclosystem, but ranges from 2–12, with common modes of 4, 5 and 8 (see Remarks). Dactylostyles robust (Fig. 29A, C, H), the cylindrical elements up to 55 µm long and 13–17 µm in diameter, not unlike the gastrostyle elements.

Female ampullae (Fig. 29L) primarily internal, with little superficial relief, even by an efferent pore; internal diameter 0.7–0.9 mm. Male ampullae also internal, the internal diameter being 0.4–0.5 mm.

#### Remarks.

Although the holotype of *Allopora papillosa* is a small, worn colony measuring only 9.4 × 6.6 mm in width and containing only 28 cyclosystems (Fig. 27D), it is not difficult to see the similarity to the two syntypes of *Allopora petrograpta* Fisher, 1938 (Fig. 27F), also worn specimens (13 and 16 mm in width, constituting over 100 cyclosystems), even though they were collected from shallow water on opposite sides of the Gulf of Alaska. All of the cyclosystems of the type of *Stylantheca papillosa* have only one gastrostyle, whereas all but four of the cyclosystems of the syntypes of *Stylantheca petrograpta* have one gastrostyle, the other four having two. The more difficult synonymy is with the more southerly species *Stylantheca porphyra* Fisher, 1931, described from shallow water in Carmel Bay, California. *Stylantheca porphyra* is very similar to the encrusting forms of *Stylantheca papillosa*, differing only in usually having more than one gastrostyle per cyclosystem, sometimes as many as 8. Cairns (1983b) reported an average of 3.3 gastrostyles per cyclosystem (mode = 3) for the holotype. Another typical population of *Stylantheca porphyra* is reported herein from Monterey Bay (USNM 1084662), also having multiple gastrostyles per cyclosystem, but just 50 km to the north (USNM 1084663) are colonies otherwise identical to *Stylantheca porphyra* that have only one gastrostyle per cyclosystem. Fisher (1938: 530) was uncertain as to the systematic importance of the number of gastrostyles per cyclosystems, suggesting that this character might have subgeneric or even generic discriminating power. We suggest that it is a matter of intraspecific variation and thus synonymize *Stylantheca porphyra* with *Stylantheca papillosa*, resulting in only one valid species in the genus *Stylantheca*. The number of dactylopores per cyclosystem of *Stylantheca porphyra* ranges from 6 to16 (n = 34, average = 9.11, σ = 2.08, mode = 10) (Cairns 1983b), whereas it is lower for the type of *Stylantheca petrograpta* (n = 30, range =2–7, average = 5.07, σ = 0.94 , mode = 5) and slightly lower still for the types of *Stylantheca papillosa* (n = 14, range = 3-5, average and mode= 4.0, σ = 0.68). Another typical specimen (AB76-55) having only 1 gastrostyle per cyclosystem has a range of 5-12 dactylopores per cyclosystem (n = 30, average = 7.43, σ = 1.61, mode = 8). The higher number of dactylopores per cyclosystem of *Stylantheca porphyra* is explained by its larger cyclosystems that house multiple gastrostyles, but in general the number of dactylopores per cyclosystem cannot be used as a diagnostic character for this species. Southern populations that have a slightly larger cyclosystems and more than one gastrostyle per cyclosystem can be referred to as the “*porphyra*” form, a conclusion first suggested by Cairns (1983b: 483). The type of *Stylantheca porphyra* is illustrated by Cairns (1983b) and as Fig. 27G herein.

Two other eastern Pacific species are similar to *Stylantheca papillosa* in color and cyclosystem and gastrostyle shape: *Stylaster venustus* (Verrill, 1870) and *Stylaster californicus* (Verrill, 1866). *Stylantheca venustus* (Figs 16A-I, 17F–G) is known from Monterey Bay, California (USA) to Vancouver Island, BC (Canada) at depths of 10-108 m and, although it may originate with an encrusting base (Fig. 17F, MCZ 5525, holotype), it quickly forms small colonies with delicate branches (Fig. 17G). Sixteen locality records are present at the NMNH, most from off Washington and Oregon (USA), but this species is not treated in this review except to illustrate some of its characters (Table 2). On the other hand, the more southern *Stylantheca californicus*, known from off southern California (USA) from approximately 21–45 m (see Boschma 1957), forms large colonies with robust branches; this species is not further discussed in this review except for the table of comparisons (Table 2).

#### Distribution.

Widespread from the Alaska Peninsula (Shumagin Islands) to Monterey Bay, California, although not yet reported from the northern Gulf of Alaska. Common in the inner passages of Alaska and British Columbia; intertidal to 27 m. Forma *porphyra* known only from Monterey Bay area.

**Figure 28.**
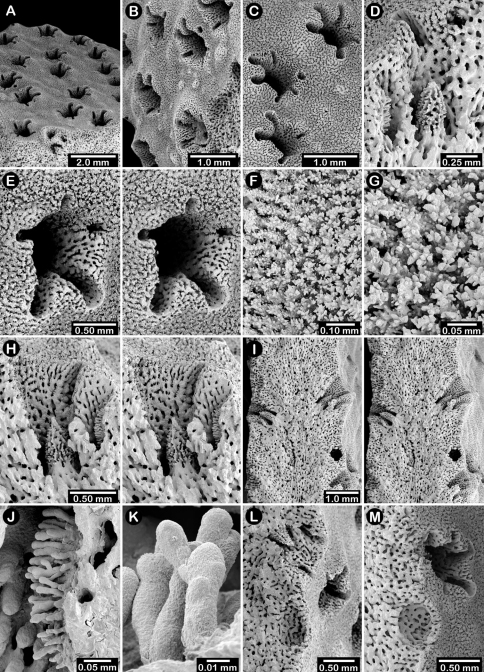
*Stylaster repandus*, **A–K, M** holotype (male), USNM 1122740, **L** female paratype, USNM 1122739: **A–C** cyclosystems and coenosteal papillae **D** gastrostyle and robust dactylostyle **E** stereo view of a cyclosystem **F–G** coenosteal texture **H** stereo view of a gastrostyle and adjacent dactylostyle **I** stereo view of plate cross section showing some gastrostyles and male ampulla **J** a robust dactylostyle **K** elements of a dactylostyle **L** cross section of a female ampulla **M** cross section of a male ampulla.

**Figure 29.**
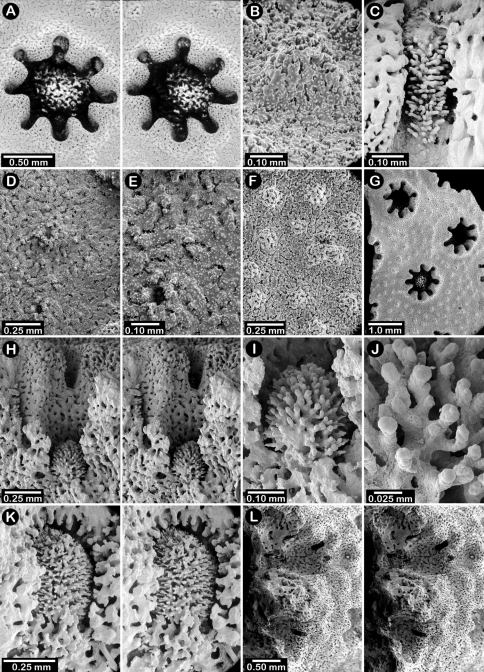
*Stylantheca papillosa*, **A–G, K** topotypic specimen, USNM 43276, **H–J** syntype of *Allopora petrograpta*, USNM 43272, **L** female colony, AB76–55: **A** stereo view of a cyclosystem **B, D, F** coenosteal papillae (nematopores) **C** robust dactylostyle **E** supernumerary dactylopore **G** three cyclosystems and many papillae **H** stereo view of longitudinal section through cyclosystem **I** gastrostyle **J** gastrostyle spines **K** stereo view of squat gastrostyle and ring palisade **L** stereo view of longitudinal section of two cyclosystems and a female ampulla.

### Genus Crypthelia

Milne Edwards & Haime, 1849

http://species-id.net/wiki/Crypthelia

#### Diagnosis.

Colonies usually uniplanar and quite delicate. Coenosteum linear-imbricate and white; nematopores common. Gastro- and dactylopores arranged in cyclosystems that are arranged unifacially on colony, only rarely on both faces; cyclosystems covered all or in part by a fixed lid. Gastropore tube double-chambered, lacking gastro- and dactylostyles. Ampullae usually superficial with a variety of locations of efferent pores.

#### Discussion.

*Crypthelia* is the second-most species-rich genus in the family, currently boasting 31 Recent and one fossil species (Appeltans et al. 2011: www.marinespecies.org). They are easily recognized by the prominent fixed lid that covers each cyclosystem. They are more characteristic of deep water environment and hold the record for the deepest stylasterid species.

#### Type species.

*Crypthelia pudica* Milne Edwards and Haime, 1849, by monotypy.

#### Distribution.

Eocene to Recent: cosmopolitan, but not off continental Antarctica, 183-2789 m.

### Crypthelia trophostega

Fisher, 1938

http://species-id.net/wiki/Crypthelia_trophostega

Figs 27H–I 30A–L

Crypthelia trophostega Fisher, 1938: 533-535, pl. 62, figs 1-8, pl. 63.—Cairns 1986: 24 (ampulla code); 1991: 386 (off Pribilof Island).—Cairns and Macintrye 1992: 102-103 (mineralogy).—Heifetz 2002: 22 (listed).—Wing and Barnard 2004: 10, 27, fig. 29.—Heifetz et al. 2005: 133, 136 (listed).—Stone and Shotwell 2007: 107 (listed).—Brooke and Stone 2007: 530, figs 2L, 3F.—Jameison et al. 2007: 224 (listed).—Lindner et al. 2008: 3, and supplemental Table S1: 1 (phylogeny and DNA sequences).
Cryptohelia trophostega : Thompson and Chow 1955: 30 (mineralogy).

#### Type material.

Holotype: *Alb*-3480, 1 large dry male colony, 15 cm in width, SEM stubs 1482–1484, USNM 42876 (Fig. 27H). Paratypes: *Alb*-3480, 2 dry colonies, male and female, USNM 52264; *Alb*-3480, 1 female colony in alcohol, USNM 43769; *Alb*-3480, 1 dry colony, CAS 69609.

#### Type locality.

*Alb*-3480: 52°06'N, 171°45'W (Amukta Pass), 517 m.

**Material examined.** *Alaskan Leader* 40, 52°09'18"N, 175°40'42"W, 174 m, 8 Jun 2002, 1 indet., USNM 1137354; *Alaskan Leader* 54–14, 51°44.4'N, 178°16.7'W, 567–680 m, 11 Jun 2002, 1 indet., AB02–29; *Alb*-4771, 54°30'N, 179°17'E, 779 m, 4 Jun 1906, 2 males, USNM 76780; *Alb*-4780, 52°01'N, 174°39'E, 1913 m, 7 Jun 1906, 2 female, USNM 62371; *Alb*-4781, 52°14'30"N, 174°13'E, 882 m, 7 Jun 1906, 3 male, USNM 43770; *Delta* 6230–20–19, 52°28.142'N, 173°35.882'W, 190 m, 8 Jul 2004, 1 female, AB; *Dominator* 971–142, 51°43'10"N, 178°35'E, 215 m, 17 Jul 1997, 1 male, USNM 1123356; *Dominator* 971–181, 51°27'43"N, 178°35'E, 384 m, 27 Jul 1997, 1 indet., USNM 1123354; *Dominator* 971–201, 51°54'28"N, 378 m, 1 Aug 1997, 1 male, USNM 1123355; *Dominator* 971–126, 51°34'16"N, 177°47'36"W, 237 m, 12 Jul 1997, 1 indet. in alcohol, USNM 1123353; *Jason* II-2095–2-9–5, 51°48.682'N, 173°50.070'W, 843 m, 26 Jul 2004, 1 male, AB08–0035; *Jason* II-2099–17–1, 51°30.101'N, 177°02.354'W, 1453 m, 30 Jul 2004, 1 indet. in alcohol, AB09–0029; *Ocean Olympic*, 52°22.5'N, 176°03.5'E, 237 m, 1 female, AB 00–0024; *Patricia Lee*, 51°59'N, 179°30.08'E, 457 m, 1 male, AB00–0042; *Sea Storm* 93, 51°50'59"N, 178°26'02"E, 390 m, 5 Jul 2002, 1 male in alcohol, USNM 1122897; *Sea Storm* 108, 52°11'32"N, 175°28'18"E, 208 m, 8 Jul 2002, 1 male, USNM 1122893; *Sea Storm* 109, 52°17'16"N, 175°20'56"E, 238 m, 8 Jul 2002, 1 female in alcohol, USNM 1122894; *Sea Storm* 133, 52°13'40"N, 176°02'19"E, 148 m, 15 Jul 2002, 1 female and 1 male in alcohol, USNM 1122889–90; *Sea Storm* 138, 52°13'50"N, 175.242'E, 146 m, 16 Jul 2002, 1 female and 1 male, USNM 1122891–92; *Sea Storm* 148, 52°28'16"N, 173°19'10"W, 194 m, 2 female, SEM stub 1485, USNM 1122887–88; *Sea Storm* 150, 52°30'47"N, 173.2935°W, 220 m, 21 Jul 2002, 1 female, USNM 1075952; *Sea Storm* 151, 52°33'40"N, 173.3195°W, 203 m, 21 Jul 2002, 1 male, USNM 1122895; *Sea Storm* 155, 52°38'43"N, 172°16.38'W, 393–401 m, 22 Jul 2002, 1 indet., USNM 1122896; *Shishaldin*, 54°12'37"N, 179°30'05"E, 179 m, 1 female and 1 male, USNM 1122435and 1122446; *Shishaldin*, 54°07'N, 179°45'E, 366 m, 20 Feb 2000, 1 female, SEM stubs 1486–87, USNM 1122487; *Vesteraalen* 941–138, 51°28'N, 178°38'W, 0–311 m, 7 Jul 1994, 1 male, USNM 96248; *Vesteraalen* 941–167, 51°54'N, 178°20'E, 0–150 m, 19 Jul 1994, 1 female, USNM 96240; 51°50'32"N, 176°00'08"E, 272 m, 5 Dec 2000, 1 male, USNM 1122441; “Gulf of Alaska", 219–274 m, 1 female, USNM 77415; “Bering Sea", 1 male in alcohol, USNM 76570; University of Washington, 51°32'N, 179°15'W, 278–289 m, 1 Sep 1968, 1 female, USNM 62372.

#### Description.

**Figure 30.**
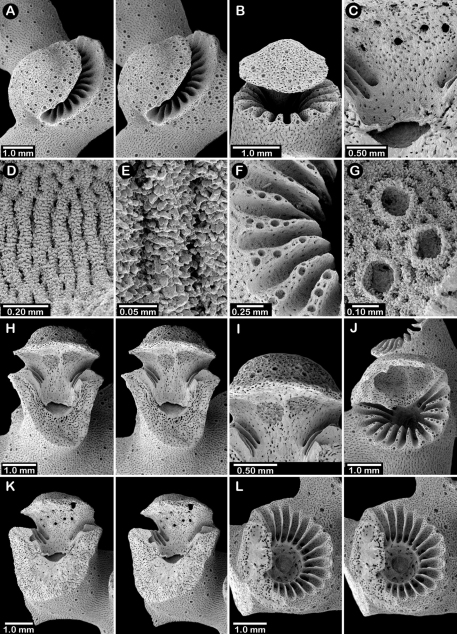
*Crypthelia trophostega*, **A–B, F, H–J** female colony, USNM 1122887 **C–E, G, K–L** holotype (male), USNM 42876: **A–B** cyclosystem and lid, covered with numerous nematopores **C** under side of cyclosystem lid showing numerous male efferent pores **D–E** coenosteal texture **F** slender pseudosepta bearing nematopores **G** three nematopores **H** stereo view of longitudinal section of cyclosystem showing multiple female efferent pores under lid **I** enlargement of efferent pores of figure H **J** fractured female lid **K** stereo view of fractured cyclosystem showing multiple male efferent pores (enlarged in fig. C) **L** stereo view of cyclosystem missing its lid revealing several male ampullae.

Colonies variable in shape, usually uniplanar (Fig. 27H) but sometimes forming three dimensional bushes (Fig. 27I) or multiplanar colonies. Largest colony known (USNM 1122446) is multiplanar, 20 cm tall and 12 cm wide, with a basal diameter of 11.5 mm, this colony only slightly larger than the holotype. Branching irregularly dichotomous and often anastomotic, which reinforces strength of a uniplanar colony. Coenosteum linear-imbricate in surface texture, the parallel strips 50–70 µm wide. Strips covered with irregularly shaped platelets, which are rarely continuous across a strip but dissected into 2–5 smaller sections, each of which has an irregular leading edge and only a slight imbricating overlap with more distal platelet, altogether producing a rough microtexture. Shallow, circular (0.06–0.13 mm in diameter) nematopores very common, occurring on branch coenosteum , cyclosystem lids, and even on pseudosepta (Fig. 30A–C, F–G, J), most concentrated around cyclosystem edge. Nematopores flush with coenenchyme or encircled with a very low rim. Coenosteum white.

Cyclosystems unilinearly arranged either bifacially or on one face, both conditions sometimes occurring on same colony. Unifacially arranged cyclosystems seem to be more common on uniplanar colonies, whereas the bifacial arrangement favors bushy coralla. Cyclosytems circular to slightly elliptical in outline (2.2–2.6 mm in diameter), the greater axis of ellipse being perpendicular to branch axis. Cyclosystems slightly flared, each covered with a horizontal lid that covers most of cyclosystem, sometimes even fusing to opposite side of cyclosystem. Lids usually greatly inflated, containing the male or female ampullae. Cyclosystems have a range of 13–23 dactylopores (n = 50, average = 18.66 (σ=1.96), and mode = 19). Upper gastropore chamber spherical, about 0.9-1.0 mm in diameter, which is separated from lower, flattened chamber by a circular gastropore ring constriction 0.4–0.6 mm in diameter, the lower chamber being about 0.8–0.9 mm in diameter (Fig. 30H). Dactylotomes uniform in width, 0.14-0.16 mm; pseudosepta quite slender, having a thin (20–60 µm) blade-like inner edge (Fig. 30F). Upper edges of pseudosepta not exsert, but gradually attenuate in size near cyclosystem edge.

Female ampullae (Fig. 30H–J) smooth, massive, hemispherical swellings in cyclosystem lid, often two ampullae contained in same lid (Fig. 30I). Efferent pores quite large (up to 0.5 mm in diameter), opening beneath lid (type A ampulla formula of Cairns 1986, Fig. 30H–I). Male ampullae (Fig. 30K–L) irregularly shaped swellings in cyclosystem lid, up to 11 occurring in one lid. Efferent pores smaller (10–12 um in diameter), also opening beneath the lid (type A2 ampulla formula of Cairns 1986)(Fig. 30C, K).

#### Remarks.

Among the 31 Recent species of *Crypthelia* (Cairns et al. 1999; www.marinespecies.org), *Crypthelia trophostega* is unique in having a tendency to have a bifacial cyclosystem arrangement and for having more than one female ampullae per cyclosystem lid. Furthermore, only one other species is known to have the A–A2 ampulla formula (see Cairns 1986), that being *Crypthelia pudica* Milne Edwards and Haime 1849. *Crypthelia trophostega* differs from *Crypthelia pudica* in having bifacial cyclosystems, a rougher coenosteal texture, more nematopores, larger cyclosystems, and lower lids. The corallum was found to be 100% aragonitic according to Cairns and Macintrye (1992). Of the 42 specimens examined: 18 are female, 18 male, and 6 indeterminate in gender.

#### Distribution.

Aleutian Islands from Near Islands to Amuka Pass, Petrel and Bowers Banks, off Pribilof Bank; 146-1913 m, although most records between 200 and 400 m.

## Supplementary Material

XML Treatment for Distichopora

XML Treatment for Distichopora borealis

XML Treatment for Cyclohelia

XML Treatment for Cyclohelia lamellata

XML Treatment for Errinopora

XML Treatment for Errinopora fisheri

XML Treatment for Errinopora undulata

XML Treatment for Errinopora disticha

XML Treatment for Errinopora zarhyncha

XML Treatment for Errinopora nanneca

XML Treatment for Errinopora dichotoma

XML Treatment for Stylaster

XML Treatment for Stylaster brochi

XML Treatment for Stylaster stejnegeri

XML Treatment for Stylaster verrillii

XML Treatment for Stylaster repandus

XML Treatment for Stylaster campylecus

XML Treatment for Stylaster leptostylus

XML Treatment for Stylaster trachystomus

XML Treatment for Stylaster parageus parageus

XML Treatment for Stylaster parageus columbiensis

XML Treatment for Stylaster alaskanus

XML Treatment for Stylaster elassotomus

XML Treatment for Stylaster crassiseptum

XML Treatment for Stylantheca

XML Treatment for Stylantheca papillosa

XML Treatment for Crypthelia

XML Treatment for Crypthelia trophostega
